# Hypergraph representations of single-cell RNA sequencing data for improved cell clustering

**DOI:** 10.1093/bioinformatics/btag148

**Published:** 2026-03-27

**Authors:** Wan He, Daniel I Bolnick, Samuel V Scarpino, Tina Eliassi-Rad

**Affiliations:** Network Science Institute, Northeastern University, Boston, MA 02115, United States; Department of Ecology and Evolutionary Biology, University of Connecticut, Storrs, CT 06269, United States; Network Science Institute, Northeastern University, Boston, MA 02115, United States; Institute for Experiential AI, Northeastern University, Boston, MA 02115, United States; Bouve College of Health Sciences, Northeastern University, Boston, MA 02115, United States; Khoury College of Computer Sciences, Northeastern University, Boston, MA 02115, United States; Vermont Complex Systems Institute, University of Vermont, Burlington, VT 05405, United States; Santa Fe Institute, Santa Fe, NM 87501, United States; Network Science Institute, Northeastern University, Boston, MA 02115, United States; Khoury College of Computer Sciences, Northeastern University, Boston, MA 02115, United States; Vermont Complex Systems Institute, University of Vermont, Burlington, VT 05405, United States; Santa Fe Institute, Santa Fe, NM 87501, United States

## Abstract

**Motivation:**

Single-cell RNA sequencing (scRNA-seq) data analysis is often performed using network projections that produce co-expression networks. These network-based algorithms are attractive because regulatory interactions are fundamentally network-based and there are many tools available for downstream analysis. However, most network-based approaches have two major limitations. First, they are typically unipartite and therefore fail to capture higher-order information. Second, scRNA-seq data are often sparse, so most algorithms for constructing unipartite network projections are inefficient and may overestimate co-expression relationships, or may under-utilize the sparsity when clustering (e.g. with cosine distance). To address these limitations, we propose representing scRNA-seq expression data as hypergraphs, which are generalized graphs where a hyperedge can connect more than two nodes. In this context, hypergraph nodes represent cells, and hyperedges represent genes. Each hyperedge connects all cells in which its corresponding gene is actively expressed, indicating the expression of that gene across different cells. The resulting hypergraph can capture higher-order information and appropriately handle varying levels of data sparsity. This representation enables clustering algorithms to leverage higher-order relationships for improved cell-type differentiation.

**Results:**

To distinguish cell types using hypergraph representations of scRNA-seq data, we introduce two novel clustering algorithms: (i) Dual-Importance Preference Hypergraph Walk (DIPHW) and (ii) Co-expression and Memory-Integrated Dual-Importance Preference Hypergraph Walk (CoMem-DIPHW). DIPHW is a new hypergraph-based random walk algorithm that computes cell embeddings by considering the relative importance of genes to cells and cells to genes, incorporating a preference exponent to facilitate clustering. CoMem-DIPHW integrates two unipartite projections, the gene co-expression and cell co-expression networks, along with the cell–gene expression hypergraph derived from single-cell abundance count data into the random walk model. The advantage of CoMem-DIPHW is that it accounts for both local information from single-cell gene expression and global information from pairwise similarity in the two co-expression networks. We benchmark the performance of our algorithms against established and state-of-the-art deep learning approaches using both real-world and simulated scRNA-seq data. Real-world datasets include cells from the human pancreas, mouse pancreas, human brain, and mouse brain tissues. We also use a ground-truth labeled cell-type annotation dataset based on human lung adenocarcinoma cell lines. Quantitative evaluation shows that CoMem-DIPHW consistently outperforms established algorithms and state-of-the-art deep learning algorithms for cell-type clustering. Our proposed algorithms show the greatest improvement on scRNA-seq data with weak modularity. Moreover, CoMem-DIPHW successfully annotates clusters with biologically relevant cell types. Our results highlight the utility of hypergraph representations in the analysis of scRNA-seq data.

**Availability and implementation:**

Our methods are implemented in Python and available on GitHub (https://github.com/wanhe13/CoMem-DIPHW) and archived at Zenodo (https://doi.org/10.5281/zenodo.18927437).

## 1 Introduction

Network analysis has become a popular tool for studying complex systems in biology, reflecting the combinatorial interactions of biomolecules. Its applications include functional analysis (Vazquez *et al.* 2003, Szklarczyk *et al.* 2015, Veres *et al.* 2015, Ashtiani *et al.* 2018) and prediction of interactions in protein–protein interaction (PPI) networks (Lei and Ruan 2013, Murakami *et al.* 2017, Kovács *et al.* 2019), identification of regulators and analysis of pathways in gene regulatory networks (Zickenrott *et al.* 2016, Muzio *et al.* 2021, Coleman *et al.* 2023), disease modeling, prediction, and intervention in epidemiology (Chinazzi *et al.* 2020, Kraemer *et al.* 2020, Davis *et al.* 2021), and identification of cell types in co-expression networks (Langfelder and Horvath 2008, Satija *et al.* 2015, Butler *et al.* 2018, Wolf *et al.* 2018).

However, traditional methods represent biological systems using unipartite networks, which capture relationships only between a single type of entity. Such representations are often not the most natural or information-preserving choice, since many complex systems involve interactions between multiple types of entities. Most often, a unipartite network projection is chosen such that the system can be analyzed with the abundant network analysis methods developed for unipartite networks (Langfelder and Horvath 2008, Horvát and Zweig 2012). However, this focus on unipartite networks restricts our attention to biological interactions just between pairs of genes, proteins, or other kinds of nodes (We use the terms node and vertex interchangeably.). In reality, biological processes often entail multiway interactions that cannot be represented by unipartite networks. For instance, three-gene epistatic interactions vastly outnumber pairwise epistasis in experimental analyses of yeast genetic networks (Kuzmin *et al.* 2018). In PPI networks, unipartite representations capture relationships between proteins but fail to capture the biological functions associated with groups of proteins. Similarly, unipartite projections of gene regulatory networks connect genes based on regulatory relationships but do not capture relationships among different entities such as transcription factors or their binding sites. Therefore, network analysis methods are needed to more accurately capture the higher-order interactions that are apparently ubiquitous in biological systems.

Single-cell RNA sequencing (scRNA-seq) has enabled the profiling of gene expression at the individual cell level (Picelli *et al.* 2014, Macosko *et al.* 2015, Zheng *et al.* 2017, Yanai and Hashimshony 2019), whereas conventional bulk-tissue RNA sequencing measures expression at the tissue level, averaging gene expression over an ensemble of cells. Cell-type identification, as one of the most important downstream tasks in single-cell RNA-seq (scRNA-seq) data analysis (Hwang *et al.* 2018), has applications in biology and medicine, including tracing the trajectories of different cell lineages in the development of cell differentiation studies (Marioni and Arendt 2017), tissue heterogeneity analysis for cancer research (Karaayvaz *et al.* 2018, Laviron *et al.* 2022, Wong *et al.* 2022), immune cell profiling for therapy development (Ermann *et al.* 2015, Chung *et al.* 2017, Lyons *et al.* 2017, Landhuis 2018), and biomarker discovery for diagnosis.

However, cell-type identification often relies on conventional transcriptomic data analysis pipelines such as WGCNA (Langfelder and Horvath 2008), Scanpy (Wolf *et al.* 2018), and Seurat (Satija *et al.* 2015, Butler *et al.* 2018). These packages embed the cell–gene interactions to a unipartite graph structure such as the co-expression network or the K-nearest neighbor (KNN) graph, which draws edges between the cell pairs based on their similarity, followed by unipartite graph partitioning algorithms to detect closely related cell clusters for cell-type identification in the scRNA-seq data. These unipartite projections of the scRNA-seq expression data have two limitations: Firstly, cell and gene co-expression networks capture only pairwise expression similarity among cells or genes. As a result, higher-order information, such as the expression level of a specific gene in a specific cell or the coordinated expression of multiple genes within the same cell, is lost. Secondly, scRNA-seq data are sparse, with non-zero entries often accounting for less than 10% of the total (Huang *et al.* 2018, Jiang *et al.* 2022), compared to 60%–90% in bulk-tissue data. Despite this sparsity, constructing a unipartite co-expression network produces in a fully-connected network, which is an inefficient representation of the originally sparse transcriptomic data. Furthermore, the high sparsity of scRNA-seq data (both dropouts and biological zeros) results in inflated correlations, leading to spurious connections and obscure meaningful biological signals, as will be discussed further in Section 3.3. In addition, determining whether zero inflation in scRNA-seq data is due to technical dropouts or true biological absence is a challenging task (Kim *et al.* 2020, Qiu 2020, Jiang *et al.* 2022).

**Figure 1:**
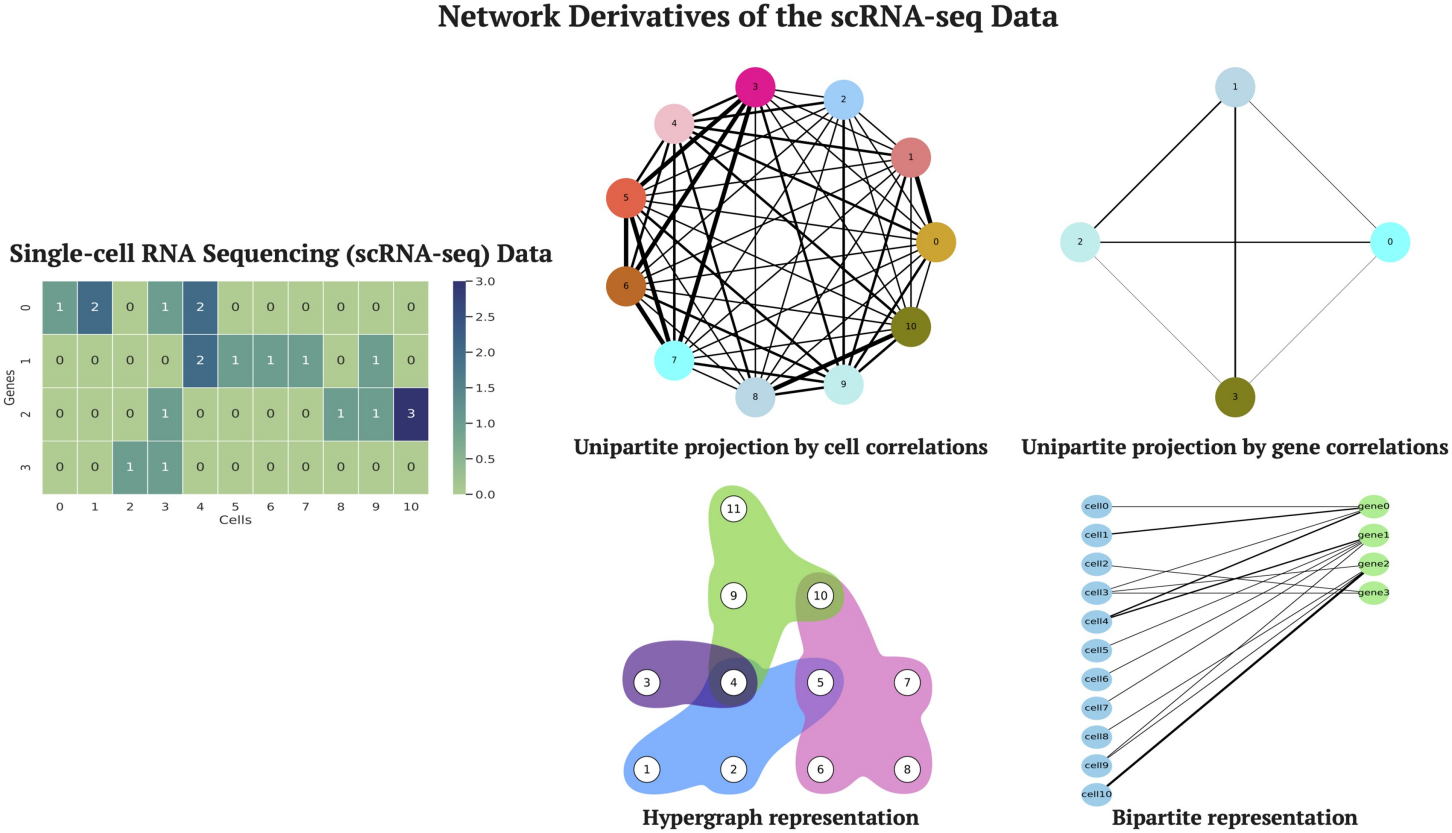
Network representations of scRNA-seq data. Expression data can be represented by various networks, including unipartite network projections such as cell and gene co-expression networks. In a cell co-expression network, nodes are cells and edge weights represent similarity in cell-pair expression. In gene co-expression networks, nodes represent genes. Hypergraph and bipartite network representations preserve the exact expression level of a gene in a cell. In the hypergraph representation, nodes represent cells and hyperedges represent genes. In the bipartite representation, one set of nodes represents cells and the other set represents genes.

**Figure 2:**
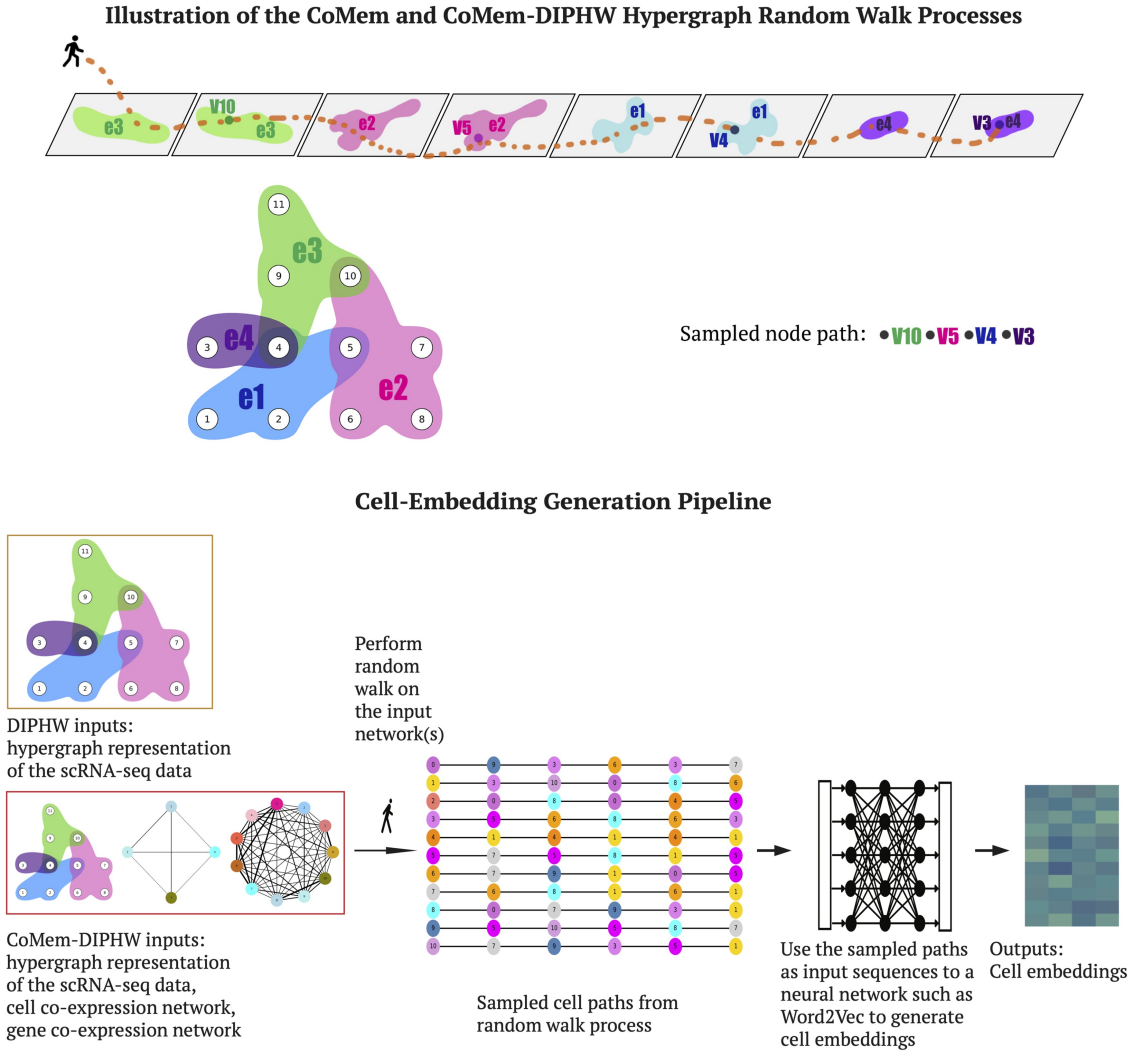
Hypergraph clustering algorithm illustration. In the hypergraph representation of scRNA-seq data, nodes represent cells and edges represent genes. The expression level of a gene in a cell is captured by the weight of a node in an edge. Random walks are performed on the hypergraph by alternately considering the probability of the walker choosing a node given an edge and choosing an edge given the current node. For DIPHW, the input is the hypergraph representation of the scRNA-seq data. For CoMem-DIPHW, the input includes the hypergraph, cell co-expression network, and gene co-expression network, which serve as node and edge similarity matrices to incorporate memory during the walk. A small neural network (word2vec) is applied to the sampled node paths from the hypergraph random walk process to compute cell embeddings. Finally, cell clustering is performed by applying *K*-means to the resulting cell embeddings.

Given the issues with unipartite projections, it is crucial to explore alternative network representations that can accurately reflect the scRNA-seq data and the underlying biological reality of higher-order interactions among genes. Hypergraphs (Battiston *et al.* 2020, 2021, Bianconi 2021) offer a nice solution by directly representing multiway relationships in scRNA-seq data, without requiring further data projection. Existing work on hypergraph representations has demonstrated the improved performance they can achieve in modeling complex biological systems by incorporating higher-order interactions. For example, Liu *et al.* (2022) developed a hypergraph-based neural network to predict synergistic drug combinations for cancer treatment. Wang *et al.* (2024) integrated multi-omics data with hypergraph convolutional neural networks to classify patients with diseases such as breast cancer and Alzheimer’s. Gaudelet *et al.* (2018) used hypergraphs to model interactions across different levels of protein organization (such as PPIs, complexes, and pathways) to better capture the complexity of biological systems and predict biological functions. Ma and Ma (2022) developed a hypergraph-based logistic matrix factorization method that predicts possible metabolite—disease interactions and uncovers novel disease-related metabolites. These successes highlight the potential of hypergraphs to model multiway relationships inherent in biological systems, motivating their application to scRNA-seq analysis (see Figs 1 and 2).

## 2 Materials and methods

We conceptualize scRNA-seq data as a hypergraph H and design two hypergraph random walk algorithms that capture cell–cell relationships by sampling cell-to-gene and gene-to-cell interactions in the hypergraph. This hypergraph representation of scRNA-seq data does not require additional data transformation, does not cause information loss, or increase computational cost (see Table 1 for notation summary).

**Table 1:** Notation table for our hypergraph random walk representation.

Notation	Description
H	Hypergraph
$I_{H}$	Incidence matrix of the hypergraph H
*V*	Set of nodes (cells)
*E*	Set of hyperedges (genes) in the hypergraph
$G_{V}$	Cell co-expression network (node similarity matrix)
$G_{E}$	Gene co-expression network (edge similarity matrix)
$\Gamma_{e}(v)$	Weight of node *v* in hyperedge *e* (i.e. expression of gene *e* in cell *v*)
P(V=v∣E=e)	Probability of selecting node *v* given hyperedge *e*
P(E=e∣V=v)	Probability of selecting hyperedge *e* given node *v*
$P(E_{t+1}=e^{\prime }|E_{t}=e,V_{t}=v)$	Probability of transitioning to hyperedge $e^{\prime }$ at time t+1, given current hyperedge *e* and node *v* at time *t*
$P(V_{t+1}=w|E_{t+1}=e,V_{t}=v)$	Probability of selecting node *w* at time t+1, given hyperedge *e* at time t+1 and previous node *v*
$P(V_{t+1}=w|V_{t}=v)$	Unipartite node-to-node transition probability

### 2.1 Hypergraph representation

scRNA-seq data can be conceptualized as a hypergraph H=(V,E), where

- *V* represents the set of nodes corresponding to the cells profiled in the experiment,
- *E* denotes the set of hyperedges representing genes, with each hyperedge e∈E connecting to all cells ∈V in which the gene *e* is actively expressed. The weight of connection of a hyperedge (gene) to each node (cell) is determined by the gene’s expression level in that cell.

Thus, each hyperedge captures the expression profile of a gene across all cells, preventing any loss of information. The incidence matrix $I_{H}$ of the hypergraph H is constructed such that $I_{H}(v,e)$ is given by the expression level, i.e. abundance counts, of gene *e* in cell *v*.

### 2.2 Method 1: dual-importance preference hypergraph walk

Building on this hypergraph representation, we introduce a novel hypergraph random walk approach for cell embedding computation, the Dual-Importance Preference (DIP) Hypergraph Walk (DIPHW), which accounts for both the relative importance of edges to nodes and nodes to edges, or the importance of genes relative to cells and cells to genes, along with a preference exponent for faster clustering. In a hypergraph random walk process, the walker alternates between transitioning from a hyperedge to a node and from a node to a hyperedge, following the node-to-edge transition probabilities $P_{E|V}(e,v)$ and edge-to-node transition probabilities $P_{V|E}(v,e)$. In the hypergraph random walk with edge-dependent vertex weight (EDVW) by Chitra and Raphael (2019), the edge-to-node transition probability is edge-dependent, but the node-to-edge transition probability does not consider the importance of the edge to the node. As a natural extension to EDVW, we define the node-to-edge transition probability to be vertex-dependent in DIPHW and use a preference exponent to accelerate the clustering process. The modified random walk process is as follows:

1. *Node-to-hyperedge transition probability*: The probability of selecting hyperedge $e_{t+1}$ given that the walker is currently at vertex $v_{t}$ is defined as:
  $$P_{E|V}(v_{t}\to e_{t+1})=\frac{\omega (e_{t+1})\gamma_{e_{t+1}}(v_{t})}{\sum_{e^{\prime }\in E(v_{t})}\omega (e^{\prime })\gamma_{e^{\prime }}(v_{t})}$$
  In scRNA-seq cell clustering, this represents the probability of selecting a gene based on its expression level relative to other genes in the same cell.
2. *Hyperedge-to-node transition probability*: Upon the walker’s arrival at hyperedge $e_{t+1}$, the probability of moving to vertex $v_{t+1}\in e_{t+1}$ is given by:
  $$P_{V|E}(e_{t+1}\to v_{t+1})=\frac{\gamma_{e_{t+1}}^{\alpha }(v_{t+1})}{\sum_{v^{\prime }\in e_{t+1}}\gamma_{e_{t+1}}^{\alpha }(v^{\prime })}$$
  In scRNA-seq cell clustering, the transition from a gene to a cell is based on the relative expression level of a gene in a cell, amplified by a preference exponent to improve clustering convergence.
3. *Node-to-node transition probability*: The unipartite node-to-node transition probability of DIPHW is calculated by summing the products of the node-to-edge and edge-to-node transition probabilities across all hyperedges. This probability is used to sample random walk node paths, which are then input into a neural network to compute cell embeddings
  $$P(v_{t}\to v_{t+1})=\sum_{e\in E}P_{E|V}(e|v_{t})P_{V|E}(v_{t+1}|e).$$
  Substituting the expressions for $P_{E|V}(e_{t+1}|v_{t})$ and $P_{V|E}(v_{t+1}|e_{t+1})$, we get:
  (1) $$\begin{array}{cc} P(v_{t}\to v_{t+1}) & =\sum_{e_{t+1}\in E}\frac{\omega (e_{t+1})\gamma_{e_{t+1}}(v_{t})}{\sum_{e^{\prime }\in E}\omega (e^{\prime })\gamma_{e^{\prime }}(v_{t})}\\ & \;\times \frac{\gamma_{e_{t+1}}^{\alpha }(v_{t+1})}{\sum_{v^{\prime }\in e_{t+1}}\gamma_{e_{t+1}}^{\alpha }(v^{\prime })} \end{array}$$
  The expression of node-to-node transition probability could be simplified by removing reference to any particular time points as,
  $$P(u\to v)=\sum_{e\in E(u)}\frac{\omega (e)\gamma_{e}(u)}{\sum_{e^{\prime }\in E}\omega (e^{\prime })\gamma_{e^{\prime }}(u)}\frac{\gamma_{e}^{\alpha }(v)}{\sum_{u^{\prime }\in e}\gamma_{e}^{\alpha }(u^{\prime })}$$
4. *Node-to-node transition probability in vectorized format*: The |V|×|V| node-to-node transition probability matrix $P_{V|V}$ could be written in the matrix form as:
  $$P=D_{E|V}^{-1}W_{E|V}D_{V|E}^{-1}W_{V|E}$$

The matrix formulation eliminates explicit iteration and reduces computation time. Here, $W_{E|V}=I_{H}^{T}W_{E}$ is the |V|×|E| node-to-edge transition weight matrix, where column *e* of $W_{E|V}$ contains the vertex weights in hyperedge *e* multiplied by the weight of hyperedge *e*. Similarly, $W_{V|E}$ represents the edge-to-node transition weight matrix before normalization with dimensions |E|×|V|, given by $W_{V|E}=I_{H}^{\alpha },\;w_{ve}={\left[I_{H}(e,v)\right]}^{\alpha }$. Finally, $D_{E|V}$ and $D_{V|E}$ are diagonal matrices that normalize vertex-to-edge and edge-to-vertex transitions to ensure the probability distribution sums to 1.

### 2.3 Method 2: co-expression and memory-integrated dual-importance preference hypergraph walk (CoMem-DIPHW)

CoMem-DIPHW is an extension of DIPHW. It incorporates co-expression networks and single-cell transcriptomic profiles. The memory mechanism of CoMem-DIPHW leverages cell and gene co-expression networks to account for previously visited nodes and edges during transitions to capture both local expression relationships and global co-expression patterns.

1. *Hypergraph representation of the scRNA-seq data*: We note that a matrix can be directly interpreted as the incidence matrix of a hypergraph to represent the relationship between two variables, without additional computation.
2. *Node and edge similarity networks*: Using scRNA-seq data, we construct two co-expression networks: the cell co-expression network ($G_{V}$) and the gene co-expression network ($G_{E}$). In the cell co-expression network, cells are nodes, and edges represent correlation coefficients between cell pairs, indicating the similarity of their expression profiles. Similarly, in the gene co-expression network, genes are nodes, and edges represent correlation coefficients between gene pairs. We use Spearman’s rank coefficient to measure pairwise similarity of expression profiles, as it captures non-linearity better than Pearson’s correlation. These unipartite networks serve as node and edge similarity matrices, embedding global-level information that summarizes associations between cell and gene pairs.
3. *Memory-integrated random walk*: CoMem-DIPHW incorporates a memory component by considering the similarity between consecutively visited nodes and edges. This memory mechanism adjusts the transition probabilities so that the node-to-edge transition probability depends on the previously visited edge, and the edge-to-node transition probability depends on the previously visited node. Node–node and edge–edge dependencies are incorporated through the node and edge similarity networks, which are two correlation networks derived from transcriptomic expression data. The memory mechanism constrains the hypergraph walker to focus on cell–gene interactions among similar nodes (cells) and edges (genes), thus better revealing modularity within the data. While the co-expression networks provide global perspectives through unipartite projections, the specific expression levels of genes in individual cells (local information) are preserved in the hypergraph structure. CoMem-DIPHW integrates global and local information for more accurate cell-type identification.
4. *Formulation*: The random walk process in CoMem-DIPHW is formulated as follows. Let $G_{V}$ be the vertex similarity matrix, where $G_{V}(v,w)$ quantifies the similarity between nodes *v* and *w*. Similarly, let $G_{E}$ be the edge similarity matrix, where each element $G_{E}(e,f)$ represents the similarity between edge *e* and *f*.

#### 2.3.1 Method 2.0: CoMem

In CoMem, we calculate the unipartite node-to-node transition probability of the hypergraph random walker. To do this, we first calculate the following four probabilities.

1. The probability of selecting vertex *v* in edge *e* is:
  $$P_{V|E}(e\to v)=\frac{\Gamma_{e}(v)}{\sum_{u\in e}\Gamma_{e}(u)}$$
2. The probability of selecting edge *e* given vertex *v* is:
  $$P_{E|V}(v\to e)=\frac{\Gamma_{e}(v)}{\sum_{e\in E}\Gamma_{e}(v)}$$
3. The probability of transitioning from node *v* in edge $e_{1}$ to edge $e_{2}$, considering edge similarity, is given by:
  $$P_{E_{t+1}|V,E_{t}}(e_{2}|v,e_{1})=\frac{G_{E}(e_{1},e_{2})P(e_{2}|v)}{\sum_{e^{\prime }\in E}G_{E}(e_{1},e^{\prime })P(e^{\prime }|v)}$$
4. Similarly, the probability of selecting node *w* from edge *e* after transitioning from node *v*, incorporating node similarity and the node-edge connection, is formulated as:
  $$P_{V_{t+1}|E,V_{t}}(w|e,v)=\frac{G_{V}(v,w)P(w|e)}{\sum_{w^{\prime }\in e}G_{V}(v,w^{\prime })P(w^{\prime }|e)}$$

Finally, the unipartite node-to-node transition probability of the hypergraph random walk process is:


$$\begin{array}{cc} & P_{V_{t}\to V_{t+1}}(v_{1},v_{2})\propto \sum_{e_{1},e_{2}\in E}P_{V|E}(v_{1}|e_{1})\\ & \;\times P_{E_{2}|V,E_{1}}(e_{2}|v_{1},e_{1})\;\times P_{V_{t+1}|E,V_{t}}(v_{2}|e_{2},v_{1}) \end{array}$$

To encourage exploration, we modify the node and edge similarity graphs such that the diagonal elements are set to zero. This ensures non-lazy walks, where the random walker does not remain at the same node or edge in consecutive steps.

#### 2.3.2 Method 2.1: CoMem based on DIPHW

For CoMem-DIPHW, we integrate DIPHW into the CoMem walk framework from Section 2.3. Specifically, the vertex selection probability $P_{V|E}(e\to v)$ and the edge selection probability $P_{E|V}(v\to e)$ are both defined as in DIPHW (Section 2.2), while all other probability distributions remain as specified in Section 2.3.

### 2.4 Cell embedding and clustering

We perform random walks using the defined hypergraph node-to-edge and edge-to-node transition probabilities to sample sequences of visited cells. These sampled cell paths, which capture the geometry of the hypergraph representing the scRNA-seq data, are then used as input to word2vec (Mikolov *et al.* 2013) to learn low-dimensional vector representations of cells (see Fig. 2 for illustration). Cells with similar expression profiles frequently co-occur in the sampled random walk paths and are closer in the embedded space. Finally, *K*-means clustering is applied to cell embeddings to obtain cluster assignments.

### 2.5 scRNA-seq data simulation

In our study, we develop an algorithm to simulate data that mimics the characteristics of scRNA-seq expression data (see Algorithm 1). In addition to the underlying assumption of within-cell-type homogeneous expression that governs most cell-clustering methods, we incorporate between-cell-type crosstalk (Lehuen *et al.* 2010, Zepp and Morrisey 2019, Armingol *et al.* 2021, Bayik and Lathia 2021, Zhang *et al.* 2023) by integrating configurable intermodular covariance, density, and signal strength into the simulation model. The algorithm generates a sparse matrix representing the simulated scRNA-seq expression data, with numerous user-configurable parameters, including number of modules, module density, shape, background signal strength, modular signal strength, intermodular signal strength, intermodular covariance, and noise level (see Table S4, available as supplementary data at *Bioinformatics* online). This method is implemented as a function in our codebase and is available for use by other researchers (He *et al.* 2025).

Our simulation model is highly flexible to accommodate various types of scRNA-seq data. For example, the model can simulate both sequenced datasets with high density (up to 50%) and sequenced datasets with low density (as low as 1%) (Andrews *et al.* 2021). The proportion of non-housekeeping genes can be adjusted by changing the number of differentially expressed genes (DEGs) in each module. In addition, the variance in module sizes and the number of co-expressed genes in each cell type can be configured by setting different variances for the Poisson distribution used in the embedded module simulation. The modules embedded in the simulated data can be configured with higher density and average signal strength to model the expression profiles of biomarker genes that are highly expressed in certain cell types. This enables us to mimic the sparsity and modularity of real scRNA-seq data, where a small number of genes are highly expressed in specific cell types. Alternatively, the embedding modules can be user-specified without using the embedded module simulation.

Algorithm 1. Simulation Algorithm for scRNA-seq Data

1: **Input:** Number of genes *E*, number of cells *V*, number of cell types *K*; average genes per cell type $\bar{g}$ and average cells per cell type $\bar{c}$; average expression levels for within-cell-type $\lambda_{\text{ct}}$, cross-cell-type $\lambda_{\text{cross}}$, and background $\lambda_{\text{bg}}$; densities within-cell-type $\rho_{\text{ct}}$, cross-cell-type $\rho_{\text{cross}}$, and background $\rho_{\text{bg}}$; and crosstalk probability $p_{\text{cross}}$.

2: **Output:** Sparse expression matrix $X\in R^{E\times V}$ with ground-truth labels.

3: Initialize $X\leftarrow 0_{E\times V}$.

4: Sample cell type sizes: $g_{k}∼\text{Poisson}(\bar{g})$, $c_{k}∼\text{Poisson}(\bar{c})$ for k=1,…,K.

5: *Phase 1: Within-Cell-Type Signal*

6: **for** each cell type k=1,…,K  **do** 

7:   For each gene $e\in E_{k}$ and cell $v\in V_{k}$: with probability $\rho_{\text{ct}}$, set $X[e,v]\leftarrow \text{Poisson}(\lambda_{\text{ct}})$.

8: **end for** 

9: *Phase 2: Background Noise*

10: Randomly fill $\rho_{\text{bg}}$ fraction of remaining zero entries with $\text{Poisson}(\lambda_{\text{bg}}$ values.

11: *Phase 3: Cross-Cell-Type Crosstalk*

12: **for** each cell type pair (k,l) where k≠l  **do** 

13:   With probability $p_{\text{cross}}$: randomly fill $\rho_{\text{cross}}$ fraction of $X[E_{k},V_{l}]$ with $\text{Poisson}(\lambda_{\text{cross}})$ values.

14: **end for** 

15: **return**  X, ground-truth labels.

## 3 Experiments and results

### 3.1 Baseline and competing methods

To evaluate the performance of DIPHW and CoMem-DIPHW, we compared them with 13 cell-clustering methods from various categories, including community detection, embedding-based methods, and recent deep learning methods that cluster scRNA-seq data. Table 2 summarizes these methods. For a fair comparison under consistent conditions, we focus on core clustering algorithms rather than specific implementations in packages such as Seurat (Satija *et al.* 2015), Scanpy (Wolf *et al.* 2018), and WGCNA (Langfelder and Horvath 2008). This approach allows us to evaluate the clustering capabilities of each method independently of any additional preprocessing or optimization steps. The hyperparameters used for each experiment are detailed in Table S4, available as supplementary data at *Bioinformatics* online.

**Table 2:** List of clustering methods evaluated in our study.^a^

Method	Type	Input	Packages
Greedy Modularity (Newman 2004)	Community detection	Cell co-expression network	igraph (Csardi and Nepusz 2006)
Louvain (Blondel *et al.* 2008)	Community detection	Cell co-expression network	Seurat (Satija *et al.* 2015), Scanpy (Wolf *et al.* 2018), WGCNA (Langfelder and Horvath 2008)
Infomap (Rosvall and Bergstrom 2008)	Community detection	Cell co-expression network	igraph (Csardi and Nepusz 2006)
Leiden (Traag *et al.* 2019)	Community detection	Cell co-expression network	igraph (Csardi and Nepusz 2006)
PCA (Pearson 1901)	Linear embedding	Gene × cell matrix	Seurat (Satija *et al.* 2015), Scanpy (Wolf *et al.* 2018), SC3 (Kiselev *et al.* 2017), Cell Ranger (Zheng *et al.* 2017)
t-SNE (Van der Maaten and Hinton 2008)	Manifold embedding	Gene × cell matrix	Seurat (Satija *et al.* 2015), Scanpy (Wolf *et al.* 2018)
UMAP (McInnes *et al.* 2018)	Manifold embedding	Gene × cell matrix	Seurat (Satija *et al.* 2015), Scanpy (Wolf *et al.* 2018)
Node2Vec (Grover and Leskovec 2016)	Graph embedding	Cell co-expression network	Multiple implementations available (Grover and Leskovec 2016); SNAP implementation: https://github.com/snap-stanford/snap/tree/master/examples/node2vec
graph-sc (Ciortan and Defrance 2022)	Deep graph embedding	Cell co-expression network	Ciortan and Defrance (2022)
tsImpute (Zheng *et al.* 2023)	Imputation	Gene × cell matrix	Zheng *et al.* (2023)
CAKE (Liu *et al.* 2024)	Deep contrastive clustering	Gene × cell matrix	Liu *et al.* (2024)
scASDC (Min *et al.* 2024)	Deep clustering	Gene × cell matrix	Min *et al.* (2024)
EDVW (Chitra and Raphael 2019) + word2vec (Mikolov 2013)	Hypergraph embedding	Gene × cell matrix	Implemented based on Chitra and Raphael (2019); GitHub (He *et al.* 2025)
DIPHW + word2vec (Mikolov 2013)	Hypergraph embedding	Gene × cell matrix	Our proposed method; GitHub (He *et al.* 2025)
CoMem + word2vec (Mikolov 2013)	Hypergraph embedding	Gene × cell matrix, plus gene and cell co-expression networks	Our proposed method; GitHub (He *et al.* 2025)

a *K*-means was used to cluster the output of all embedding-based methods that do not include a clustering algorithm. Since tsImpute is an imputation method, we evaluated its impact on clustering performance by applying PCA followed by *K*-means to its imputed data.

### 3.2 Data and preprocessing

We use simulated and publicly available scRNA-seq datasets for our study. The simulated data is generated using our simulation algorithm described in Section 2.5, with pseudocode in Algorithm 1 and code at He *et al.* (2025).

For both simulated and real scRNA-seq data, preprocessing includes Counts Per Million (CPM) normalization (Vallejos *et al.* 2017) to account for differences in sequencing depth, log transformation to reduce skewness, and removal of genes and cells with zero total expression. For real scRNA-seq datasets, we additionally retain the top *n* highly variable genes using Scanpy’s implementation of the Seurat (Satija *et al.* 2015) method. The values of *n* used for each dataset are provided in Table S4, available as supplementary data at *Bioinformatics* online.

We use six publicly available scRNA-seq datasets: (i) human brain (Camp *et al.* 2015), (ii) human pancreas (Muraro *et al.* 2016), (iii) mouse brain (Zeisel *et al.* 2015), (iv) mouse pancreas (Baron *et al.* 2016), (v) scMixology benchmark with three classes, and (vi) scMixology benchmark with five classes. scMixology benchmark which is a curated benchmark dataset with ground-truth cell line labels (Tian *et al.* 2019). Table 3 provides a summary of these datasets.

**Table 3:** List of publicly available datasets used in our study.^a^

Datasets	# of cells	# of genes	Sparsity (%)	Results in
scMixology (3-class) (Tian *et al.* 2019)	902	16 468	45.02	Table 4
scMixology (5-class) (Tian *et al.* 2019)	3918	11 786	63.01	Table 4
Human pancreas (Muraro *et al.* 2016)	3072	18 348	78.74	Section 3.6
Human brain (Camp *et al.* 2015)	735	18 929	80.11	Section S6, available as supplementary data at *Bioinformatics* online
Mouse brain (Zeisel *et al.* 2015)	3006	19 973	81.21	Section S7, available as supplementary data at *Bioinformatics* online
Mouse pancreas (Baron *et al.* 2016)	1065	14 881	87.79	Section S7, available as supplementary data at *Bioinformatics* online

a Sparsity measures the percentage of zero entries in a cell × gene matrix.

### 3.3 Sparsity-induced inflated correlation in scRNA-seq co-expression networks

The zero-inflation problem in scRNA-seq data analysis refers to the excessive number of zero counts (or “dropouts”) observed in the data. These zeros may arise from technical limitations in detecting low-abundance transcripts (false negatives) or from the true absence of transcripts (true negatives) (Kim *et al.* 2020, Qiu 2020, Jiang *et al.* 2022). Integrating individual cell expression profiles into a unified scRNA-seq dataset requires constructing a matrix that includes every gene detected in any cell. This process introduces zero entries for genes that are not expressed in specific cells. Because cells typically express different sets of genes, the merged dataset becomes highly sparse. In co-expression network construction, edge weights are inflated due to these artificially introduced common zeros, as the common zeros are interpreted as evidence of expression homogeneity. This issue is especially pronounced at the single-cell level compared to bulk-tissue RNA-seq data, since the proportion of expressed genes per cell is much smaller, resulting in greater data sparsity.

**Figure 3:**
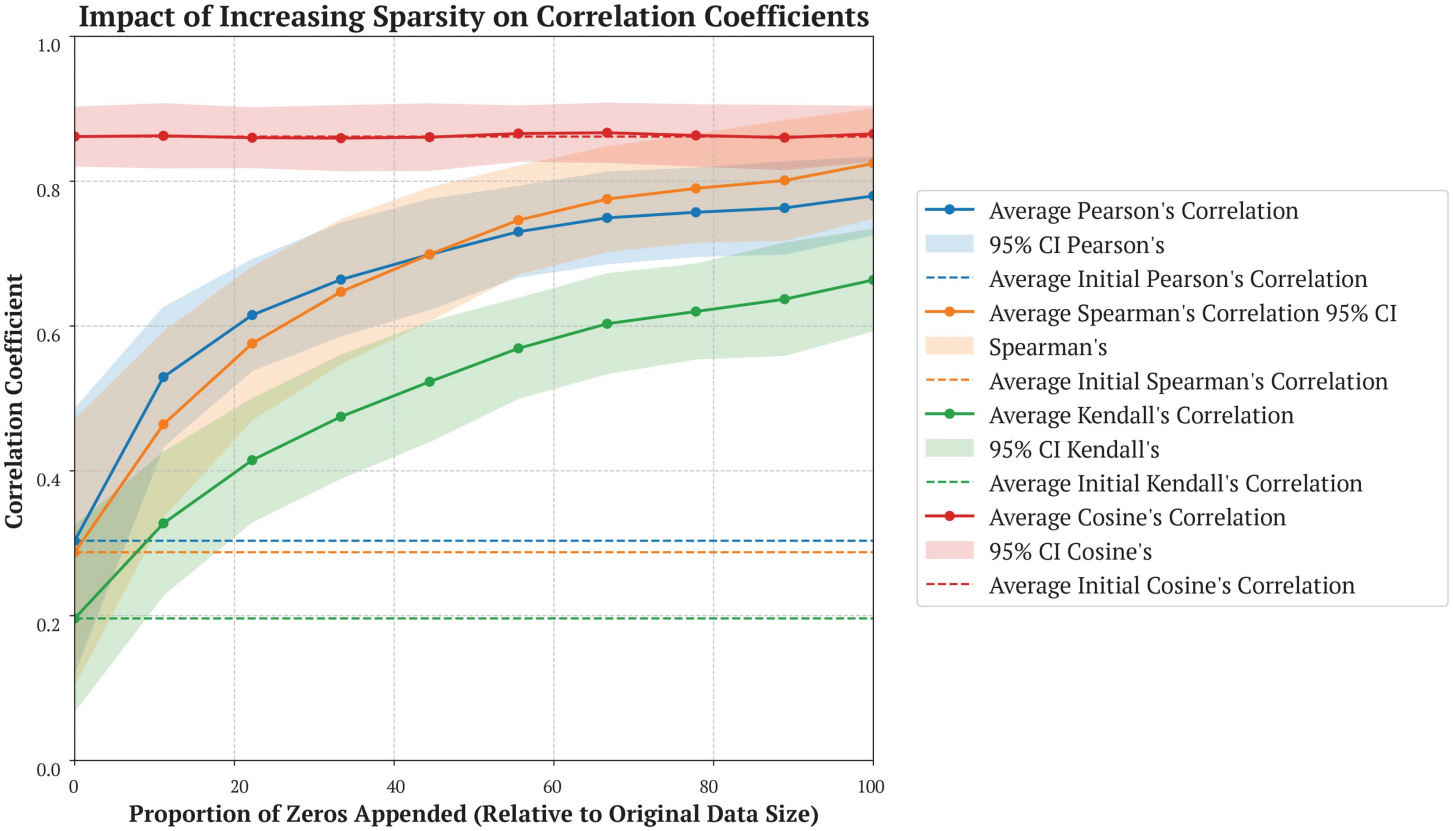
Impact of increased sparsity on correlation coefficients. This figure shows the relationship between the proportion of zeros added to the expression profile (representing increased sparsity) and the resulting correlation coefficients. This process was repeated 100 times. Shaded areas indicate the 95% confidence intervals around the average correlation values. The dashed lines represent the average initial correlation without added sparsity. Induced sparsity in scRNA-seq data can inflate correlations in co-expression networks, highlighting the need for alternative scRNA-seq data representations.

In Fig. 3, we examine how induced sparsity affects correlation coefficients and the interpretation of gene and cell connections in co-expression networks. To simulate this effect, we generate gene expression data with a fixed base size and introduce varying proportions of zeros to represent different sparsity levels. Our results indicate that as induced sparsity increases, correlation coefficients between expression profiles become artificially inflated, leading to an overestimation of similarity between cell expression profiles. These findings highlight the importance of addressing zero inflation in scRNA-seq data analysis and the need for accurate representation of underlying biological relationships.

Table S1 in Section S1, available as supplementary data at *Bioinformatics* online, shows that ignoring shared zeros using cosine similarity results in poorer clustering performance, as these inactive genes still have biological significance.

### 3.4 Results on the simulated data

To evaluate performance of the clustering methods on datasets with known ground-truth, we employ five widely used measures to compute coherence between the identified clusters and the ground-truth clusters: adjusted rand index (ARI), normalized mutual information (NMI), adjusted mutual information (AMI), clustering accuracy (ACC), and *F*1 score. All evaluation measures are invariant to permutations, with a score of 0 representing random labeling and a score of 1 indicating identical clusters. *K*-means is used to cluster the output of all embedding-based methods that do not directly assign cluster membership.

**Figure 4:**
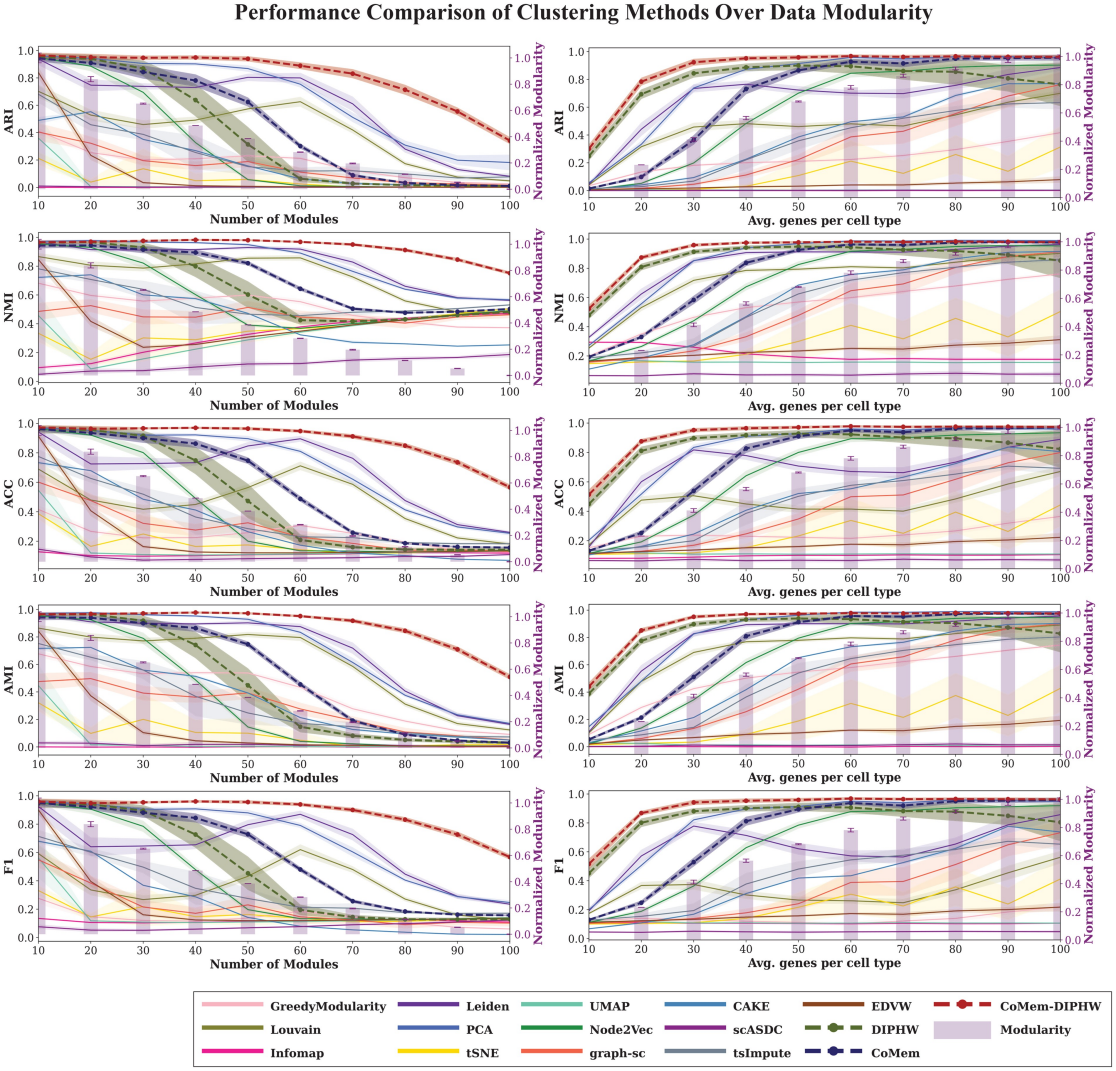
Comparing performances of clustering methods on simulated scRNA-seq data across modularity. The performance of clustering methods is measured by ARI, NMI, ACC, AMI, and *F*1 across different levels of data modularity. The *x*-axis represents parameters determining data modularity, including the average number of co-expressed genes per cell type and the number of embedded modules. Our proposed methods, DIPHW, CoMem, and CoMem-DIPHW, are highlighted with dashed lines. CoMem-DIPHW, in particular, maintains strong performance and is least affected by decreases in modularity, performing significantly better even under conditions of extremely weak modularity. This behavior is observed consistently across all evaluation measures. Experiments are repeated 10 times for each parameter setting, and the 95% confidence intervals (CIs) are shown. The bar plots represent Barber’s bipartite modularity, normalized so that the most modular graphs have modularity = 1. *K*-means is used to cluster the output of all embedding-based methods that do not include an inherent clustering mechanism.

**Figure 5:**
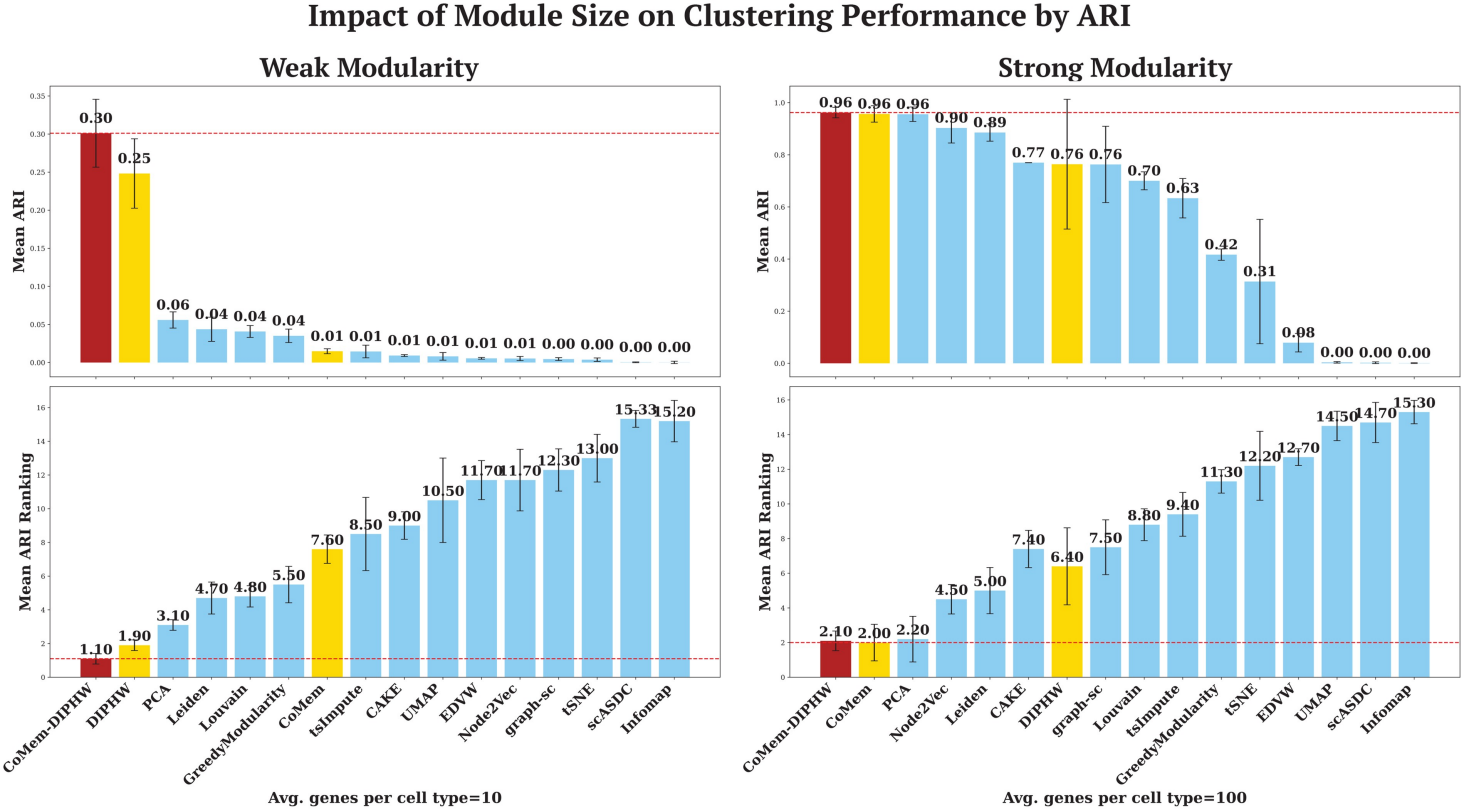
Impact of module size on clustering performance by ARI. The bar plots show the mean ARI (top) and average ranking (bottom) for each method, ordered by their average ARI. Each experiment was repeated 10 times per parameter setting, with error bars representing the 95% confidence interval. Dashed lines indicate the highest ARI values or best ARI rankings. Our proposed hypergraph-based methods (CoMem-DIPHW, CoMem, and DIPHW) show significant advantages when data modularity is weak, i.e. when the average number of co-expressed genes per cell-type module is low (10 genes per module). Under strong modularity conditions, where the average number of genes per cell-type module is high (100 genes per module), many methods (e.g. PCA, CoMem-DIPHW, CoMem, and Node2Vec) perform well and achieve average ARI scores above 0.9. These observations are consistent across NMI, ACC, AMI, and *F*1, as shown in Section S5, available as supplementary data at *Bioinformatics* online. *K*-means is used to cluster the output of all embedding-based methods that do not directly assign cluster membership.

**Figure 6:**
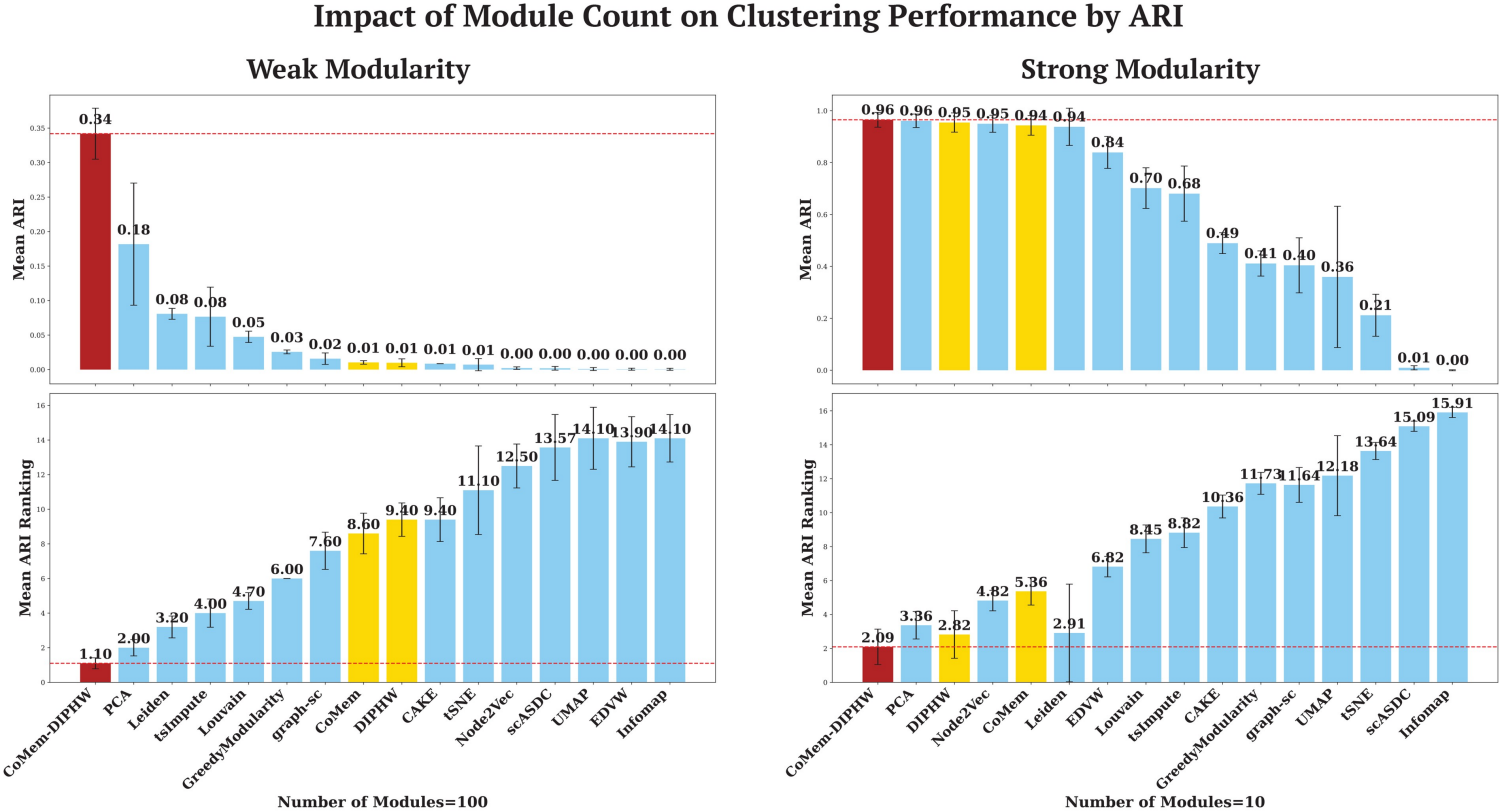
Impact of module count on clustering performance by ARI. The bar plots show the mean ARI (top) and average ranking (bottom) of each method, ordered by their average ARI. CoMem-DIPHW demonstrates significant advantages in weak modularity regimes, where the number of embedded modules (representing the number of cell types) in the simulated data is high (100 modules). Each experiment is repeated 10 times per parameter setting, with error bars representing the 95% confidence interval. Dashed lines indicate the highest ARI values or best ARI rankings. These observations are consistent across NMI, ACC, AMI, and *F*1, as shown in Section S5, available as supplementary data at *Bioinformatics* online. *K*-means is used to cluster the output of all embedding-based methods that do not directly assign cluster membership.

We evaluate the performance of clustering methods under varying data modularity conditions. Modularity is controlled by two key parameters: the average number of co-expressed genes per cell type and the number of embedded cell modules. The number of modules corresponds to the number of cell types in the simulated scRNA-seq data. Weak modularity refers to a small number of co-expressed genes per module or a large number of embedded modules, which makes data structures more difficult to detect. The modularity bar plots in Fig. 4 and the heatmap visualization in Fig. S11, available as supplementary data at *Bioinformatics* online, show that the simulated scRNA-seq data exhibit stronger modularity when the average number of co-expressed genes per cell type is higher or when the number of modules is smaller. We implemented Barber’s bipartite modularity (Barber 2007), as shown in the bar plots in Fig. 4, to quantify the modularity of the underlying data for clustering and to analyse the association between modularity and clustering performance. Clustering performance generally improves as data modularity increases. Our proposed methods, DIPHW, CoMem, and CoMem-DIPHW, demonstrate strong and highly competitive performance across varying modularity. Specifically, CoMem-DIPHW consistently remains competitive with the best-performing methods evaluated, with its advantage particularly pronounced under weak data modularity conditions. For clarity, we present the most contrasting cases from our analysis: (i) an average of 10 versus 100 co-expressed genes per module in Fig. 5 and (ii) 10 versus 100 embedded modules in Fig. 6.

In Figs 5 and 6, we compare the performance of our proposed hypergraph-based clustering methods (highlighted in red and yellow) with five embedding-based approaches, four community detection algorithms, and four state-of-the-art scRNA-seq clustering methods. *K*-means is used to cluster the output of all embedding-based methods that do not directly assign cluster membership. In weak modularity regimes (average number of co-expressed genes per module = 10 or number of modules = 100), CoMem-DIPHW and DIPHW show a clear performance advantage. For example, when the average number of co-expressed genes is 10, CoMem-DIPHW achieves an ARI of 0.30 and DIPHW achieves an ARI of 0.25, compared to 0.05 for the best baseline method (PCA). Under strong modularity conditions (average number of genes per module = 100 or number of modules = 10), multiple methods (PCA, CoMem-DIPHW, CoMem, and Node2Vec) performed well, achieving average ARI scores above 0.9.

Additional results from intermediate parameter settings (average number of co-expressed genes per module: 20, 30, 40, 70, 80, 90; number of embedded modules: 20, 30, 40, 70, 80, 90), as measured by NMI, ACC, AMI, and *F*1, support the same observation. Across the 16 parameter settings under different modularity conditions, CoMem-DIPHW ranks first in all 16 settings by ACC and *F*1, 15 by ARI, and 10 each by NMI and AMI. DIPHW and CoMem also perform strongly. Either DIPHW or CoMem rank among the top three methods in at least 10 of 16 settings across all evaluation measures. Our hypergraph-based methods show significant advantages in weak modularity regimes, where data structures are harder to detect. Under comparatively strong modularity conditions, several methods (PCA, CoMem-DIPHW, CoMem, Node2Vec, DIPHW, and Leiden) perform well, with average NMI scores above 0.9. Notably, PCA slightly outperforms CoMem-DIPHW by ARI, NMI, and AMI under strong modularity, with differences in scores ranging from 0.01 to 0.02. These additional results are in Section S5, available as supplementary data at *Bioinformatics* online.

### 3.5 Results on the scMixology benchmark datasets

A major challenge in scRNA-seq analysis is the lack of reliable ground truth for cell-type annotation, which complicates the evaluation of clustering algorithms. In most scRNA-seq datasets, cell-type labels are assigned using a combination of clustering results, differential expression (DE) analysis, and canonical marker genes. First, cells are grouped into clusters based on transcriptomic similarity using clustering algorithms such as *K*-means. Next, DE analysis identifies genes that are differentially expressed across clusters. Finally, these clusters are annotated based on known canonical marker genes associated with specific cell types. Because this annotation process relies on clustering results, using these labels to evaluate clustering performance introduces circular logic and bias toward the method originally used for annotation. To address this issue, we use the mixture control benchmarking dataset scMixology (Tian *et al.* 2019), where ground truth is established based on cell line identity.

We evaluate the performance of our proposed method, CoMem-DIPHW, using two benchmarking datasets from scMixology (Tian *et al.* 2019). These datasets provide ground-truth labels for cell-type annotation based on human lung adenocarcinoma cell lines. As shown in Table 4, tsImpute achieved the highest scores across all five evaluation measures (ARI, NMI, AMI, ACC, and F1) on the 3-class dataset, with CoMem-DIPHW, Louvain, and Leiden tied for second. On the 5-class dataset, CoMem-DIPHW achieved the best performance across all evaluation measures, followed by tsImpute and graph-sc. This result is consistent with the pattern observed in our simulation analysis (see Section 3.4), where CoMem-DIPHW maintains strong performance under low modularity conditions.

**Table 4:** Performance of clustering methods on the scMixology benchmark datasets (Tian *et al.* 2019) across five evaluation measures (ARI, NMI, ACC, AMI, and *F*1).^a^

Traditional embedding and community detection methods								
Dataset/evaluation measures	GreedyModularity	Louvain	Infomap	Leiden	PCA	t-SNE	Node2Vec	UMAP
scMixology 3-class dataset								
ARI	0.584 ± 0.000	0.993 ± 0.000	0.233 ± 0.000	0.993 ± 0.000	0.990 ± 0.000	0.990 ± 0.000	0.991 ± 0.004	0.990 ± 0.000
NMI	0.719 ± 0.000	0.986 ± 0.000	0.384 ± 0.000	0.986 ± 0.000	0.980 ± 0.000	0.981 ± 0.000	0.983 ± 0.008	0.981 ± 0.000
ACC	0.693 ± 0.000	0.998 ± 0.000	0.337 ± 0.000	0.998 ± 0.000	0.996 ± 0.000	0.997 ± 0.000	0.997 ± 0.001	0.997 ± 0.000
AMI	0.719 ± 0.000	0.986 ± 0.000	0.234 ± 0.000	0.986 ± 0.000	0.980 ± 0.000	0.981 ± 0.000	0.983 ± 0.008	0.981 ± 0.000
*F*1	0.587 ± 0.000	0.998 ± 0.000	0.459 ± 0.000	0.998 ± 0.000	0.997 ± 0.000	0.997 ± 0.000	0.997 ± 0.001	0.997 ± 0.000
scMixology 5-class dataset								
ARI	0.665 ± 0.000	0.675 ± 0.000	0.300 ± 0.001	0.675 ± 0.000	0.987 ± 0.000	0.986 ± 0.000	0.945 ± 0.007	0.984 ± 0.000
NMI	0.785 ± 0.000	0.795 ± 0.000	0.436 ± 0.000	0.792 ± 0.000	0.975 ± 0.000	0.974 ± 0.000	0.915 ± 0.009	0.971 ± 0.000
ACC	0.733 ± 0.000	0.736 ± 0.000	0.415 ± 0.002	0.736 ± 0.000	0.994 ± 0.000	0.994 ± 0.000	0.962 ± 0.006	0.993 ± 0.000
AMI	0.785 ± 0.000	0.795 ± 0.000	0.274 ± 0.000	0.792 ± 0.000	0.975 ± 0.000	0.974 ± 0.000	0.915 ± 0.009	0.971 ± 0.000
*F*1	0.656 ± 0.000	0.658 ± 0.000	0.525 ± 0.003	0.657 ± 0.000	0.994 ± 0.000	0.994 ± 0.000	0.962 ± 0.006	0.993 ± 0.000

Advanced ScRNA-seq and hypergraph-based methods								
Dataset/evaluation measure	EDVW	graph-sc	tsImpute	CAKE	scASDC	DIPHW	CoMem	CoMem-DIPHW
scMixology 3-class dataset								
ARI	0.596 ± 0.045	0.702 ± 0.265	**0.997 ** ± ** 0.000**	0.832 ± 0.020	0.423 ± 0.434	0.990 ± 0.005	0.991 ± 0.002	0.993 ± 0.000
NMI	0.614 ± 0.027	0.765 ± 0.188	**0.993 ** ± ** 0.000**	0.861 ± 0.021	0.450 ± 0.440	0.980 ± 0.010	0.983 ± 0.003	0.986 ± 0.000
ACC	0.800 ± 0.056	0.861 ± 0.128	**0.999 ** ± ** 0.000**	0.996 ± 0.001	0.644 ± 0.296	0.997 ± 0.002	0.997 ± 0.001	0.998 ± 0.000
AMI	0.613 ± 0.027	0.765 ± 0.189	**0.993 ** ± ** 0.000**	0.861 ± 0.021	0.448 ± 0.442	0.980 ± 0.010	0.983 ± 0.003	0.986 ± 0.000
*F*1	0.799 ± 0.056	0.851 ± 0.138	**0.999 ** ± ** 0.000**	0.996 ± 0.001	0.548 ± 0.381	0.997 ± 0.002	0.997 ± 0.001	0.998 ± 0.000
scMixology 5-class dataset								
ARI	0.657 ± 0.022	0.988 ± 0.002	0.989 ± 0.000	0.733 ± 0.075	0.392 ± 0.360	0.944 ± 0.003	0.988 ± 0.001	**0.990 ** ± ** 0.002**
NMI	0.742 ± 0.020	0.978 ± 0.002	0.980 ± 0.000	0.844 ± 0.022	0.458 ± 0.416	0.909 ± 0.004	0.977 ± 0.003	**0.980 ** ± ** 0.003**
ACC	0.699 ± 0.016	0.995 ± 0.001	0.995 ± 0.000	0.857 ± 0.019	0.555 ± 0.216	0.963 ± 0.002	0.995 ± 0.001	**0.996 ** ± ** 0.001**
AMI	0.742 ± 0.020	0.978 ± 0.002	0.980 ± 0.000	0.843 ± 0.022	0.457 ± 0.417	0.909 ± 0.004	0.977 ± 0.003	**0.980 ** ± ** 0.003**
*F*1	0.711 ± 0.014	0.995 ± 0.001	0.995 ± 0.000	0.858 ± 0.018	0.344 ± 0.225	0.963 ± 0.002	0.995 ± 0.001	**0.996 ** ± ** 0.001**

a The scMixology benchmark datasets include a 3-class and a 5-class dataset. Results for each are shown in the top and bottom sections, respectively. Best values are in bold, and second best are underlined. Results are reported as mean ± standard deviation over five independent runs. On the 3-class dataset, tsImpute achieved the highest scores across all evaluation measures, with CoMem-DIPHW, Louvain, and Leiden tied for second. On the more challenging 5-class dataset, CoMem-DIPHW achieved the best performance across all evaluation measures, outperforming tsImpute, which ranked second.

### 3.6 Results on the human pancreas dataset

For datasets without ground-truth annotations (specifically, human pancreas, mouse pancreas, human brain, and mouse brain), we qualitatively evaluate clustering performance using DE analysis. We present detailed DE analysis results for the human pancreas dataset using CoMem-DIPHW, PCA, graph-sc, tsImpute, CAKE, and scASDC in this section and provide results for the human brain, mouse brain, and mouse pancreas in Sections S6 and S7, available as supplementary data at *Bioinformatics* online.

**Figure 7:**
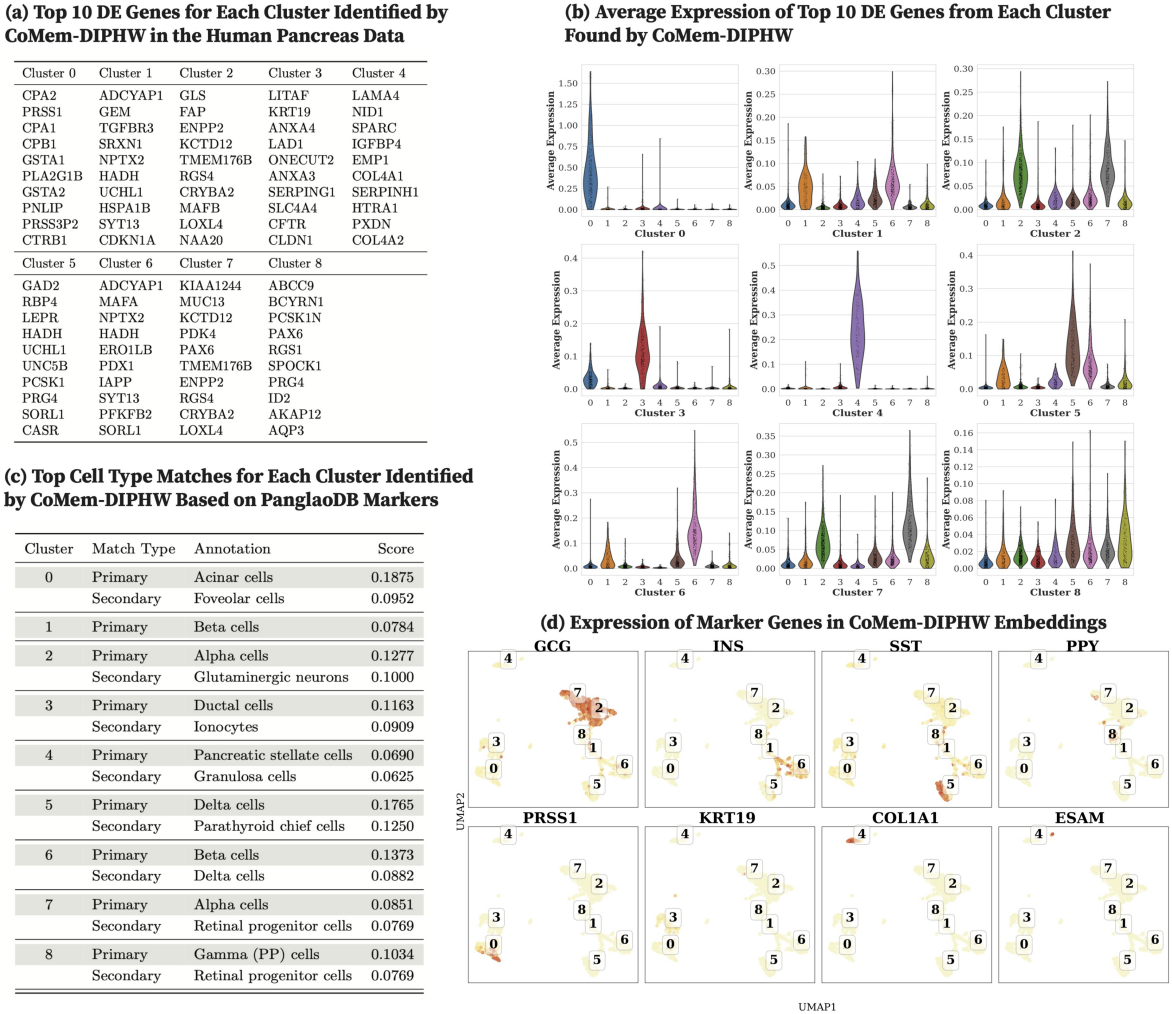
Clustering performance of CoMem-DIPHW on the human pancreas dataset and cell-type annotation using differentially expressed genes (DEGs) and canonical markers. (a) Top 10 DEGs per cluster identified by CoMem-DIPHW. For example, Cluster 0 (acinar cells) includes genes associated with digestive enzyme production, a primary function of pancreatic acinar cells, including CPA1, CPA2 (Tamura *et al*. 2018), PLA2G1B (Hui 2019), CTRB1 (Pinho *et al*. 2011), and PRSS1 (Masson *et al*. 2013). Clusters 2 and 7 (alpha cells) include markers GLS, CRYBA2, FAP, LOXL4, MAFB, and RGS4 (Cluster 2), and LOXL4, PAX6, RGS4, and CRYBA2 (Cluster 7). Cluster 3 (ductal cells) includes SLC4A4, ANXA4, KRT19, CFTR, CLDN1, and ONECUT2. Cluster 4, likely mesenchymal or endothelial cells, aligns with nine of the top 10 DEGs found by CoMem-DIPHW, including COL4A2, COL4A1, SPARC, EMP1, IGFBP4, HTRA1, PXDN, LAMA4, and NID1. Cluster 5 (delta cells) includes GAD2, PCSK1, LEPR, and CASR, while Cluster 6 (beta cells) includes MAFA, ERO1LB, PDX1, IAPP, SYT13, HADH, and ADCYAP1. Cell-type assignments are based on canonical marker expression patterns shown in (d). (b) Across-cluster average expression of cluster-specific DEGs. Violin plots show the distribution of average expression levels of these DEGs across all clusters. Strong clustering performance is indicated by high within-cluster expression of DEGs and low expression in other clusters. (c) Cell-type annotation using the PanglaoDB marker database. Cell types are determined by the overlap between each cluster’s DEGs and PanglaoDB cell type-specific markers, with match scores computed based on the proportion of matched markers. (d) Expression of human pancreatic canonical markers in CoMem-DIPHW embeddings. UMAP visualizations show CoMem-DIPHW cell embeddings, with cluster IDs assigned by *K*-means clustering. Each subplot displays the expression of a canonical marker gene for a specific human pancreatic cell type. The cell-type-specific markers considered include GCG (alpha cells), INS (beta cells), SST (delta cells), PPY (PP cells), PRSS1 (acinar cells), KRT19 (ductal cells), COL1A1 (mesenchymal cells), and ESAM (endothelial cells). Color intensity represents gene expression levels, with red hues indicating higher expression. Clusters showing high expression of cell-type-specific canonical markers are assigned to the corresponding cell types. Overall, clustering quality is demonstrated by (b) high within-cluster DEG expression, (c) consistency between PanglaoDB-based annotation and clustering, and (d) distinct separation of cell types.

**Figure 8:**
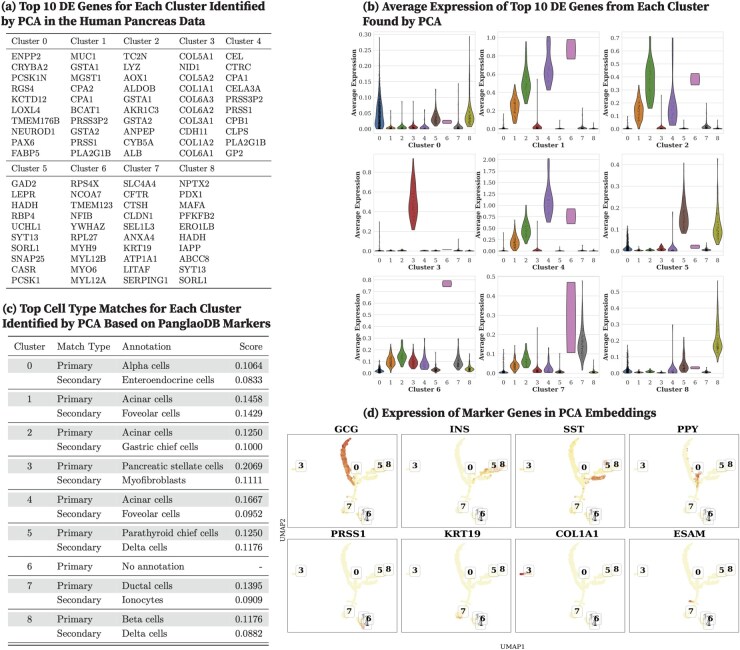
Clustering performance of PCA on the human pancreas dataset and cell-type annotation using differentially expressed genes (DEGs) and canonical markers. (a) Top 10 DEGs per cluster identified by PCA. (b) Average expression of cluster-specific DEGs across clusters. Strong clustering performance is indicated by high expression of cluster-specific DEGs within their respective clusters and low expression in other clusters. (c) Cell-type annotation using the PanglaoDB marker database. (d) Expression of human pancreatic canonical markers in PCA embeddings. UMAP visualizations show PCA cell embeddings, with cluster IDs assigned by *K*-means clustering. Overall, the clustering of cell types is limited compared to CoMem-DIPHW clustering, as shown by the overlapping beta cells, delta cells, and PP cells in (d) and the absence of distinct high expression patterns of the DEGs in (b).

**Figure 9:**
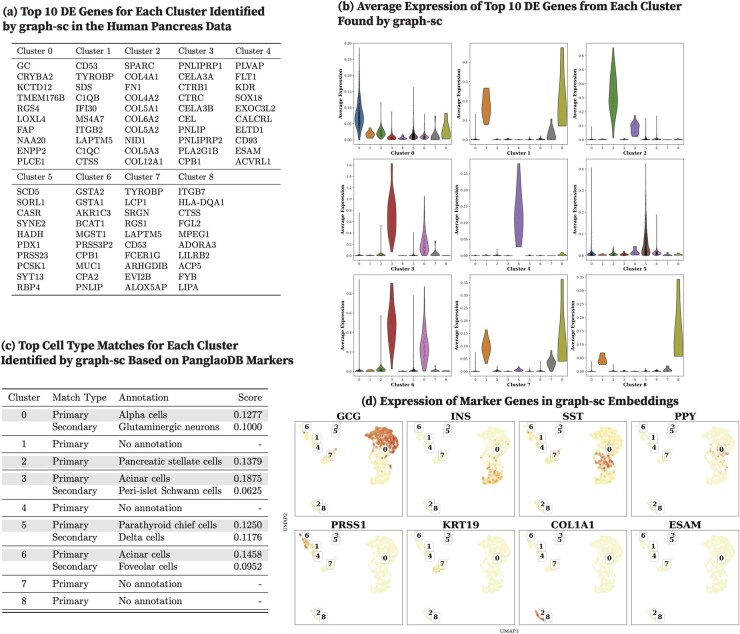
Clustering performance of graph-sc on the human pancreas dataset and cell-type annotation using differentially expressed genes (DEGs) and canonical markers. (a) Top 10 DEGs per cluster identified by graph-sc. (b) Across expression of cluster-specific DEGs across clusters. Strong clustering performance is indicated by high expression of cluster-specific DEGs within their respective clusters and low expression in other clusters. Graph-sc clusters generally show moderate specificity with substantial cross-cluster overlap. (c) Cell-type annotation using the PanglaoDB marker database. Four clusters (1, 4, 7, and 8) yield no annotation, indicating that their DEGs did not match known cell-type markers. (d) Expression of human pancreatic canonical markers in graph-sc embeddings. UMAP visualizations show graph-sc cell embeddings, with cluster IDs assigned by *K*-means clustering. Overall, cell cluster separation is limited.

**Figure 10:**
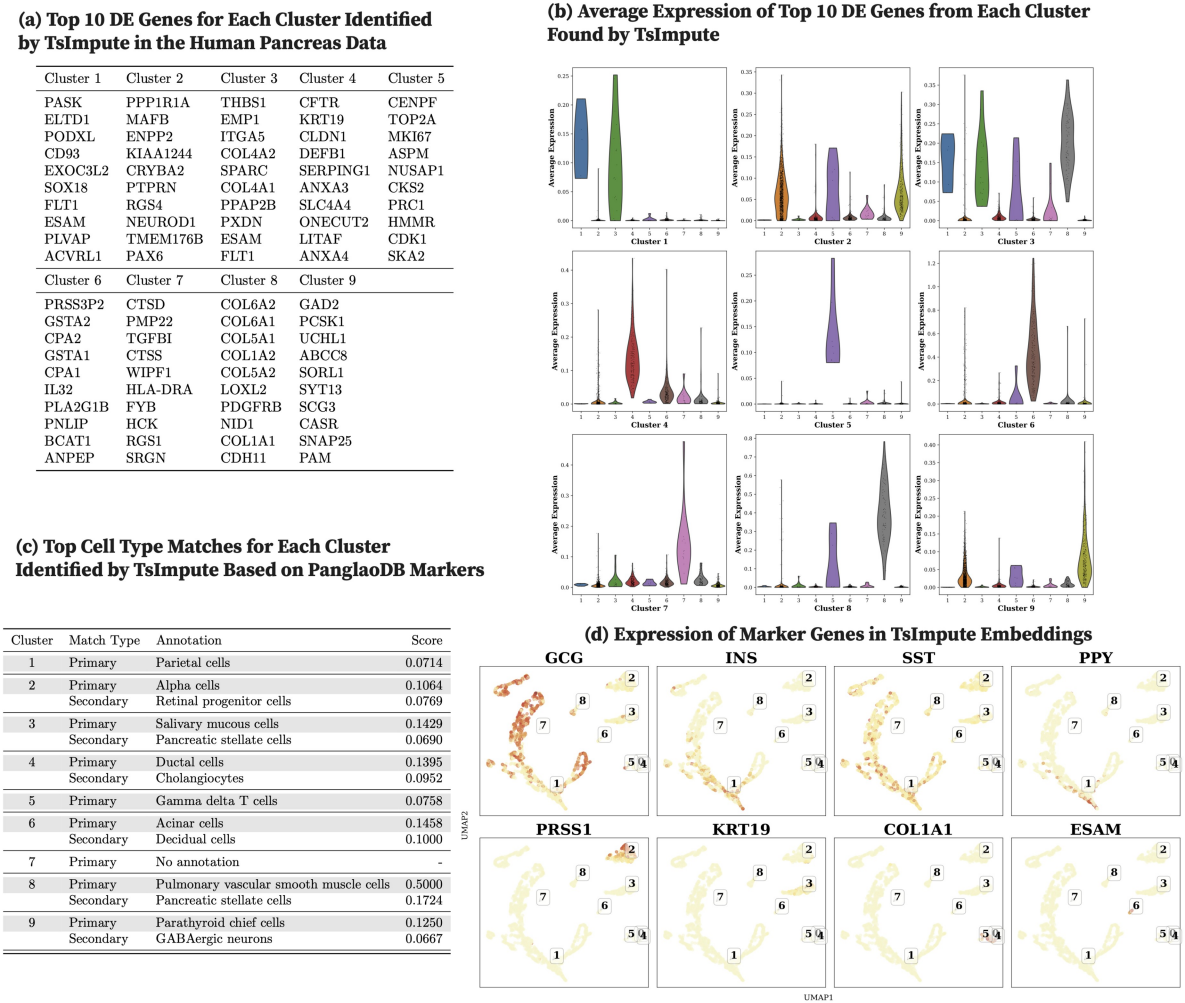
Clustering performance of tsImpute on the human pancreas dataset and cell-type annotation using differentially expressed genes (DEGs) and canonical markers. (a) Top 10 DEGs per cluster identified by tsImpute combined with PCA embeddings and *K*-means. (b) Average expression of cluster-specific DEGs across clusters. Strong clustering performance is indicated by high expression of cluster-specific DEGs within their respective clusters and low expression in other clusters. tsImpute DEGs show high specificity in Clusters 2, 4, 5, 6, 7, 8, and 9. (c) Cell-type annotation using the PanglaoDB marker database. Several annotations are biologically implausible for pancreas tissue, including parietal cells (Cluster 1), salivary mucous cells (Cluster 3), gamma delta T cells (Cluster 5), and pulmonary vascular smooth muscle cells (Cluster 8). These unexpected annotations suggest that the imputation process may introduce artifacts that confound cell-type identification. (d) Expression of human pancreatic canonical markers in tsImpute combined with PCA embeddings. UMAP visualizations show tsImpute plus PCA cell embeddings, with cluster IDs assigned by *K*-means clustering. tsImpute improves cluster separation compared to PCA without imputation.

**Figure 11:**
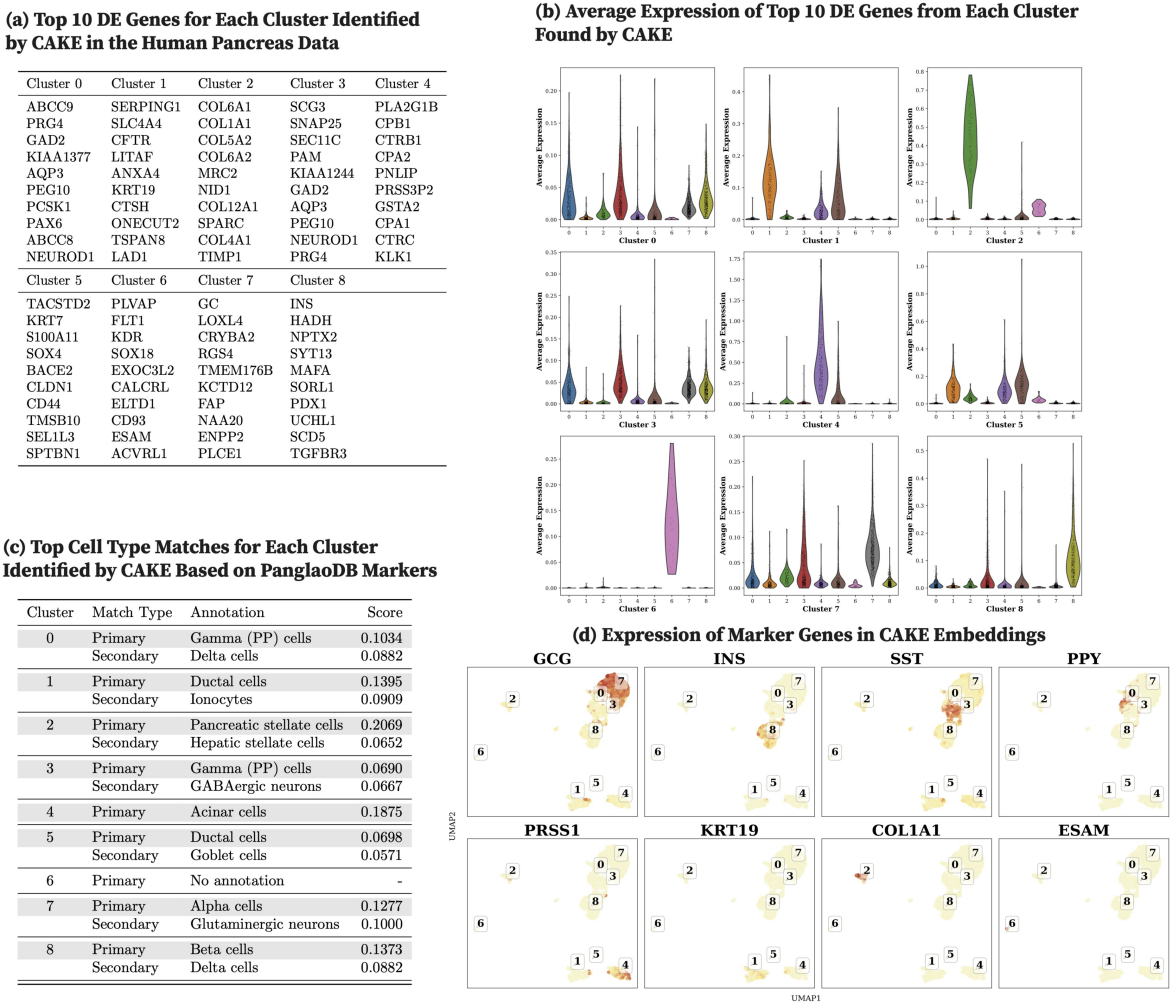
Clustering performance of CAKE on the human pancreas dataset and cell-type annotation using differentially expressed genes (DEGs) and canonical markers. (a) Top 10 DEGs per cluster identified by CAKE. (b) Average expression of cluster-specific DEGs across clusters. Strong clustering performance is indicated by high expression of cluster-specific DEGs within their respective clusters and low expression in other clusters. CAKE DEGs show good cluster specificity. (c) Cell-type annotation using the PanglaoDB marker database. Most CAKE clusters correspond to biologically relevant cell types: gamma/PP cells (Clusters 0 and 3), ductal cells (Clusters 1 and 5), pancreatic stellate cells (Cluster 2), acinar cells (Cluster 4), alpha cells (Cluster 7), and beta cells (Cluster 8). (d) Expression of human pancreatic canonical markers in CAKE embeddings. UMAP visualizations show CAKE cell embeddings, with cluster IDs assigned by *K*-means clustering. Marker genes show distinct concentration in the well-separated CAKE embeddings: GCG in Cluster 7, INS in Cluster 8, SST in Clusters 0 and 3, PRSS1 in Cluster 4, and KRT19 in Cluster 1.

**Figure 12:**
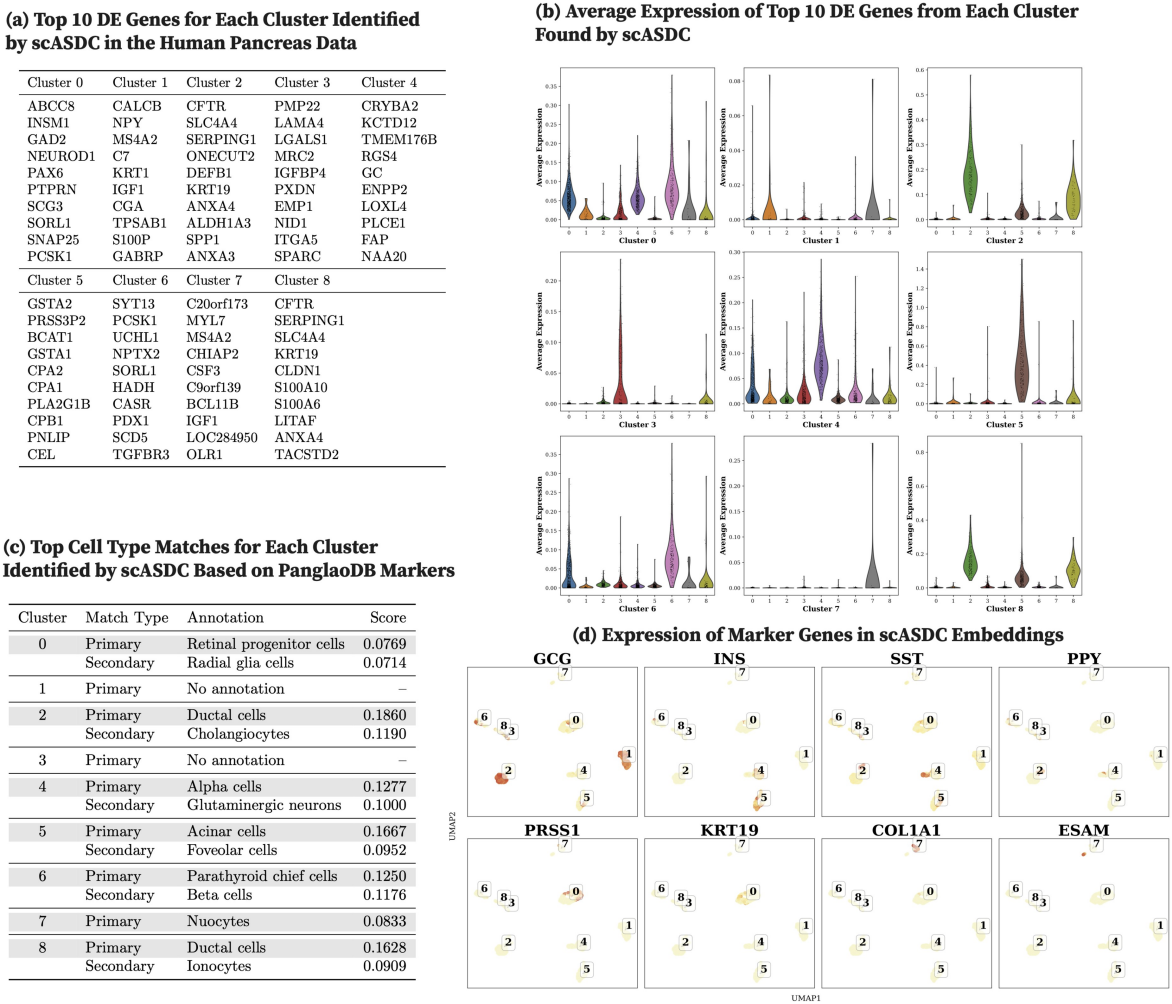
Clustering performance of scASDC on the human pancreas dataset and cell-type annotation using differentially expressed genes (DEGs) and canonical markers. (a) Top 10 DEGs per cluster identified by scASDC. (b) Average expression of cluster-specific DEGs across clusters. Strong clustering performance is indicated by high expression of cluster-specific DEGs within their respective clusters and low expression in other clusters. scASDC DEGs show good cluster specificity. (c) Cell-type annotation using the PanglaoDB marker database. While most clusters are matched to relevant pancreatic cell types, some are annotated with unexpected cell types, including retinal progenitor cells (Cluster 0) and nuocytes (Cluster 7). Clusters 1 and 3 received no annotation. (d) Expression of human pancreatic canonical markers in scASDC embeddings. scASDC embeddings show the best internal cohesion and separability. The marker genes show distinct concentration in the well-separated scASDC embeddings. However, the cell-type annotations suggest that the embeddings may not necessarily correspond to biologically meaningful clusters.

We report the top 10 DEGs from each human pancreas cell cluster identified by CoMem-DIPHW (Fig. 7a), PCA (Fig. 8a), graph-sc (Fig. 9a), tsImpute (Fig. 10a), CAKE (Fig. 11a), and scASDC (Fig. 12a). To evaluate cluster purity, we examine the expression of these DEGs across clusters (Figs 7b–12b). An effective clustering method should yield DEGs that uniquely characterize each cluster while showing minimal expression in other clusters.

Violin plots show that the DEGs identified by CoMem-DIPHW exhibit more cluster-specific expression patterns than those derived from PCA, which tend to show higher expression in multiple clusters. For example, the average expression of the DEGs from PCA Cluster 1 is higher in Clusters 2, 5, and 6. Similarly, the DEGs from Cluster 7 show higher average expression in Cluster 6. Among the comparison methods, CAKE and scASDC produced highly distinctive DEGs, with scASDC yielding clusters that showed the strongest intra-cluster cohesion and inter-cluster separability. Graph-sc and PCA show less cluster-specific profiles, with DEGs frequently expressed across multiple clusters.

To assess the biological relevance of the clusters, we cross-reference the cluster-specific DEGs (see Fig. 7a) with markers from the PanglaoDB cell-type marker database (version 27 March 2020) (Franzén *et al.* 2019). Cell-type assignment is based on the overlap between the DEGs of each cluster and the cell type-specific markers in the PanglaoDB database, with match scores calculated as the proportion of each cell type’s PanglaoDB marker genes that overlap with the cluster’s DEGs (Fig. 7c). CoMem-DIPHW (Fig. 7c) successfully annotates all nine clusters with biologically relevant pancreatic cell types. CAKE produces similarly meaningful annotations, while graph-sc has several clusters with no annotation or biologically implausible labels.

To further validate the clustering results, we examine the expression of canonical pancreatic marker genes (Muraro *et al.* 2016), including GCG (alpha cells), INS (beta cells), SST (delta cells), PPY (PP cells), PRSS1 (acinar cells), KRT19 (ductal cells), COL1A1 (mesenchymal cells), and ESAM (endothelial cells) in the embeddings (Figs 7d–12d). Each marker is distinctly expressed in the embedding generated by CoMem-DIPHW, as shown in Fig. 7d, confirming that CoMem-DIPHW embeddings effectively distinguish different cell types. CAKE and scASDC also produce well-separated embeddings. In contrast, PCA embeddings show poor separation. The results from CoMem-DIPHW align well with the cell-type annotation by the PanglaoDB database. The only discrepancy is in Cluster 4. Matching the DEGs with PanglaoDB cell-type markers suggests pancreatic stellate cells (PSCs) or granulosa cells, while the COL1A1 and ESAM expression plots indicate mesenchymal or endothelial cells. However, this apparent discrepancy can be explained by the fact that PSCs can differentiate into cells with endothelial-like properties (Cheng *et al.* 2021), and granulosa cells can exhibit mesenchymal-like behaviors (Jozkowiak *et al.* 2020).

In contrast, PCA clustering results (Fig. 8) showed limited cluster separation. While some PCA clusters could be annotated consistently using both PanglaoDB markers and canonical markers, significant overlap remained among clusters. For example, PCA Cluster 8, identified as beta cells by both the PanglaoDB markers and the canonical marker INS, is not well separated from Cluster 5 (potentially delta cells, marked by SST expression). Similarly, Cluster 7, identified as ductal cells by both the PanglaoDB markers and the canonical marker KRT19, is closely embedded with endothelial cells (ESAM). These results highlight CoMem-DIPHW’s improved ability to distinguish pancreatic cell types compared to PCA.

In addition to the human pancreas results presented here, we also analyse mouse pancreas, human brain, and mouse brain datasets. For these datasets, we include visualizations in Figs S12b–S29b, available as supplementary data at *Bioinformatics* online, that show the average expression of the top DEGs across clusters identified by different methods. These results show our proposed algorithm’s ability in effectively distinguishing cell clusters by their expression profiles. The supplementary material (Figs S12a–S29a, available as supplementary data at *Bioinformatics* online), also contain tables listing the top DEGs for clusters identified by CoMem-DIPHW, PCA, graph-sc, tsImpute, CAKE, and scASDC in each dataset for reference.

To complement the qualitative biological validation presented above, we further assess clustering performance using quantitative cluster quality measures, including the Silhouette score, Calinski–Harabasz index, Davies–Bouldin index, and coefficient of variation. These evaluations are conducted in two settings: (i) directly in the learned embedding space and (ii) by evaluating the same cluster assignments on the preprocessed expression data used as input to the embedding algorithms. We refer the reader to Tables S2 and S3, available as supplementary data at *Bioinformatics* online. Evaluation in the embedding space captures the geometric separability and cohesion of clusters, whereas evaluation on the preprocessed data assesses whether the resulting cluster assignments capture meaningful structure in the input gene expression data. As demonstrated by DE and cell-type annotation analyses in the human pancreas data set, strong embedding-space cohesion and separation alone do not necessarily guarantee biological interpretability.

## 4 Conclusion

In this study, we develop and analyse two hypergraph-based clustering algorithms, DIPHW and CoMem-DIPHW, to improve cell-clustering performance on scRNA-seq data. First, the DIP Hypergraph Walk (DIPHW) extends the EDVW hypergraph random walk (Chitra and Raphael 2019) and consistently outperforms it in our experiments. The key idea of DIPHW is its symmetrized treatment of importance, considering both the relative importance of edges to nodes and nodes to edges, or the importance of genes relative to cells and cells to genes, along with a preference exponent for faster clustering. Second, the CoMem and CoMem-DIPHW clustering methods incorporate a memory mechanism that considers both the abundance counts data (conceptualized as hypergraphs) and the two co-expression networks to integrate local and global information when computing embeddings for clustering. Third, we identify and demonstrate through experiments (see Fig. 3) the issue of inflated signals in co-expression networks caused by the sparsity of scRNA-seq data, which further motivates the use of hypergraph representations. Fourth, we compare our proposed methods with 13 cell-clustering methods from various categories, including community detection, embedding-based methods, and recent deep learning methods used in clustering scRNA-seq data. Our evaluation covers a broad range of modularity conditions in the data, where CoMem-DIPHW and DIPHW consistently show superior performance, particularly in weak modularity regimes. When clusters are harder to detect, other methods experience a much more pronounced decline in performance compared to CoMem-DIPHW. Fifth, we test our proposed methods on four real scRNA-seq datasets and two benchmark datasets. In these experiments, CoMem-DIPHW demonstrates superior performance in differentiating and classifying distinct cell types compared to other methods. Sixth, we design a versatile simulation algorithm for scRNA-seq data that is highly customizable through many user-specified parameters. This algorithm models a wide range of expression scenarios and modular structures. Notably, it incorporates intermodular co-expressions, which mimic cell-type cross-talk observed in real biological systems.

### 4.1 Future work

We plan to optimize the implementation of CoMem-DIPHW to reduce its memory complexity, enabling it to scale to larger datasets. We will also explore alternative methods for incorporating the memory mechanism into the hypergraph random walk. Currently, CoMem uses a simple product approach; moving beyond this could reveal more effective clustering strategies. Although our current evaluation focuses on the biological relevance of clusters through cell-type annotation using PanglaoDB markers, future application-focused studies could include Gene Ontology enrichment analysis to characterize the functions of identified clusters.

With the increasing availability of data and studies in spatial transcriptomics (Longo *et al.* 2021, Rao *et al.* 2021, Kleshchevnikov *et al.* 2022, Li *et al.* 2024), extending the CoMem-DIPHW formulation for spatial transcriptomics analysis is a promising direction. While the current CoMem-DIPHW formulation focuses on cell clustering based on expression profile similarity, it could be extended to incorporate spatial proximity by replacing the cell expression similarity matrix $G_{V}$ with functions that characterize joint cell similarity based on both expression profiles and spatial information.

## Supplementary Material

btag148_Supplementary_Data

## Data Availability

The implementation of CoMem-DIPHW is freely available on GitHub at https://github.com/wanhe13/CoMem-DIPHW and archived on Zenodo (https://doi.org/10.5281/zenodo.18927437). The baseline methods compared in this study are available via their original authors’ repositories: graph-sc (https://github.com/ciortanmadalina/graph-sc), tsImpute (https://github.com/ZhengWeihuaYNU/tsImpute), CAKE (https://github.com/CSUBioGroup/CAKE), and scASDC (https://github.com/wenwenmin/scASDC). The real scRNA-seq datasets used in this study are publicly available and can be accessed via their respective publications: human brain (Camp *et al.* 2015), human pancreas (Muraro *et al.* 2016), mouse brain (Zeisel *et al.* 2015), mouse pancreas (Baron *et al.* 2016), and the scMixology benchmarks (Tian *et al.* 2019).

## References

[btag148-B1] Andrews TS , Kiselev VY, McCarthy D et al Tutorial: guidelines for the computational analysis of single-cell RNA sequencing data. Nat Protoc 2021;16:1–9. 33288955 10.1038/s41596-020-00409-w

[btag148-B2] Armingol E , Officer A, Harismendy O et al Deciphering cell–cell interactions and communication from gene expression. Nat Rev Genet 2021;22:71–88. 33168968 10.1038/s41576-020-00292-x PMC7649713

[btag148-B3] Ashtiani M , Salehzadeh-Yazdi A, Razaghi-Moghadam Z et al A systematic survey of centrality measures for protein–protein interaction networks. BMC Syst Biol 2018;12:80. 30064421 10.1186/s12918-018-0598-2 PMC6069823

[btag148-B4] Barber MJ. Modularity and community detection in bipartite networks. Phys Rev E Stat Nonlin Soft Matter Phys 2007;76:066102. 18233893 10.1103/PhysRevE.76.066102

[btag148-B5] Baron M , Veres A, Wolock SL et al A single-cell transcriptomic map of the human and mouse pancreas reveals inter- and intra-cell population structure. Cell Syst 2016;3:346–60.e4. 27667365 10.1016/j.cels.2016.08.011 PMC5228327

[btag148-B6] Battiston F , Cencetti G, Iacopini I et al Networks beyond pairwise interactions: structure and dynamics. Phys Rep 2020;874:1–92.

[btag148-B7] Battiston F , Amico E, Barrat A et al The physics of higher-order interactions in complex systems. Nat Phys 2021;17:1093–8.

[btag148-B8] Bayik D , Lathia JD. Cancer stem cell–immune cell crosstalk in tumour progression. Nat Rev Cancer 2021;21:526–36. 34103704 10.1038/s41568-021-00366-w PMC8740903

[btag148-B9] Bianconi G. Higher-Order Networks. Cambridge, UK: Cambridge University Press, 2021.

[btag148-B10] Blondel VD , Guillaume J-L, Lambiotte R et al Fast unfolding of communities in large networks. J Stat Mech 2008;2008:P10008.

[btag148-B11] Butler A , Hoffman P, Smibert P et al Integrating single-cell transcriptomic data across different conditions, technologies, and species. Nat Biotechnol 2018;36:411–20. 29608179 10.1038/nbt.4096 PMC6700744

[btag148-B12] Camp JG , Badsha F, Florio M et al Human cerebral organoids recapitulate gene expression programs of fetal neocortex development. Proc Natl Acad Sci USA 2015;112:15672–7. 26644564 10.1073/pnas.1520760112 PMC4697386

[btag148-B13] Cheng L , Xie M, Qiao W et al Generation and characterization of cardiac valve endothelial-like cells from human pluripotent stem cells. Commun Biol 2021;4:1039. 34489520 10.1038/s42003-021-02571-7 PMC8421482

[btag148-B14] Chinazzi M , Davis JT, Ajelli M et al The effect of travel restrictions on the spread of the 2019 novel coronavirus (covid-19) outbreak. Science 2020;368:395–400. 32144116 10.1126/science.aba9757 PMC7164386

[btag148-B15] Chitra U , Raphael BJ. Random walks on hypergraphs with edge-dependent vertex weights. In: Chaudhuri K, Salakhutdinov R (eds.), Proceedings of the 36th International Conference on Machine Learning, ICML 2019, 9–15 June 2019, Long Beach, California, USA, Volume 97 of Proceedings of Machine Learning Research. Long Beach, CA, USA: PMLR. 2019, 1172–81.

[btag148-B16] Chung W , Eum HH, Lee H-O et al Single-cell RNA-seq enables comprehensive tumour and immune cell profiling in primary breast cancer. Nat Commun 2017;8:15081. 28474673 10.1038/ncomms15081 PMC5424158

[btag148-B17] Ciortan M , Defrance M. GNN-based embedding for clustering scRNA-seq data. Bioinformatics 2022;38:1037–44. 34850828 10.1093/bioinformatics/btab787

[btag148-B18] Coleman DJL , Keane P, Luque-Martin R et al Gene regulatory network analysis predicts cooperating transcription factor regulons required for FLT3-ITD+ AML growth. Cell Rep 2023;42:113568. 38104314 10.1016/j.celrep.2023.113568 PMC10874628

[btag148-B19] Csardi G , Nepusz T. The igraph software package for complex network research. InterJ Complex Syst 2006;1695.

[btag148-B20] Davis JT , Chinazzi M, Perra N et al Cryptic transmission of SARS-CoV-2 and the first covid-19 wave. Nature 2021;600:127–32. 34695837 10.1038/s41586-021-04130-w PMC8636257

[btag148-B21] Ermann J , Rao DA, Teslovich NC et al Immune cell profiling to guide therapeutic decisions in rheumatic diseases. Nat Rev Rheumatol 2015;11:541–51. 26034835 10.1038/nrrheum.2015.71 PMC4898649

[btag148-B22] Franzén O , Gan L-M, Björkegren JL. PanglaoDB: a web server for exploration of mouse and human single-cell RNA sequencing data. Database 2019;2019:baz046. 30951143 10.1093/database/baz046 PMC6450036

[btag148-B23] Gaudelet T , Malod-Dognin N, Pržulj N. Higher-order molecular organization as a source of biological function. Bioinformatics 2018;34:i944–53. 30423061 10.1093/bioinformatics/bty570 PMC6129285

[btag148-B24] Grover A , Leskovec J. node2vec: scalable feature learning for networks. In: *Proceedings of the 22nd ACM SIGKDD International Conference on Knowledge Discovery and Data Mining*. New York, NY, USA: ACM, 2016, 855–64. 10.1145/2939672.2939754 PMC5108654 27853626

[btag148-B25] He W , Bolnick DI, Scarpino SV et al CoMem-DIPHW: coexpression and memory-integrated dual-importance preference hypergraph walk. 2026. 10.5281/zenodo.18927437.

[btag148-B26] Horvát E-A , Zweig KA. One-mode projection of multiplex bipartite graphs. In: 2012 IEEE/ACM International Conference on Advances in Social Networks Analysis and Mining. Piscataway, NJ, USA: IEEE, 2012, 599–606.

[btag148-B27] Huang M , Wang J, Torre E et al SAVER: gene expression recovery for single-cell RNA sequencing. Nat Methods 2018;15:539–42. 29941873 10.1038/s41592-018-0033-z PMC6030502

[btag148-B28] Hui DY. Group 1B phospholipase A2 in metabolic and inflammatory disease modulation. Biochim Biophys Acta Mol Cell Biol Lipids 2019;1864:784–8. 30003964 10.1016/j.bbalip.2018.07.001 PMC6328335

[btag148-B29] Hwang B , Lee JH, Bang D. Single-cell RNA sequencing technologies and bioinformatics pipelines. Exp Mol Med 2018;50:1–14. 10.1038/s12276-018-0071-8 PMC6082860 30089861

[btag148-B30] Jiang R , Sun T, Song D et al Statistics or biology: the zero-inflation controversy about scRNA-seq data. Genome Biol 2022;23:31. 35063006 10.1186/s13059-022-02601-5 PMC8783472

[btag148-B31] Jozkowiak M , Hutchings G, Jankowski M et al The stemness of human ovarian granulosa cells and the role of resveratrol in the differentiation of MSCs—a review based on cellular and molecular knowledge. Cells 2020;9:1418. 32517362 10.3390/cells9061418 PMC7349183

[btag148-B32] Karaayvaz M , Cristea S, Gillespie SM et al Unravelling subclonal heterogeneity and aggressive disease states in TNBC through single-cell RNA-seq. Nat Commun 2018;9:3588. 30181541 10.1038/s41467-018-06052-0 PMC6123496

[btag148-B33] Kim TH , Zhou X, Chen M. Demystifying “drop-outs” in single-cell UMI data. Genome Biol 2020;21:196. 32762710 10.1186/s13059-020-02096-y PMC7412673

[btag148-B34] Kiselev VY , Kirschner K, Schaub MT et al SC3: consensus clustering of single-cell RNA-seq data. Nat Methods 2017;14:483–6. 28346451 10.1038/nmeth.4236 PMC5410170

[btag148-B35] Kleshchevnikov V , Shmatko A, Dann E et al Cell2location maps fine-grained cell types in spatial transcriptomics. Nat Biotechnol 2022;40:661–71. 35027729 10.1038/s41587-021-01139-4

[btag148-B36] Kovács IA , Luck K, Spirohn K et al Network-based prediction of protein interactions. Nat Commun 2019;10:1240. 30886144 10.1038/s41467-019-09177-y PMC6423278

[btag148-B37] Kraemer MUG , Yang C-H, Gutierrez B, Open COVID-19 Data Working Group et al The effect of human mobility and control measures on the covid-19 epidemic in China. Science 2020;368:493–7. 32213647 10.1126/science.abb4218 PMC7146642

[btag148-B38] Kuzmin E , VanderSluis B, Wang W et al Systematic analysis of complex genetic interactions. Science 2018;360:eaao1729. 29674565 10.1126/science.aao1729 PMC6215713

[btag148-B39] Landhuis E. Single-cell approaches to immune profiling. Nature 2018;557:595–7. 29789748 10.1038/d41586-018-05214-w

[btag148-B40] Langfelder P , Horvath S. WGCNA: An R package for weighted correlation network analysis. BMC Bioinformatics 2008; 9:559. 19114008 10.1186/1471-2105-9-559 PMC2631488

[btag148-B41] Laviron M , Petit M, Weber-Delacroix E et al Tumor-associated macrophage heterogeneity is driven by tissue territories in breast cancer. Cell Rep 2022;39:110865. 35613577 10.1016/j.celrep.2022.110865

[btag148-B42] Lehuen A , Diana J, Zaccone P et al Immune cell crosstalk in type 1 diabetes. Nat Rev Immunol 2010;10:501–13. 20577267 10.1038/nri2787

[btag148-B43] Lei C , Ruan J. A novel link prediction algorithm for reconstructing protein–protein interaction networks by topological similarity. Bioinformatics 2013;29:355–64. 23235927 10.1093/bioinformatics/bts688 PMC3562060

[btag148-B44] Li X , Zhu F, Min W. SpaDiT: diffusion transformer for spatial gene expression prediction using scRNA-seq. Brief Bioinform 2024;25:bbae571. 39508444 10.1093/bib/bbae571 PMC11541600

[btag148-B45] Liu J , Zeng W, Kan S et al CAKE: a flexible self-supervised framework for enhancing cell visualization, clustering and rare cell identification. Brief Bioinform 2023;25:bbad475. 38145950 10.1093/bib/bbad475 PMC10749894

[btag148-B46] Liu X , Song C, Liu S et al Multi-way relation-enhanced hypergraph representation learning for anti-cancer drug synergy prediction. Bioinformatics 2022;38:4782–9. 36000898 10.1093/bioinformatics/btac579

[btag148-B47] Longo SK , Guo MG, Ji AL et al Integrating single-cell and spatial transcriptomics to elucidate intercellular tissue dynamics. Nat Rev Genet 2021;22:627–44. 34145435 10.1038/s41576-021-00370-8 PMC9888017

[btag148-B48] Lyons YA , Wu SY, Overwijk WW et al Immune cell profiling in cancer: molecular approaches to cell-specific identification. NPJ Precis Oncol 2017;1:26. 29872708 10.1038/s41698-017-0031-0 PMC5871917

[btag148-B49] Ma Y , Ma Y. Hypergraph-based logistic matrix factorization for metabolite–disease interaction prediction. Bioinformatics 2022;38:435–43. 34499104 10.1093/bioinformatics/btab652

[btag148-B50] Macosko EZ , Basu A, Satija R et al Highly parallel genome-wide expression profiling of individual cells using nanoliter droplets. Cell 2015;161:1202–14. 26000488 10.1016/j.cell.2015.05.002 PMC4481139

[btag148-B51] Marioni JC , Arendt D. How single-cell genomics is changing evolutionary and developmental biology. Annu Rev Cell Dev Biol 2017;33:537–53. 28813177 10.1146/annurev-cellbio-100616-060818

[btag148-B52] Masson E , Chen J-M, Audrézet M-P et al A conservative assessment of the major genetic causes of idiopathic chronic pancreatitis: data from a comprehensive analysis of PRSS1, SPINK1, CTRC and CFTR genes in 253 young French patients. PLoS One 2013;8:e73522. 23951356 10.1371/journal.pone.0073522 PMC3738529

[btag148-B53] McInnes L , Healy J, Saul N et al UMAP: Uniform manifold approximation and projection. J Open Source Softw 2018;3:861.

[btag148-B54] Mikolov T, Chen K, Corrado G et al. Efficient estimation of word representations in vector space. In: *International Conference on Learning Representations, Workshop Track Proceedings*. Scottsdale, AZ, USA, 2013.

[btag148-B55] Mikolov T , Sutskever I, Chen K et al Distributed representations of words and phrases and their compositionality. Adv Neural Inf Process Syst 2013;26.

[btag148-B56] Min W , Wang Z, Zhu F et al scASDC: attention enhanced structural deep clustering for single-cell RNA-seq data. In: 2024 IEEE International Conference on Bioinformatics and Biomedicine (BIBM). Piscataway, NJ, USA: IEEE, 2024, 1092–7.

[btag148-B57] Murakami Y , Tripathi LP, Prathipati P et al Network analysis and in silico prediction of protein–protein interactions with applications in drug discovery. Curr Opin Struct Biol 2017;44:134–42. 28364585 10.1016/j.sbi.2017.02.005

[btag148-B58] Muraro MJ , Dharmadhikari G, Grün D et al A single-cell transcriptome atlas of the human pancreas. Cell Syst 2016;3:385–94.e3. 27693023 10.1016/j.cels.2016.09.002 PMC5092539

[btag148-B59] Muzio G , O’Bray L, Borgwardt K. Biological network analysis with deep learning. Brief Bioinform 2021;22:1515–30. 33169146 10.1093/bib/bbaa257 PMC7986589

[btag148-B60] Newman ME. Fast algorithm for detecting community structure in networks. Phys Rev E Stat Nonlin Soft Matter Phys 2004;69:066133. 15244693 10.1103/PhysRevE.69.066133

[btag148-B61] Pearson K. On lines and planes of closest fit to systems of points in space. Lond Edinb Dubl Phil Mag J Sci 1901;2:559–72.

[btag148-B62] Picelli S , Faridani OR, Björklund ÅK et al Full-length RNA-seq from single cells using Smart-seq2. Nat Protoc 2014;9:171–81. 24385147 10.1038/nprot.2014.006

[btag148-B63] Pinho AV , Rooman I, Reichert M et al Adult pancreatic acinar cells dedifferentiate to an embryonic progenitor phenotype with concomitant activation of a senescence programme that is present in chronic pancreatitis. Gut 2011;60:958–66. 21193456 10.1136/gut.2010.225920

[btag148-B64] Qiu P. Embracing the dropouts in single-cell RNA-seq analysis. Nat Commun 2020;11:1169. 32127540 10.1038/s41467-020-14976-9 PMC7054558

[btag148-B65] Rao A , Barkley D, França GS et al Exploring tissue architecture using spatial transcriptomics. Nature 2021;596:211–20. 34381231 10.1038/s41586-021-03634-9 PMC8475179

[btag148-B66] Rosvall M , Bergstrom CT. Maps of random walks on complex networks reveal community structure. Proc Natl Acad Sci USA 2008;105:1118–23. 18216267 10.1073/pnas.0706851105 PMC2234100

[btag148-B67] Satija R , Farrell JA, Gennert D et al Spatial reconstruction of single-cell gene expression data. Nat Biotechnol 2015;33:495–502. 25867923 10.1038/nbt.3192 PMC4430369

[btag148-B68] Szklarczyk D , Franceschini A, Wyder S et al STRING v10: protein–protein interaction networks, integrated over the tree of life. Nucleic Acids Res 2015;43:D447–52. 25352553 10.1093/nar/gku1003 PMC4383874

[btag148-B69] Tamura K , Yu J, Hata T et al Mutations in the pancreatic secretory enzymes CPA1 AND CPB1 are associated with pancreatic cancer. Proc Natl Acad Sci USA 2018;115:4767–72. 29669919 10.1073/pnas.1720588115 PMC5939087

[btag148-B70] Tian L , Dong X, Freytag S et al Benchmarking single cell RNA-sequencing analysis pipelines using mixture control experiments. Nat Methods 2019;16:479–87. 31133762 10.1038/s41592-019-0425-8

[btag148-B71] Traag VA , Waltman L, Van Eck NJ. From Louvain to Leiden: guaranteeing well-connected communities. Sci Rep 2019;9:5233–12. 30914743 10.1038/s41598-019-41695-z PMC6435756

[btag148-B72] Vallejos CA , Risso D, Scialdone A et al Normalizing single-cell RNA sequencing data: challenges and opportunities. Nat Methods 2017;14:565–71. 28504683 10.1038/nmeth.4292 PMC5549838

[btag148-B73] Van Der Maaten L , Hinton G. Visualizing data using t-SNE. J Mach Learn Res 2008;9:2579–605.

[btag148-B74] Vazquez A , Flammini A, Maritan A et al Global protein function prediction from protein–protein interaction networks. Nat Biotechnol 2003;21:697–700. 12740586 10.1038/nbt825

[btag148-B75] Veres DV , Gyurkó DM, Thaler B et al ComPPI: a cellular compartment-specific database for protein–protein interaction network analysis. Nucleic Acids Res 2015;43:D485–93. 25348397 10.1093/nar/gku1007 PMC4383876

[btag148-B76] Wang H , Lin K, Zhang Q et al HyperTMO: a trusted multi-omics integration framework based on hypergraph convolutional network for patient classification. Bioinformatics 2024;40:btae159. 38530977 10.1093/bioinformatics/btae159 PMC11212491

[btag148-B77] Wolf FA , Angerer P, Theis FJ. SCANPY: large-scale single-cell gene expression data analysis. Genome Biol 2018;19:15. 29409532 10.1186/s13059-017-1382-0 PMC5802054

[btag148-B78] Wong HY , Sheng Q, Hesterberg AB et al Single cell analysis of cribriform prostate cancer reveals cell intrinsic and tumor microenvironmental pathways of aggressive disease. Nat Commun 2022;13:6036. 36229464 10.1038/s41467-022-33780-1 PMC9562361

[btag148-B79] Yanai I , Hashimshony T. Cel-seq2—single-cell RNA sequencing by multiplexed linear amplification. In: *Single Cell Methods: Sequencing and Proteomics*. New York, NY: Springer New York, 2019, 45–56. 10.1007/978-1-4939-9240-9_4 31028631

[btag148-B80] Zeisel A , Muñoz-Manchado AB, Codeluppi S et al Cell types in the mouse cortex and hippocampus revealed by single-cell RNA-seq. Science 2015;347:1138–42. 25700174 10.1126/science.aaa1934

[btag148-B81] Zepp JA , Morrisey EE. Cellular crosstalk in the development and regeneration of the respiratory system. Nat Rev Mol Cell Biol 2019;20:551–66. 31217577 10.1038/s41580-019-0141-3 PMC7254499

[btag148-B82] Zhang C , Hu Y, Gao L. Defining and identifying cell sub-crosstalk pairs for characterizing cell–cell communication patterns. Sci Rep 2023;13:15746. 37735248 10.1038/s41598-023-42883-8 PMC10514069

[btag148-B83] Zheng GXY , Terry JM, Belgrader P et al Massively parallel digital transcriptional profiling of single cells. Nat Commun 2017;8:14049. 28091601 10.1038/ncomms14049 PMC5241818

[btag148-B84] Zheng W , Min W, Wang S. TsImpute: an accurate two-step imputation method for single-cell RNA-seq data. Bioinformatics 2023;39:btad731. 38039139 10.1093/bioinformatics/btad731 PMC10724850

[btag148-B85] Zickenrott S , Angarica V, Upadhyaya B et al Prediction of disease–gene–drug relationships following a differential network analysis. Cell Death Dis 2016;7:e2040. 26775695 10.1038/cddis.2015.393 PMC4816176

