# Supplementary material for: Hypergraph representations of single-cell RNA sequencing data for improved cell clustering
Source: Bioinformatics. 2026 Mar 27;42(4):btag148. doi: 10.1093/bioinformatics/btag148 (PMC13070707; doi:10.1093/bioinformatics/btag148)
Supplement: btag148_Supplementary_Data [file btag148_supplementary_data.pdf]

Supplementary Materials: Hypergraph Representations of Single-Cell RNA Sequencing Data for Improved Cell Clustering

Wan He, Daniel I. Bolnick, Samuel V. Scarpino, and Tina Eliassi-Rad

S1 Evaluating the Impact of Correlation Methods on Network-Based Clustering Performance

There are two primary strategies for handling shared zeros in cell expression profiles of scRNA-seq data during correlation computation. The first strategy, exemplified by cosine similarity, is to ignore the shared zeros. In this approach, inactive expression (zeros) does not contribute to the similarity between cells. However, our experiments indicate that this strategy leads to significantly worse clustering performance. The absence of expression in the same genes between cell pairs provides information about cellular similarity and should not be disregarded.

We evaluate how co-expression networks generated by different correlation computation methods affect the performance of various network-based clustering methods. See Table S1. Our results show that cosine similarity often produces the worst clustering results among Pearson, Spearman, and Kendall Tau.

**Method**	**ARI**				**NMI**			
	**Pearson**	**Cosine**	**Spearman**	**Kendall**	**Pearson**	**Cosine**	**Spearman**	**Kendall**
GreedyModularity	0.205	**0.135**	0.194	0.197	0.534	**0.429**	0.496	0.502
Louvain	0.493	**0.322**	0.445	0.473	0.784	**0.666**	0.768	0.781
Infomap	**0.000**	**0.000**	**0.000**	**0.000**	**0.000**	**0.000**	**0.000**	**0.000**
Leiden	**-0.001**	**-0.001**	-0.000	-0.000	0.062	**0.040**	0.082	0.073
Multilevel	**0.032**	**0.034**	0.034	0.035	0.305	**0.289**	0.325	0.319
Eigenvector	0.002	**0.000**	0.003	0.005	0.097	**0.060**	0.082	0.091

Table S1: Impact of Correlation Methods on Network-Based Clustering Performance. The table shows how different correlation measures (specifically, Pearson, Spearman, Kendall Tau, and cosine similarity) affect the performance of various network-based clustering methods. The correlation computation method that results in the worst clustering performance is marked in bold. Cosine similarity, which ignores shared zeros in cell expression profiles, often produces the worst clustering performance. These results indicate that while correlation computation methods that treat zeros the same as non-zero signals may inflate correlations, ignoring these zeros, as in cosine similarity, can lead to poorer performance.

The main implication of this experiment is that both strategies have limitations when handling sparse scRNA-seq data. Cosine similarity's omission of shared zeros results in the poorest performance. In contrast, methods that treat all shared zeros in expression profiles as indicators of cell

homogeneity (such as Pearson, Spearman, and Kendall Tau) overlook the fact that many shared zeros arise simply from the high dimensionality of scRNA-seq data. When sequencing results from multiple cells are combined, many zero entries appear because different cells often express different sets of genes. Our results indicate that alternative approaches, such as hypergraph-based methods, offer a better solution by avoiding the need for unipartite network projections and the complexity of handling zeros in correlation calculations.

S2 Computational and Memory Complexities of DIPHW and CoMem-DIPHW

DIPHW. The computational cost of each element $P(u \to v)$ in the $|V| \times |V|$ node-to-node transition probability matrix is $O(|E|)$ when the intermediate matrices for the node-to-edge and edge-to-node transition probabilities $P_{E|V}(e|u)$ and $P_{V|E}(v|e)$ are precomputed. The construction of the entire $|V| \times |V|$ node-to-node transition probability matrix involves computing these probabilities for all node pairs, resulting in an overall time complexity of $O(|V|^2 \cdot |E|)$.

For efficient computation, the $|V| \times |V|$ node-to-node transition probability matrix $P_{V|V}$ in martix form is given by:

$$P_{V|V} = D_{E|V}^{-1} W_{E|V} D_{V|E}^{-1} W_{V|E}$$

The theoretical complexity remains $O(|E|^2 \cdot |V| + |E| \cdot |V|^2)$. However, the matrix representation utilizes parallel computation and is significantly faster, making it more suitable for large datasets.

DIPHW's memory complexity is $O(|E| \cdot |V|)$ for the node-to-edge and edge-to-node transition probability matrices.

CoMem-DIPHW. The computational complexity of CoMem-DIPHW is $O(|E|^2 \cdot |V|^2)$. For each pair of nodes, the memory-incorporated transition traverses all possible edges that could have reached the first node and all possible edges that could then connect the first node with the second node.

CoMem-DIPHW's memory complexity is $O(|E|^2 \cdot |V| + |E| \cdot |V|^2)$ for the memory-incorporated node-to-edge and edge-to-node transition probability matrices.

S3 Bipartite representation

We choose the hypergraph conceptualization over the bipartite representation for consistency, as the DIPHW algorithm we introduce is an extension of the edge-dependent vertex weight hypergraph random walk (EDVW) [Chitra and Raphael, 2019]. DIPHW extends the EDVW hypergraph random walk by incorporating a vertex-dependent edge selection probability and a preference exponent to accelerate clustering.

A bipartite conceptualization could also be applied to our proposed methods by interpreting the incidence matrix $\mathbf{I}_{\mathcal{H}}$ of the hypergraph $\mathcal{H}$ as a bipartite graph. In this representation, the two sets of nodes are the set of cells V and the set of genes E. Each gene is connected to all cells in which it is actively expressed, with weights corresponding to the expression level. This alternative representation enables the use of bipartite graph algorithms. For example, we use Barber's bipartite

modularity [Barber, 2007] to assess how the clustering performance of different algorithms varies with the modularity of the underlying scRNA-seq dataset.

S4 Quantitative Evaluation of Clustering Quality on Tissue Datasets

The quantitative evaluations in Tables S2 and S3 offer complementary perspectives on clustering performance for tissue scRNA-seq datasets. The evaluation in the learned embedding space (Table S2) reflects the geometric separability and compactness of the clusters produced by each method, while the evaluation of the preprocessed expression data (Table S3) assesses whether the same cluster assignments capture the structure of the preprocessed gene expression data used as input to the embedding methods. In the embedding space, graph-sc and scASDC achieve strong geometric separation and cohesion across datasets. However, they show reduced clustering performance when evaluated on the preprocessed data. This aligns with the results in Fig. 12, where scASDC produces embeddings with strong cluster separation and cohesion, but some identified DEGs correspond to biologically implausible cell types. In particular, PCA performs best in the preprocessed data evaluation. This is consistent with the findings of Ciortan et al. [Ciortan and Defrance, 2022], where PCA outperformed many state-of-the-art methods, including graph-sc, when evaluated by the Silhouette score and the Calinski-Harabasz index on real and simulated datasets.

	Human Brain				Human Pancreas				Mouse Brain				Mouse Pancreas			
Method	SC	CH	DB	CV	SC	CH	DB	CV	SC	CH	DB	CV	SC	CH	DB	CV
scASDC	**0.421**	244.710	0.975	2.373	**0.763**	**7005.583**	**0.549**	0.821	**0.816**	5230.004	**0.640**	1.845	**0.874**	**2843.512**	**0.462**	0.563
graph-sc	0.352	**705.099**	**0.844**	**0.560**	0.529	4164.981	0.584	**0.816**	0.418	2431.617	0.982	**0.905**	0.394	976.784	0.768	**0.495**
CoMem_DIPHW	0.285	224.443	1.207	1.287	0.384	1048.921	1.119	1.058	0.396	1222.851	0.936	1.403	0.274	313.957	1.183	1.677
CoMem	0.265	199.820	1.285	1.922	0.474	1447.208	0.852	3.575	0.455	1432.727	0.817	1.086	0.291	287.810	1.179	4.768
tsImpute	0.263	703.410	1.163	8.200	0.457	715.814	0.789	5.051	0.409	867.564	1.095	8.499	0.424	666.677	0.902	10.673
PCA	0.258	236.768	1.412	6.802	0.294	542.832	1.270	89.515	0.229	512.127	1.652	83.396	0.107	120.386	1.804	125.705
CAKE	0.156	164.736	1.679	2.849	0.417	3999.076	0.794	1.944	0.461	**12194.111**	0.800	2.684	0.237	373.750	1.391	1.825
node2vec	0.133	172.629	2.118	2.307	0.409	1405.125	1.034	1.359	0.332	1108.268	1.188	27.464	0.198	265.955	1.467	1.014
DIPHW	0.122	110.724	2.138	0.903	0.204	480.360	1.328	1.060	0.127	264.797	2.444	5.145	0.055	35.369	3.380	3.147
EDVW	0.109	97.634	2.315	1.720	0.152	402.944	2.039	6.156	0.126	246.265	2.616	5.840	0.060	40.552	3.482	5.804

Table S2: Clustering Quality Evaluated in the Learned Embedding Space on Tissue scRNA-seq Datasets. Clustering performance is assessed using the Silhouette score (SC), Calinski–Harabasz index (CH), Davies–Bouldin index (DB), and Coefficient of Variation (CV) on the learned embeddings produced by each method. Higher values indicate better performance for the Silhouette score and Calinski–Harabasz index, while lower values indicate better performance for the Davies–Bouldin index and Coefficient of Variation. This evaluation reflects the geometric separability and cohesion of clusters in the embedding space. The best values within each dataset/evaluation measure column are highlighted in bold, and the second-best values are underlined.

S5 Additional Results for Clustering Performance Across Modularity Levels

Figures S1 and S2 show additional results on the impact of module size and module count on ARI clustering performance. Figures S3 and S4 show the impact of module size and module count on NMI clustering performance. Figures S5 and S6 show the impact of module size and module count on ACC clustering performance. Figures S7 and S8 show the impact of module size and module

Method	Human Brain				Human Pancreas				Mouse Brain				Mouse Pancreas			
	SC	CH	DB	CV	SC	CH	DB	CV	SC	CH	DB	CV	SC	CH	DB	CV
CoMem	**0.027**	17.529	4.654	3.951	0.071	230.969	2.048	4.559	0.041	173.331	2.838	7.603	-0.002	37.954	4.182	5.064
CoMem_DIPHW	0.027	17.529	**4.575**	3.951	0.021	235.958	2.364	4.834	0.018	169.640	3.363	7.795	0.002	40.049	3.891	5.008
PCA	0.025	**19.080**	4.991	4.012	**0.276**	**395.673**	**1.461**	4.012	**0.087**	**183.428**	**2.829**	6.995	0.016	**46.001**	**3.051**	**4.699**
tsImpute	0.009	12.901	7.122	3.958	0.114	171.384	2.715	**3.734**	-0.005	67.857	6.082	6.721	-0.025	22.239	6.907	5.127
graph-sc	0.008	10.692	6.286	4.043	0.042	201.025	2.218	4.137	0.047	127.852	5.446	6.905	**0.052**	28.514	3.696	4.749
node2vec	0.007	15.731	8.386	4.010	0.060	259.873	2.019	4.609	0.040	174.822	3.240	7.726	-0.001	37.939	4.459	5.027
DIPHW	0.006	14.937	7.794	4.018	0.015	195.839	3.152	4.328	0.012	153.568	4.081	7.782	-0.006	36.516	5.353	5.115
EDVW	0.002	13.243	8.843	4.042	-0.075	177.732	7.734	4.956	0.034	172.331	3.403	7.805	-0.001	39.774	4.790	4.991
scASDC	-0.009	7.670	7.899	**3.947**	-0.128	139.358	3.605	4.655	0.070	166.681	3.032	7.177	-0.027	26.217	6.188	5.148
CAKE	-0.012	7.713	8.723	4.002	-0.025	184.071	3.063	4.444	0.068	75.122	6.077	**5.624**	0.006	33.657	6.005	5.042

Table S3: Clustering Quality Evaluated on Preprocessed Expression Data for Tissue scRNA-seq Datasets. Using the same cluster assignments as in Supplementary Table S2, we evaluate cluster quality on the preprocessed gene expression data used as input to each embedding method. We measure cluster quality using the Silhouette score (SC), Calinski–Harabasz index (CH), Davies–Bouldin index (DB), and Coefficient of Variation (CV). Higher values indicate better performance for SC and CH, while lower values indicate better performance for DB and CV. This evaluation assesses whether the cluster assignments by each method capture meaningful structure in the input gene expression data. The best values within each dataset/evaluation measure column are highlighted in bold, and the second-best values are underlined.

count on AMI clustering performance. Figures S9 and S10 show the impact of module size and module count on F1 clustering performance. Finally, Fig. S11 shows a visualization of modularity in our imulated scRNA-seq Data.

S6 Results for the Human Brain Dataset

Figures S12, S13, S14, S15, S16, and S17 show the results on the human brain dataset for CoMem-DIPHW, PCA, graph-sc, tsImpute, CAKE, and scASDC, respectively.

S7 Results for the Mouse Pancreas and the Mouse Brain Datasets

Figures S18, S19, S20, S21, S22, and S23 show the results on the mouse pancreas dataset for CoMem-DIPHW, PCA, graph-sc, tsImpute, CAKE, and scASDC, respectively.

Figures S24, S25, S26, S27, S28, and S29 show the results on the mouse brain dataset for CoMem-DIPHW, PCA, graph-sc, tsImpute, CAKE, and scASDC, respectively.

S8 Hyperparameters

Table S4 lists the hyperparameters used in each experiment.

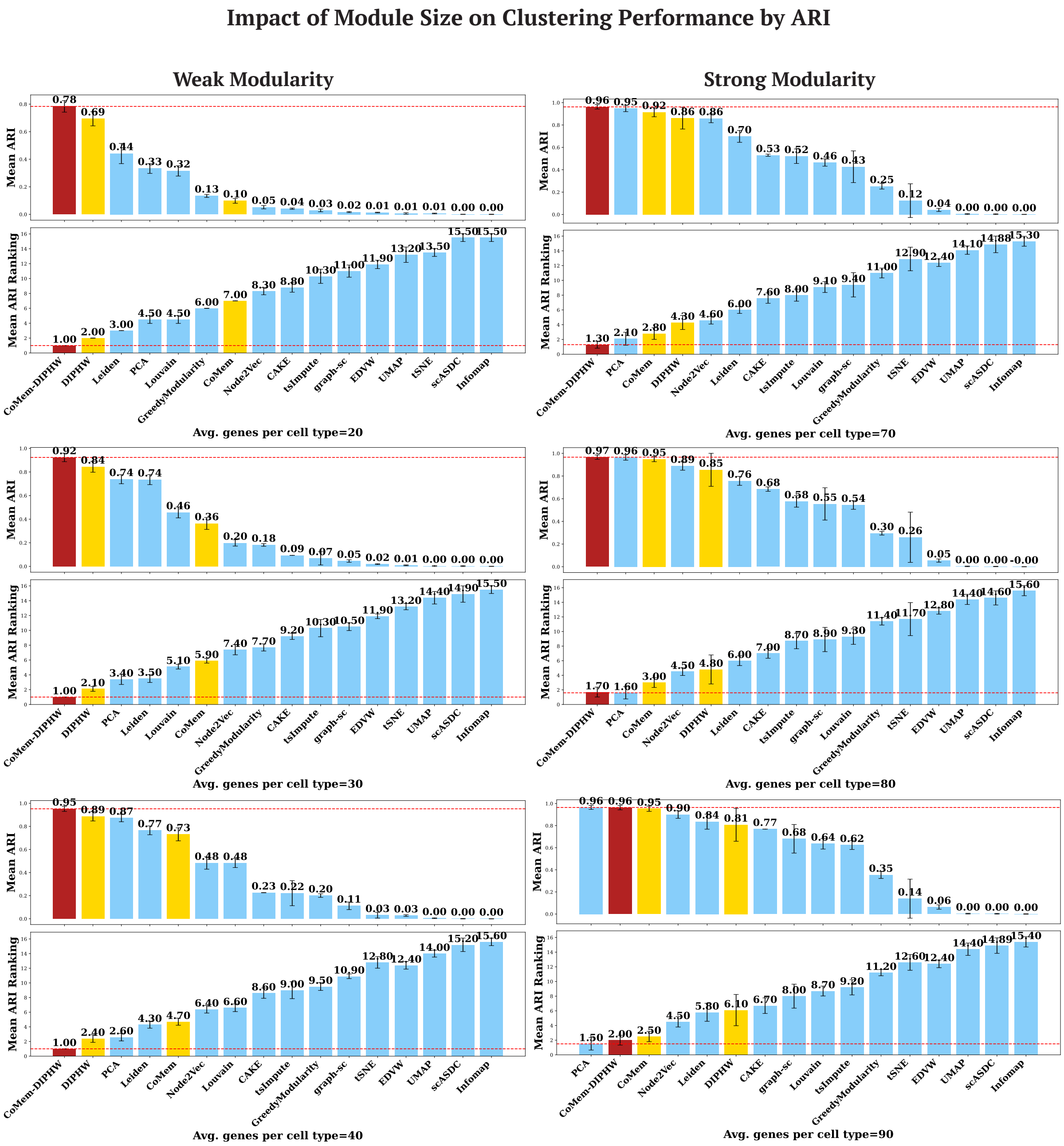

Figure S1: Clustering Performance Comparison by ARI Across Varying Module Sizes. Simulated scRNA-seq data were used for this evaluation. Here, we present the complementary results across intermediate parameter settings, which show consistent patterns: our proposed methods (highlighted in red and yellow) consistently ranked first by ARI in all scenarios, with a stronger advantage when data modularity is weak, i.e., when the average number of co-expressed genes per module is small. Each experiment was repeated 10 times per parameter setting, with error bars representing the 95% confidence interval. Red dashed lines indicate the highest ARI values or best ARI rankings. K-means was used to cluster the output of all embedding-based methods that do not directly assign cluster membership.

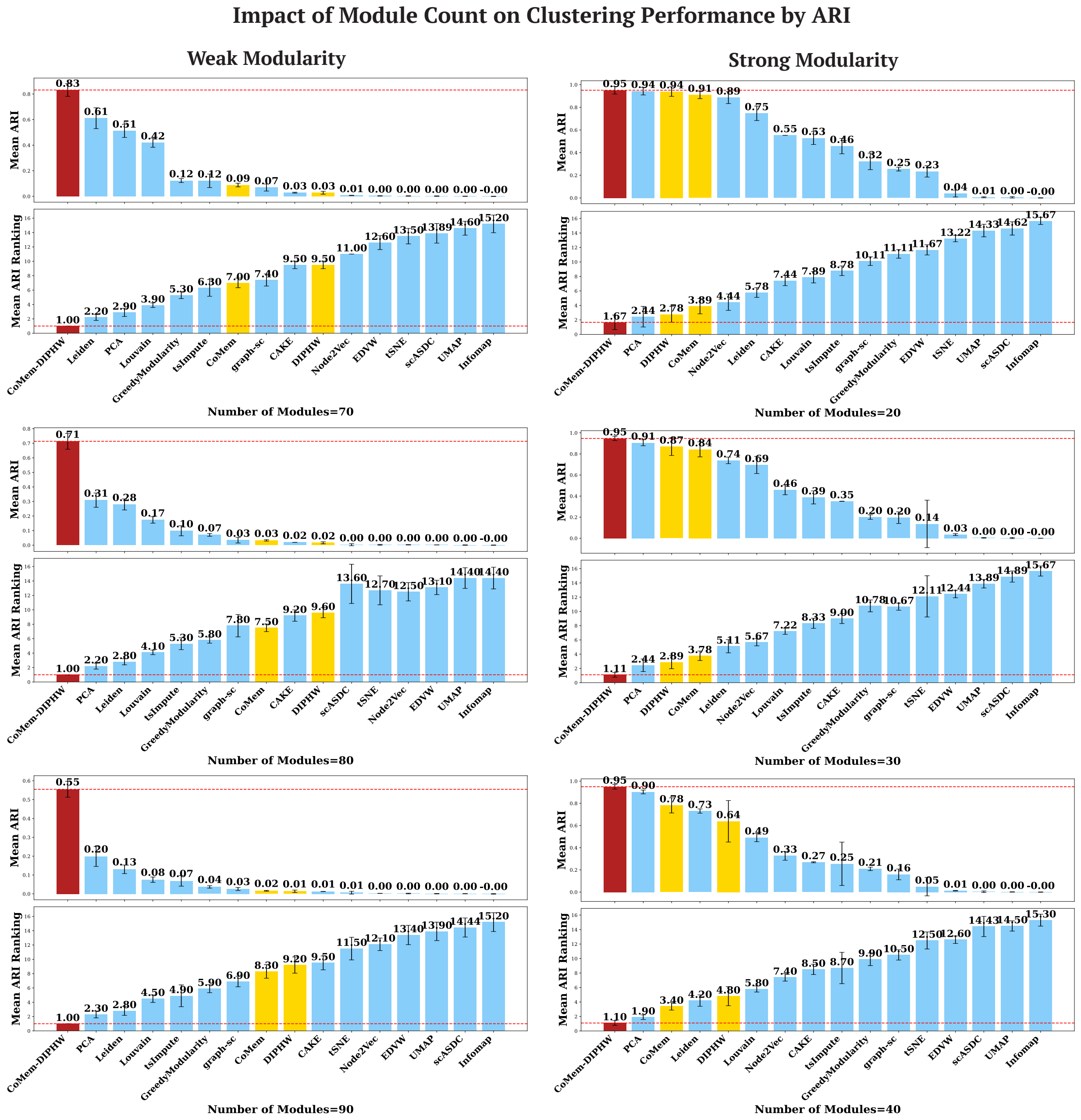

Figure S2: Clustering Performance Comparison by ARI Across Varying Module Counts. Simulated scRNA-seq data were used for this evaluation. Here, we present the complementary results across intermediate parameter settings, which show consistent patterns: our proposed methods (highlighted in red and yellow) consistently ranked first by ARI in all scenarios, with a stronger advantage when data modularity is weak, i.e., when the number of embedded modules in the simulated scRNA-seq data is greater. Each experiment was repeated 10 times per parameter setting, with error bars representing the 95% confidence interval. Red dashed lines indicate the highest ARI values or best ARI rankings. K-means was used to cluster the output of all embedding-based methods that do not directly assign cluster membership.

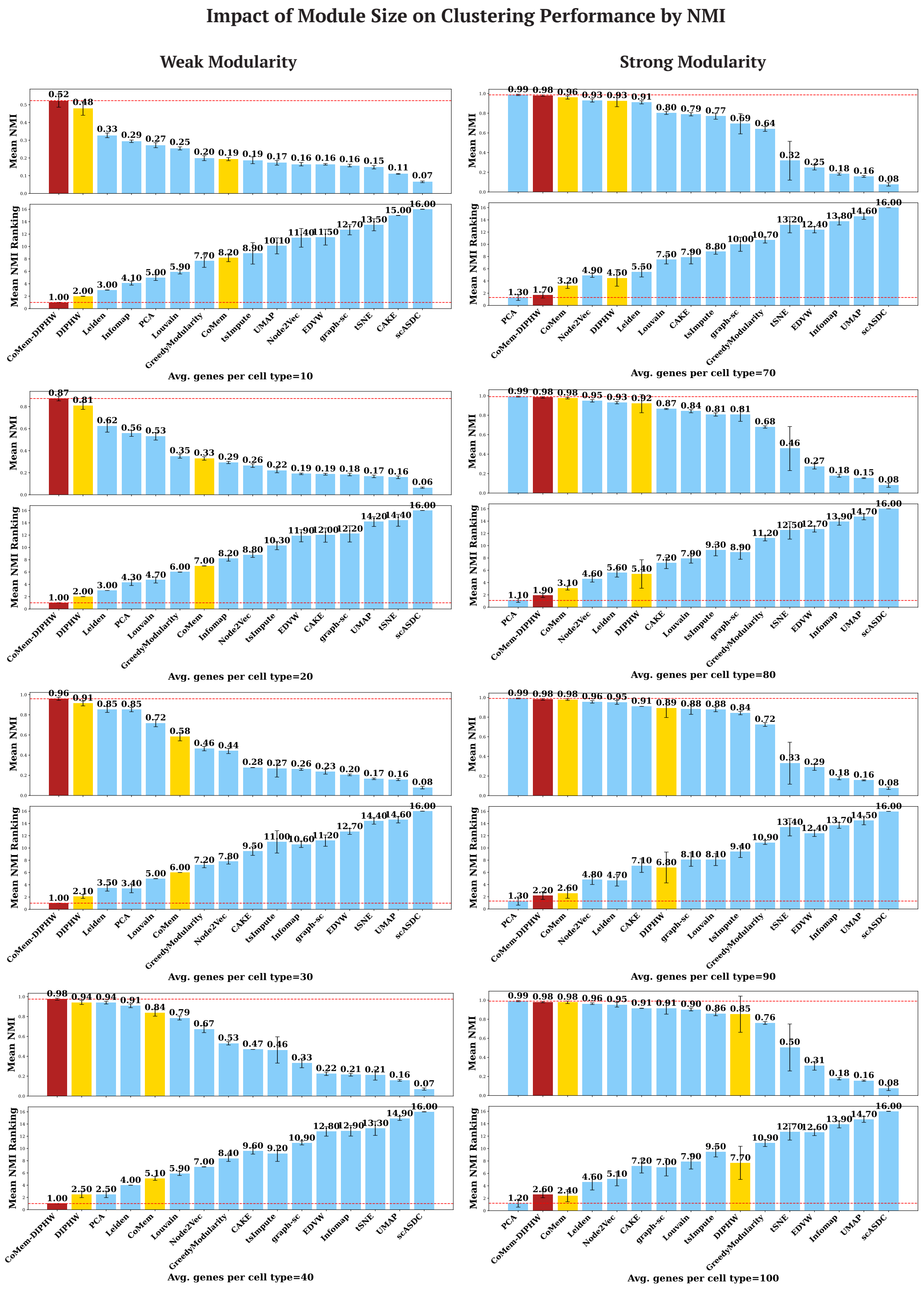

Figure S3: Clustering Performance Comparison by NMI Across Varying Module Sizes. Simulated scRNA-seq data were used for this evaluation. The NMI results support the same conclusion: when modularity is weak (i.e., when the average number of co-expressed genes per module is small), the advantage of our proposed methods (highlighted in red and yellow) is more pronounced. Each experiment was repeated 10 times per parameter setting, with error bars representing the 95% confidence interval. Red dashed lines indicate the highest NMI values or best NMI rankings. K-means was used to cluster the output of all embedding-based methods that do not directly assign cluster membership.

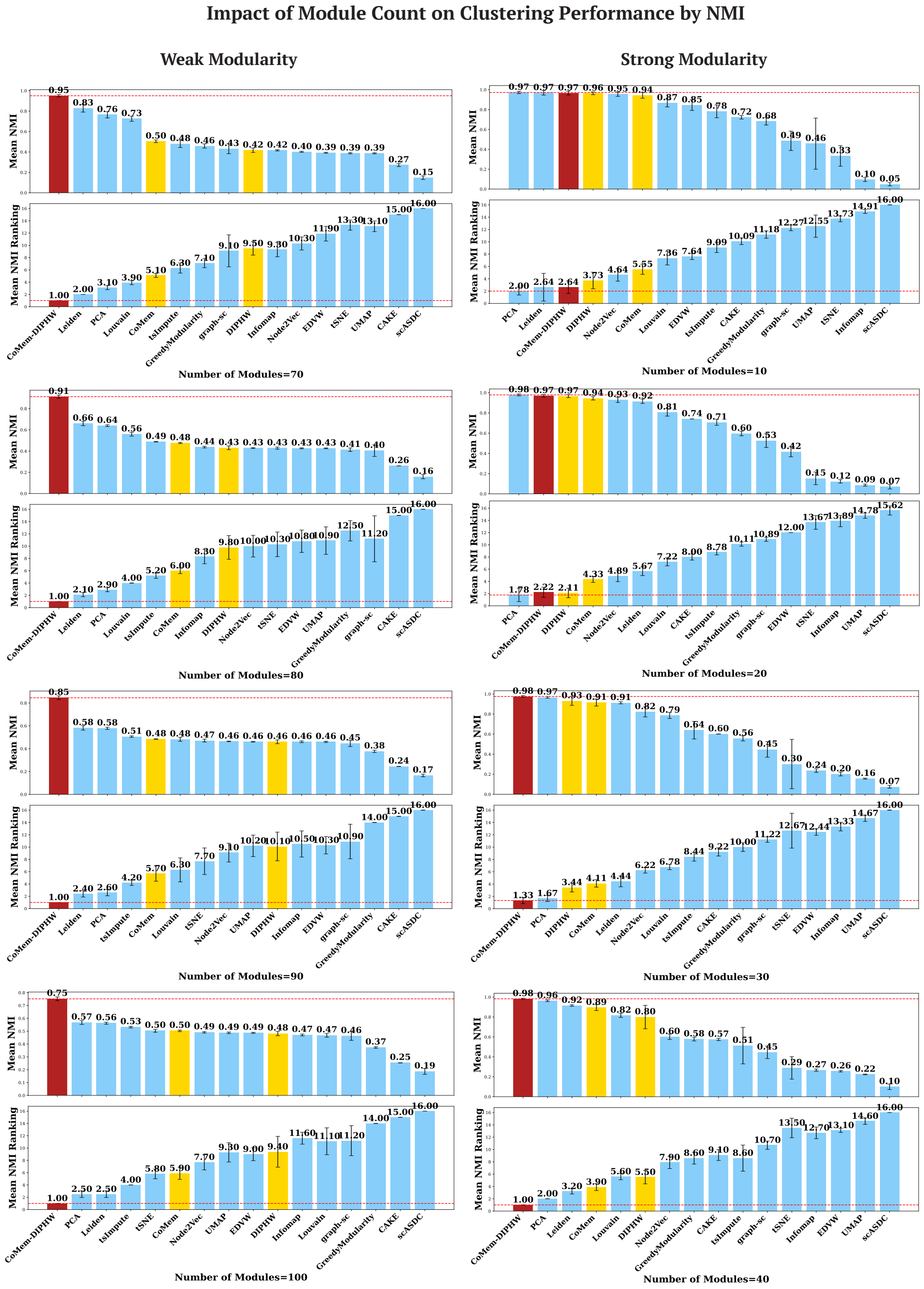

Figure S4: Clustering Performance Comparison by NMI Across Varying Module Counts. Simulated scRNA-seq data were used for this evaluation. The results by NMI support the same conclusion: when modularity is weak (i.e., when the number of modules is greater), the advantage of our proposed methods (highlighted in red and yellow) is more pronounced. Each experiment was repeated 10 times per parameter setting, with error bars representing the 95% confidence interval. Red dashed lines indicate the highest NMI values or best NMI rankings. K-means was used to cluster the output of all embedding-based methods that do not directly assign cluster membership.

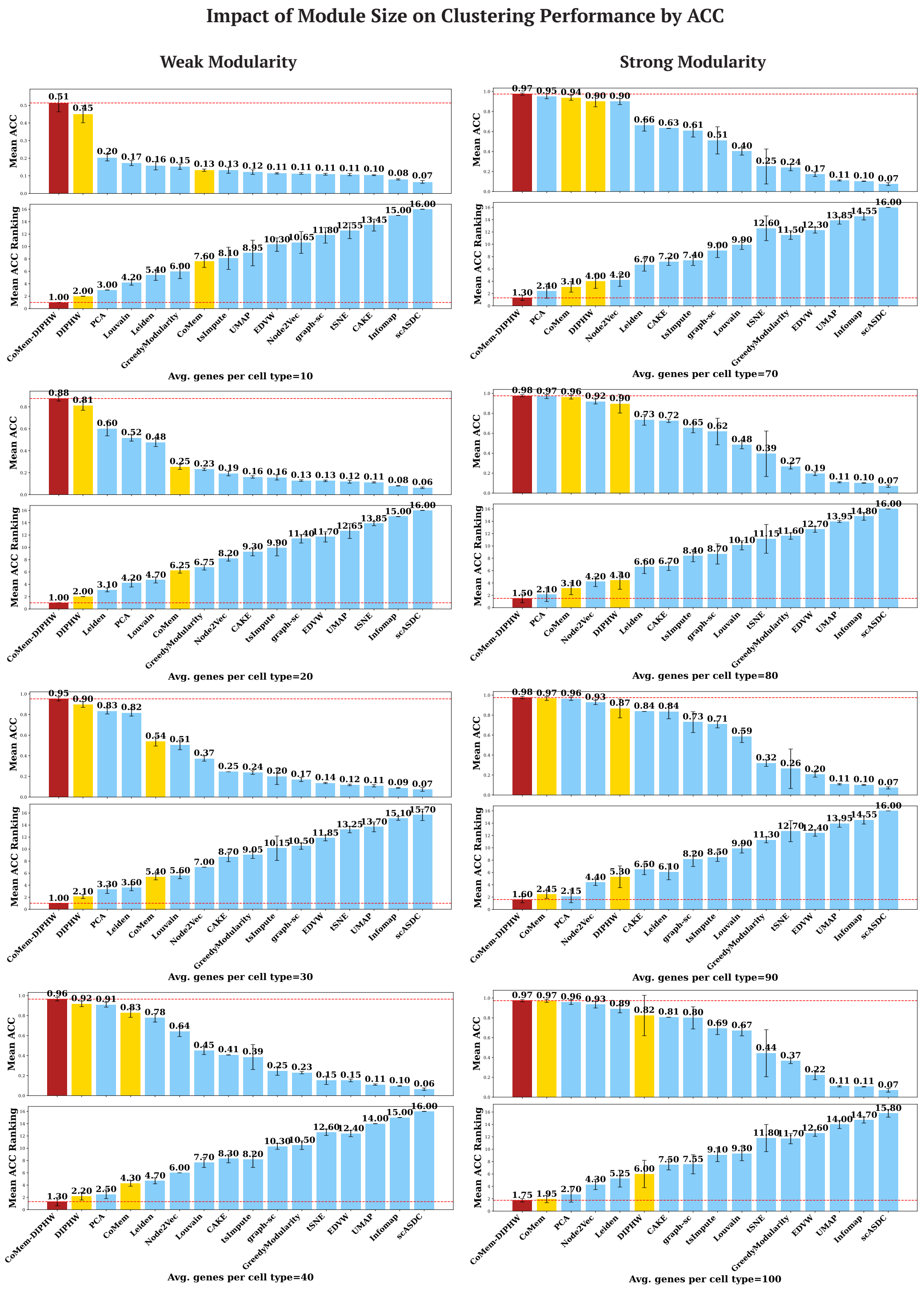

Figure S5: Clustering Performance Comparison by ACC Across Varying Module Sizes. Simulated scRNA-seq data were used for this evaluation. The ACC results support the same conclusion: when modularity is weak (i.e., when the average number of co-expressed genes per module is small), the advantage of our proposed methods (highlighted in red and yellow) is more pronounced. Each experiment was repeated 10 times per parameter setting, with error bars representing the 95% confidence interval. Red dashed lines indicate the highest ACC values or best ACC rankings. K-means was used to cluster the output of all embedding-based methods that do not directly assign cluster membership.

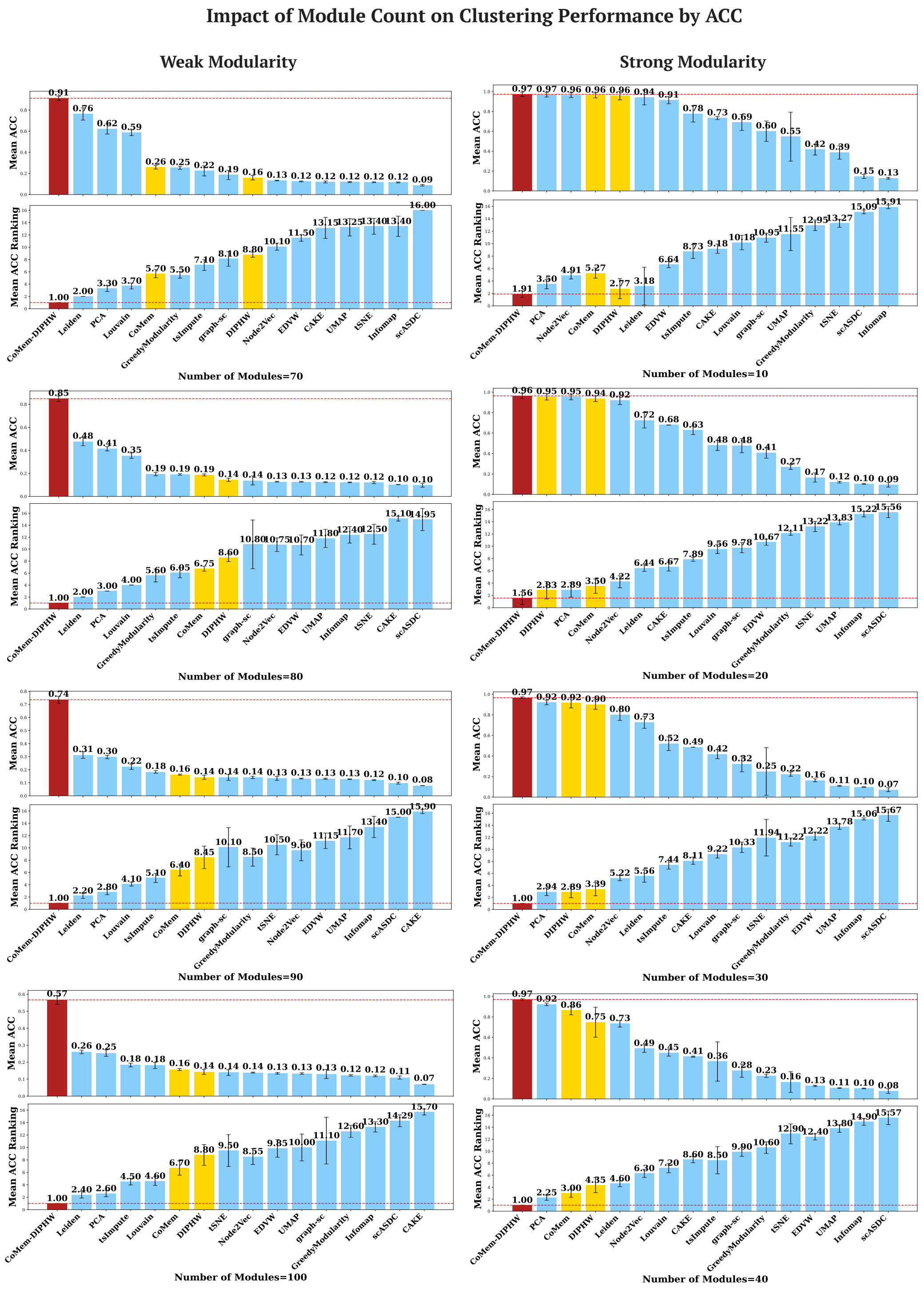

Figure S6: Clustering Performance Comparison by ACC Across Varying Module Counts. Simulated scRNA-seq data were used for this evaluation. The results by ACC support the same conclusion: when modularity is weak (i.e., when the number of modules is greater), the advantage of our proposed methods (highlighted in red and yellow) is more pronounced. Each experiment was repeated 10 times per parameter setting, with error bars representing the 95% confidence interval. Red dashed lines indicate the highest ACC values or best ACC rankings. K-means was used to cluster the output of all embedding-based methods that do not directly assign cluster membership.

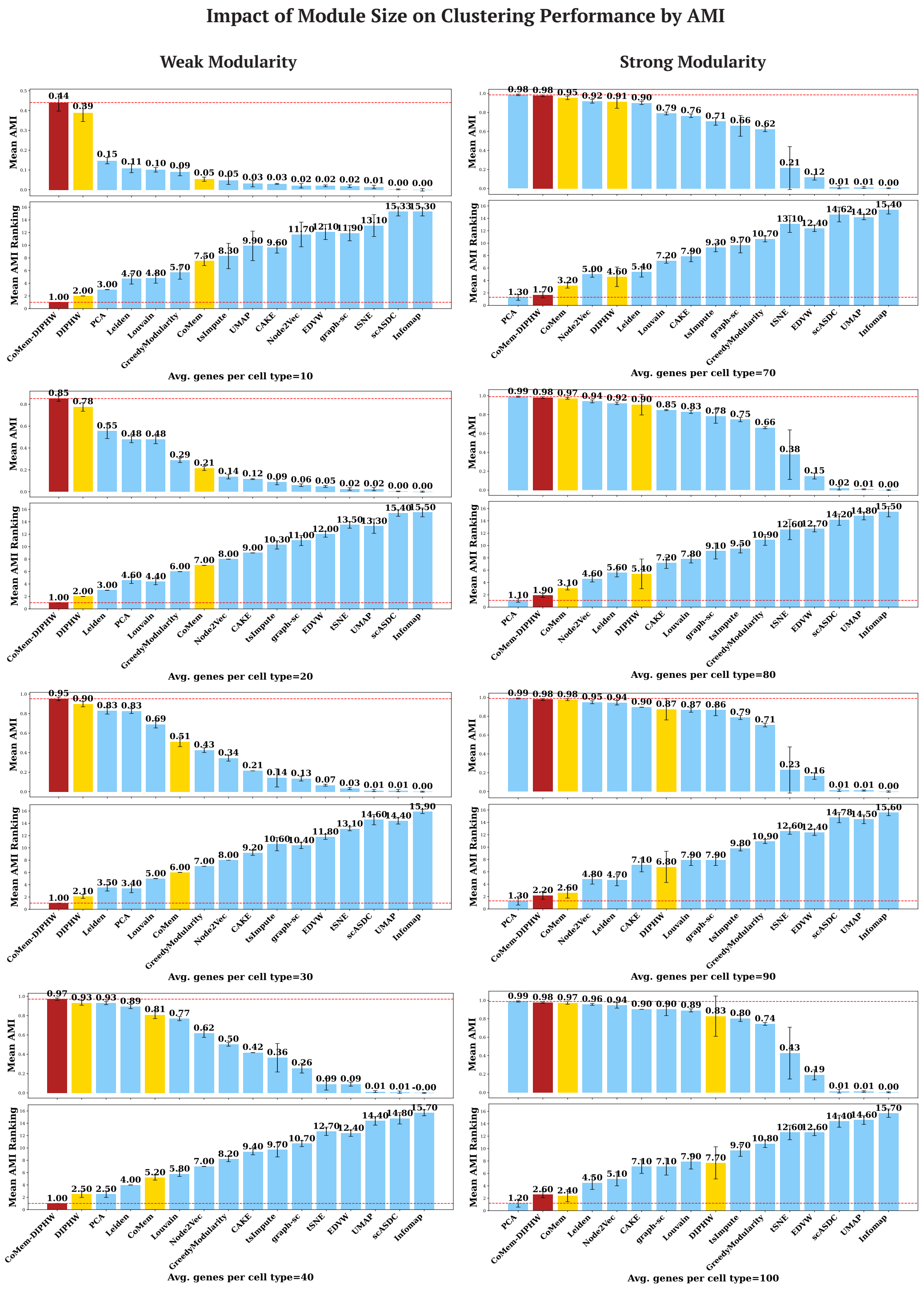

Figure S7: Clustering Performance Comparison by AMI Across Varying Module Sizes. Simulated scRNA-seq data were used for this evaluation. The AMI results support the same conclusion: when modularity is weak (i.e., when the average number of co-expressed genes per module is small), the advantage of our proposed methods (highlighted in red and yellow) is more pronounced. Each experiment was repeated 10 times per parameter setting, with error bars representing the 95% confidence interval. Red dashed lines indicate the highest AMI values or best AMI rankings. K-means was used to cluster the output of all embedding-based methods that do not directly assign cluster membership.

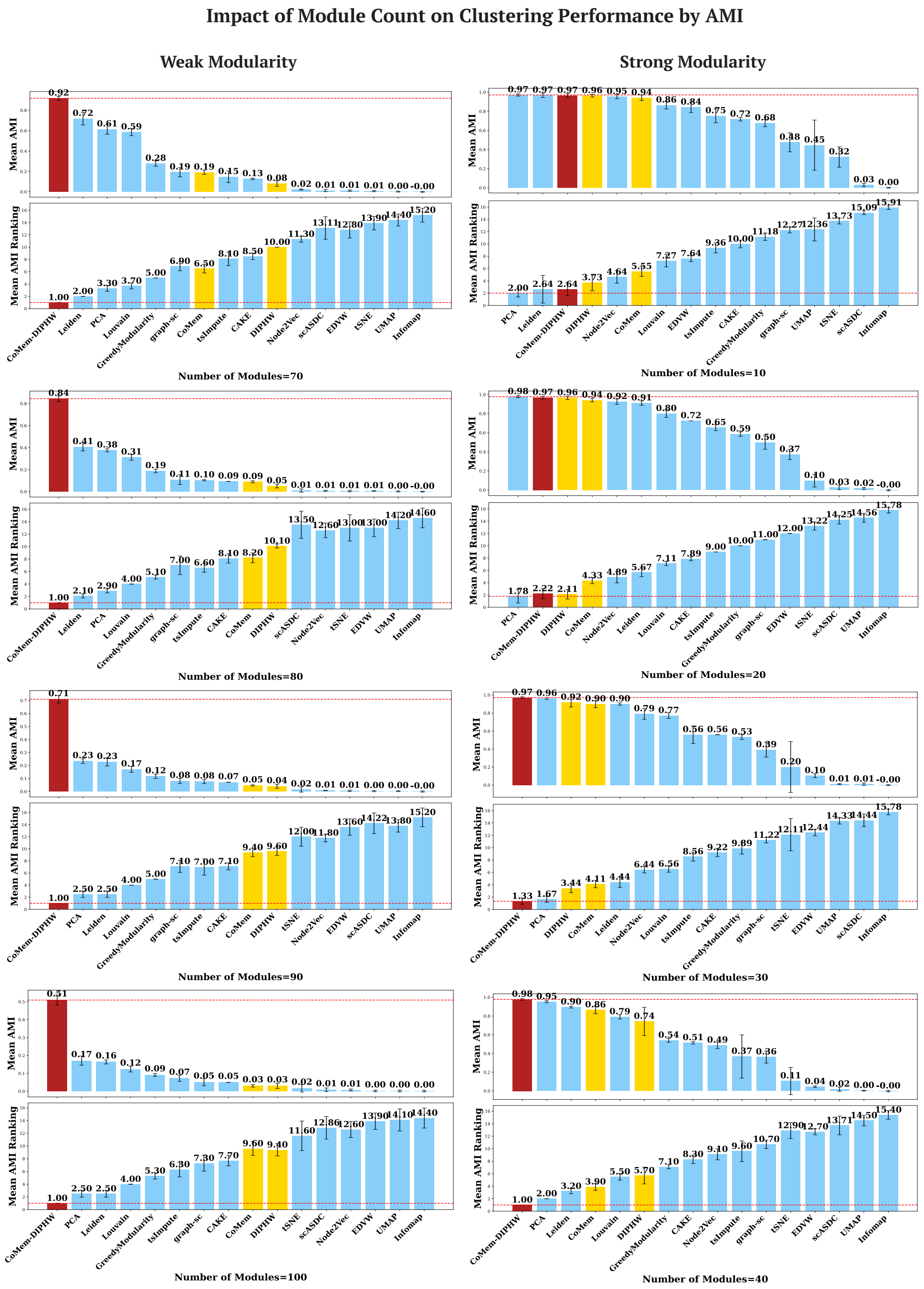

Figure S8: Clustering Performance Comparison by AMI Across Varying Module Counts. Simulated scRNA-seq data were used for this evaluation. The results by AMI support the same conclusion: when modularity is weak (i.e., when the number of modules is greater), the advantage of our proposed methods (highlighted in red and yellow) is more pronounced. Each experiment was repeated 10 times per parameter setting, with error bars representing the 95% confidence interval. Red dashed lines indicate the highest AMI values or best AMI rankings. K-means was used to cluster the output of all embedding-based methods that do not directly assign cluster membership.

Impact of Module Size on Clustering Performance by F1

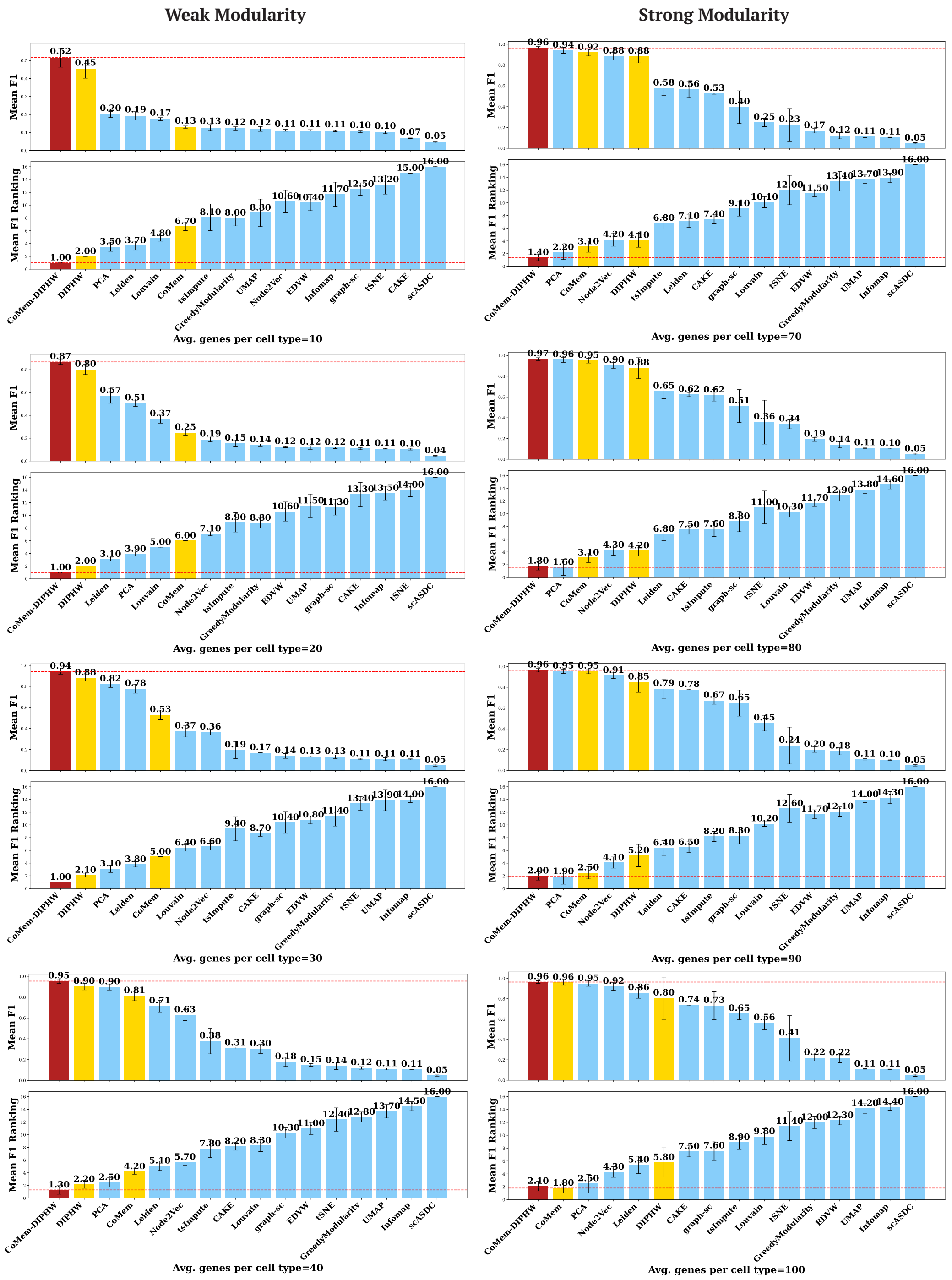

Figure S9: Clustering Performance Comparison by F1 Across Varying Module Sizes. Simulated scRNA-seq data were used for this evaluation. The F1 results support the same conclusion: when modularity is weak (i.e., when the average number of co-expressed genes per module is small), the advantage of our proposed methods (highlighted in red and yellow) is more pronounced. Each experiment was repeated 10 times per parameter setting, with error bars representing the 95% confidence interval. Red dashed lines indicate the highest F1 values or best F1 rankings. K-means was used to cluster the output of all embedding-based methods that do not directly assign cluster membership.

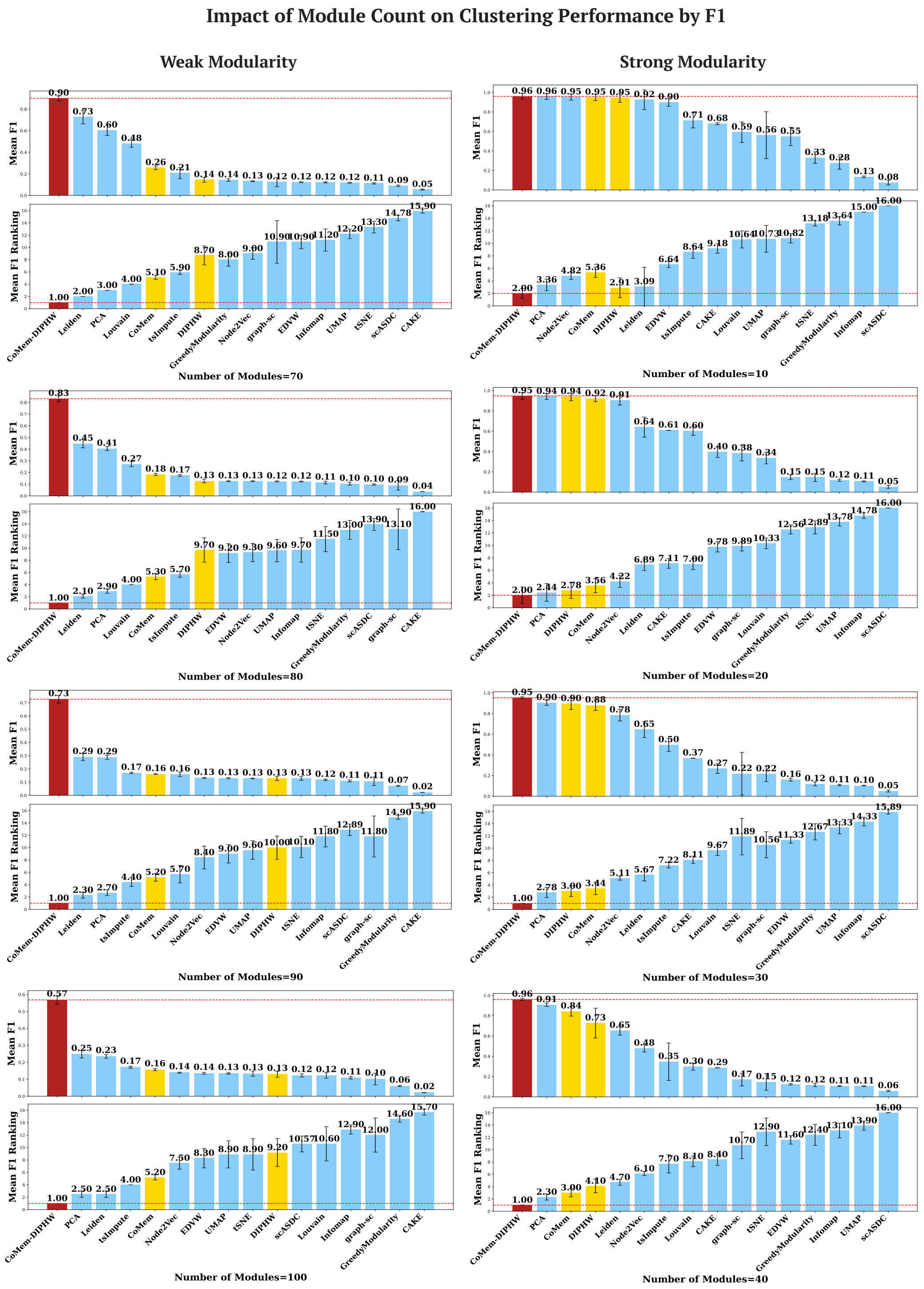

Figure S10: Clustering Performance Comparison by F1 Across Varying Module Counts. Simulated scRNA-seq data were used for this evaluation. The results by F1 support the same conclusion: when modularity is weak (i.e., when the number of modules is greater), the advantage of our proposed methods (highlighted in red and yellow) is more pronounced. Each experiment was repeated 10 times per parameter setting, with error bars representing the 95% confidence interval. Red dashed lines indicate the highest F1 values or best F1 rankings. K-means was used to cluster the output of all embedding-based methods that do not directly assign cluster membership.

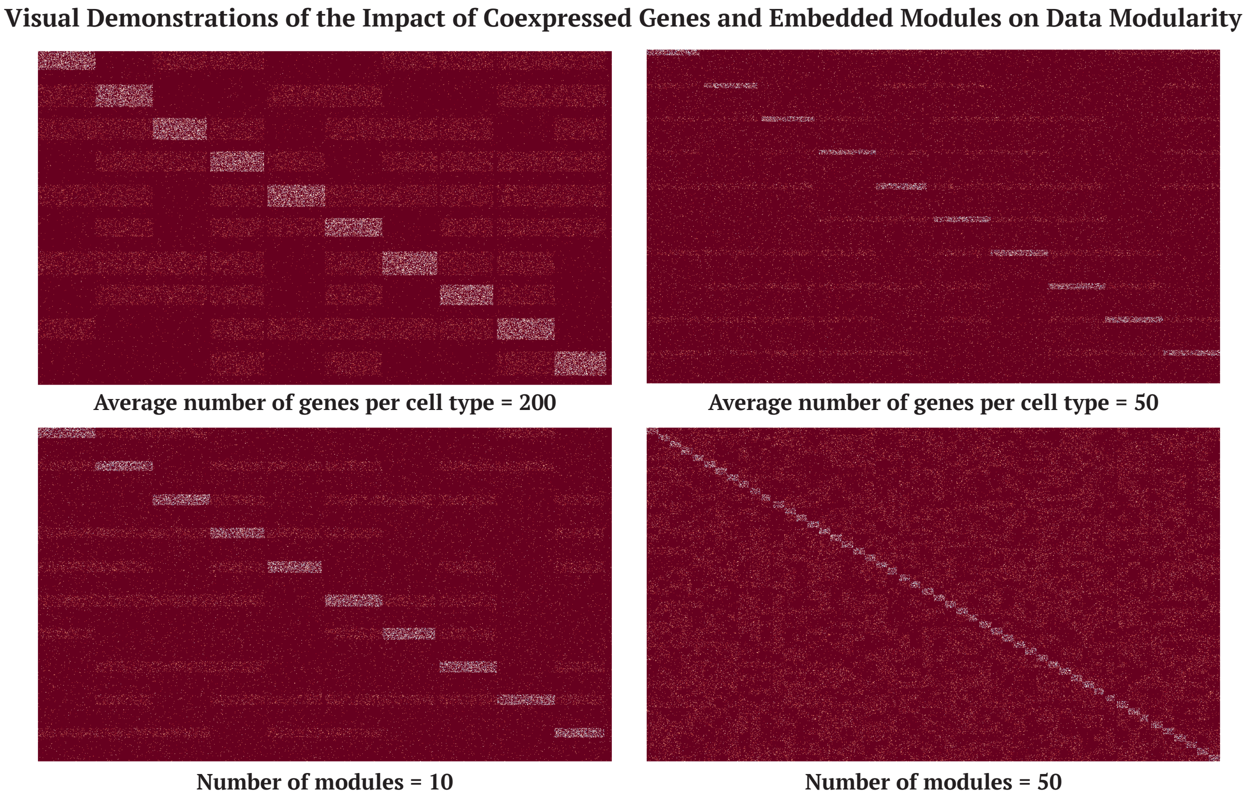

Figure S11: Visualization of Modularity in Simulated scRNA-seq Data. Modularity increases with a higher average number of co-expressed genes per cell type and fewer modules. The number of modules models the number of cell types in the simulated data.

(a) Top 10 DE Genes for Each Cluster Identified by CoMem-DIPHW in Human Brain Data

Cluster 0	Cluster 1	Cluster 2	Cluster 3	Cluster 4	Cluster 5	Cluster 6
SLC1A3	SOX4	SOX5	GDAP1L1	UCP2	COL1A2	PET100
PTPRZ1	TAGLN3	PDE1A	LHX9	HSD17B4	LDHA	SCG3
SAT1	NNAT	STMN2	SCN3A	STYXL1	EIF2A	SOX4
DOK5	STMN1	BCL11A	CACNA2D1	ACTL6A	MFAP4	GSTA4
C1orf61	SORBS2	GPM6A	MAB21L1	SELT	NR4A1	STMN1
CREB5	STMN2	GAP43	EBF1	UBE2C	COL3A1	OCIAD2
NFIA	ELAVL4	DAB1	SLC17A6	TRMT11	MGP	GAD1
CKB	BCL11A	ST18	MGAT4C	FUT10	PABPC4	STMN2
DCLRE1C	GPM6A	RUNX1T1	NEFM	MEIS1	S100A11	CD24
MASP1	CD24	GRIA2	STMN2	VIMP	LGALS1	CADPS

(b) Average Expression of Top 10 DE Genes from Each Cluster Found by CoMem-DIPHW

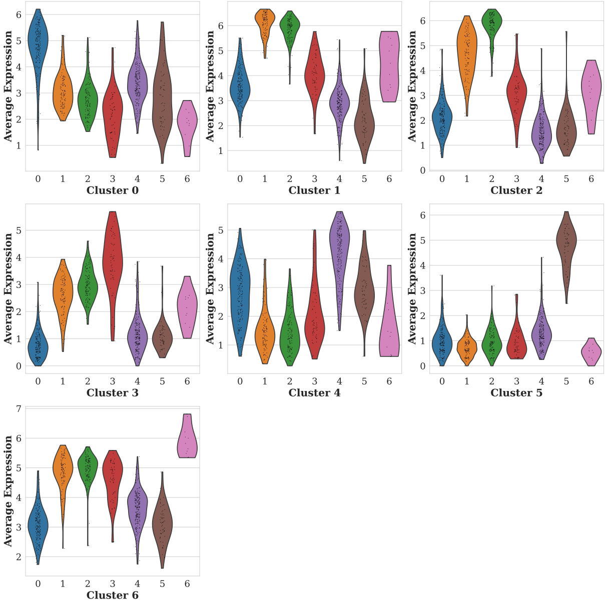

(c) Top Cell Type Matches for Each Cluster Identified by CoMem-DIPHW Based on PanglaoDB Markers

Cluster	Primary Match	Secondary Match
0	Radial glia cells (0.1429)	Glutaminergic neurons (0.1000)
1	Nuocytes (0.0833)	Parietal cells (0.0714)
2	Nuocytes (0.0833)	Epiblast cells (0.0588)
3	Purkinje fiber cells (0.2000)	Glutaminergic neurons (0.1000)
4	Gamma delta T cells (0.0606)	-
5	Meningeal cells (0.1111)	Pancreatic stellate cells (0.1034)
6	Parietal cells (0.0714)	GABAergic neurons (0.0667)

Figure S12: Clustering Performance of CoMem-DIPHW on the Human Brain Dataset and Cell Type Annotation using DEGs and Canonical Markers. (a) Top 10 DEGs identified for each cluster by CoMem-DIPHW. (b) Across-cluster average expression of cluster-specific DEGs. Violin plots show the distribution of average expression levels of these DEGs across all clusters. Strong clustering performance is indicated by high expression of cluster-specific DEGs within their respective clusters and low expression in other clusters. (c) Cell type annotation using the PanglaoDB marker database. Cell types are determined by the overlap between each cluster's DEGs and cell type-specific markers in the PanglaoDB database, with match scores computed based on the proportion of matched markers.

(a) Top 10 DE Genes for Each Cluster Identified by PCA in Human Brain Data

Cluster 0	Cluster 1	Cluster 2	Cluster 3	Cluster 4	Cluster 5	Cluster 6
NEDD4L	LMO7	SOX4	UCP2	COL1A2	GDAP1L1	EIF2A
SLC1A3	PDE1A	STMN1	HSD17B4	LDHA	SCN3A	MAB21L1
PTPRZ1	BCL11A	SORBS2	STYXL1	EIF2A	SCG3	C8orf46
DOK5	GPM6A	STMN2	ACTL6A	MFAP4	SOX4	TRIB3
C1orf61	GAP43	ELAVL4	SELT	NR4A1	TAGLN3	TAL2
CREB5	DAB1	BCL11A	UBE2C	COL3A1	NNAT	PABPC4
SHISA2	LMO3	CD24	TRMT11	MGP	STMN1	FUT10
NFIA	ST18	GPM6A	FUT10	PABPC4	NEFM	MTO1
CKB	RUNX1T1	GAP43	MEIS1	S100A11	STMN2	MARS
DCLRE1C	CAMKV	MAP2	VIMP	LGALS1	ELAVL4	CKB

(b) Average Expression of Top 10 DE Genes from Each Cluster Found by PCA

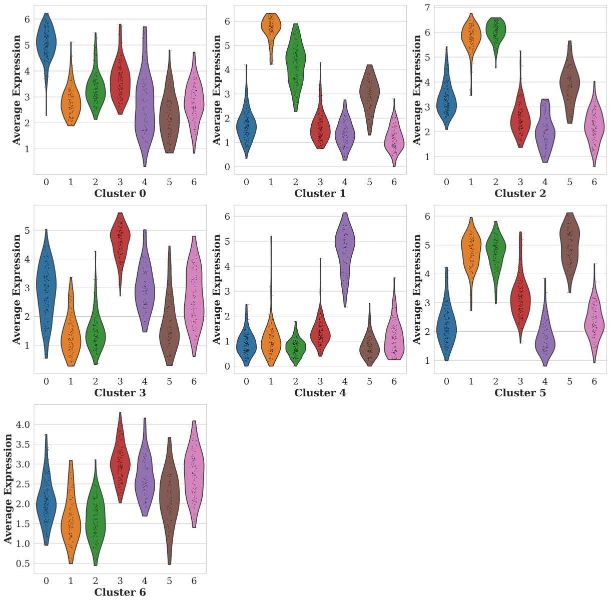

(c) Top Cell Type Matches for Each Cluster Identified by PCA Based on PanglaoDB Markers

Cluster	Primary Match	Secondary Match
0	Radial glia cells (0.1429)	Glutaminergic neurons (0.1000)
1	Nuocytes (0.0833)	Epiblast cells (0.0588)
2	Nuocytes (0.0833)	Parietal cells (0.0714)
3	Gamma delta T cells (0.0606)	-
4	Meningeal cells (0.1111)	Pancreatic stellate cells (0.1034)
5	Purkinje fiber cells (0.2000)	T follicular helper cells (0.0769)
6	No annotation	-

Figure S13: Clustering Performance of PCA on the Human Brain Dataset and Cell Type Annotation using DEGs and Canonical Markers. (a) Top 10 DEGs identified for each cluster by PCA. (b) Across-cluster average expression of cluster-specific DEGs. Violin plots show the distribution of average expression levels of these DEGs across all clusters. Strong clustering performance is indicated by high expression of cluster-specific DEGs within their respective clusters and low expression in other clusters. (c) Cell type annotation using the PanglaoDB marker database. Cell types are determined by the overlap between each cluster's DEGs and cell type-specific markers in the PanglaoDB database, with match scores computed based on the proportion of matched markers.

(a) Top 10 DE Genes for Each Cluster Identified by graph-sc in Human Brain Data

Cluster 0	Cluster 1	Cluster 2	Cluster 3	Cluster 4	Cluster 5
PAX6	NEUROD6	VIMP	COL1A2	DCN	SCN2A
HMGB2	RTN1	FLRT3	DCN	LUM	SCN3A
CDK1	STMN2	CCNB1	LGALS1	COL1A2	RALYL
SLC1A3	NFIB	UBE2C	COL3A1	COL3A1	GRIA2
CREB5	MYT1L	TXNRD1	LUM	S100A11	ANK3
KNTC1	BCL11A	MAPK10	POSTN	MFAP4	THSD7A
TOP2A	BCL11B	FSIP2	S100A11	LGALS1	CDH13
UBE2C	NFIA	TOP2A	MGP	POSTN	HMP19
CCNB1	SOX4	OSGEP	MFAP4	SELM	ANK2
DOK5	RUNX1T1	FUT10	COL21A1	MGP	SYN3

(b) Average Expression of Top 10 DE Genes from Each Cluster Found by graph-sc

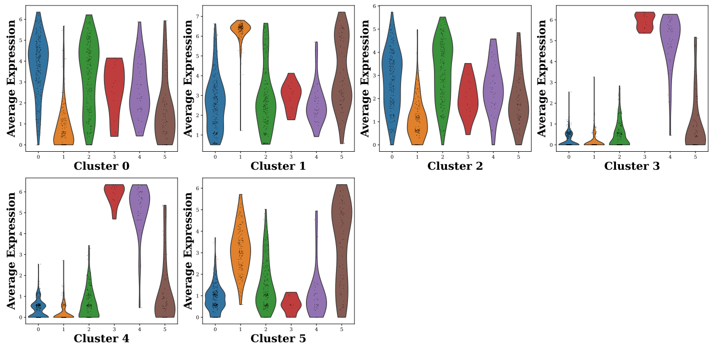

(c) Top Cell Type Matches for Each Cluster Identified by graph-sc Based on PanglaoDB Markers

Cluster	Match Type	Annotation	Score
0	Primary	Radial glia cells	0.1429
	Secondary	Retinal progenitor cells	0.0769
1	Primary	Nuocytes	0.0833
	Secondary	Epiblast cells	0.0588
2	Primary	No annotation	–
3	Primary	Meningeal cells	0.1111
	Secondary	Pancreatic stellate cells	0.1034
4	Primary	Meningeal cells	0.1111
	Secondary	Pancreatic stellate cells	0.1034
5	Primary	No annotation	–

Figure S14: Clustering Performance of graph-sc on the Human Brain Dataset and Cell Type Annotation using DEGs and Canonical Markers. (a) Top 10 DEGs identified for each cluster by graph-sc. (b) Across-cluster average expression of cluster-specific DEGs. Violin plots show the distribution of average expression levels of these DEGs across all clusters. Strong clustering performance is indicated by high expression of cluster-specific DEGs within their respective clusters and low expression in other clusters. (c) Cell type annotation using the PanglaoDB marker database. Cell types are determined by the overlap between each cluster's DEGs and cell type-specific markers in the PanglaoDB database, with match scores computed based on the proportion of matched markers. Two of the graph-sc clusters had less than 5 cells, one was excluded from the expression plots in (b), and both failed to map to known cell types in (c).

(a) Top 10 DE Genes for Each Cluster Identified by TsImpute in Human Brain Data

Cluster 1	Cluster 2	Cluster 3	Cluster 4	Cluster 5	Cluster 6	Cluster 7
NFIB	HMGB2	UBE2C	NEUROD6	COL3A1	SOX4	CKB
SOX4	PAX6	CDK1	RTN1	COL1A2	BCL11A	SOX4
STMN1	SLC1A3	CCNB1	GAP43	DCN	MAP2	STMN1
STMN2	CREB5	TOP2A	BCL11A	LUM	KIDINS220	SPAG9
NEUROD6	KNTC1	C11orf31	BCL11B	S100A11	NFIB	MAP2
GPM6A	IFI44L	UCP2	STMN2	LGALS1	AKAP9	C1orf61
NFIA	TOP2A	HMGB2	MYT1L	MFAP4	C1orf61	UBB
MAP2	CDK1	KNTC1	NFIB	POSTN	ANK3	SCN2A
RTN1	DOK5	TXNRD1	SLC24A2	SELM	ANK2	AKAP9
MYT1L	SAT1	SELT	SLA	MGP	RUNX1T1	NNAT

(b) Average Expression of Top 10 DE Genes from Each Cluster Found by TsImpute

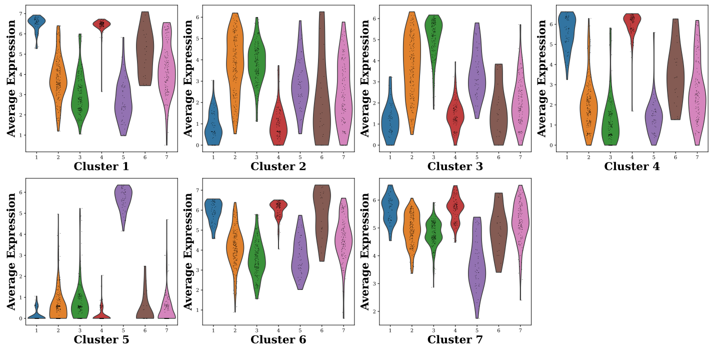

(c) Top Cell Type Matches for Each Cluster Identified by TsImpute Based on PanglaoDB Markers

Cluster	Match Type	Annotation	Score
1	Primary	No annotation	–
2	Primary	Radial glia cells	0.1429
	Secondary	Retinal progenitor cells	0.0769
3	Primary	Gamma delta T cells	0.0606
4	Primary	Nuocytes	0.0833
	Secondary	Epiblast cells	0.0588
5	Primary	Meningeal cells	0.1111
	Secondary	Pancreatic stellate cells	0.1034
6	Primary	Epiblast cells	0.0588
7	Primary	Meningeal cells	0.0556

Figure S15: Clustering Performance of tsImpute on the Human Brain Dataset and Cell Type Annotation using DEGs and Canonical Markers. (a) Top 10 DEGs identified for each cluster by tsImpute. (b) Across-cluster average expression of cluster-specific DEGs. Violin plots show the distribution of average expression levels of these DEGs across all clusters. Strong clustering performance is indicated by high expression of cluster-specific DEGs within their respective clusters and low expression in other clusters. (c) Cell type annotation using the PanglaoDB marker database. Cell types are determined by the overlap between each cluster's DEGs and cell type-specific markers in the PanglaoDB database, with match scores computed based on the proportion of matched markers.

(a) Top 10 DE Genes for Each Cluster Identified by CAKE in Human Brain Data

Cluster 0	Cluster 1	Cluster 2	Cluster 3	Cluster 4	Cluster 5	Cluster 6
HMGB2	SELM	LUM	C11orf31	NEUROD6	FGF12	PAX6
CKB	HERPUD1	COL3A1	VIMP	STMN2	LMO3	C1orf61
CREB5	DCN	S100A11	UBE2C	MYT1L	ST18	PHLDA1
PAX6	LUM	DCN	MEIS1	RTN1	LMO7	CREB5
SLC1A3	VIMP	COL1A2	CCNB1	SOX4	MEF2C	SLC1A3
CCNB1	S100A11	MFAP4	RCHY1	NFIB	GRIA2	UBB
RNF157	IL11RA	POSTN	CDK1	GAP43	GDAP1	DOK5
UBE2C	COL1A2	LGALS1	MBD1	NFIA	GRIK2	CKB
DOK5	COL3A1	SELM	SELT	STMN1	SCG3	NFIA
IFI44L	PTPRM	COL21A1	NME6	BCL11B	ELMOD1	MEIS2

(b) Average Expression of Top 10 DE Genes from Each Cluster Found by CAKE

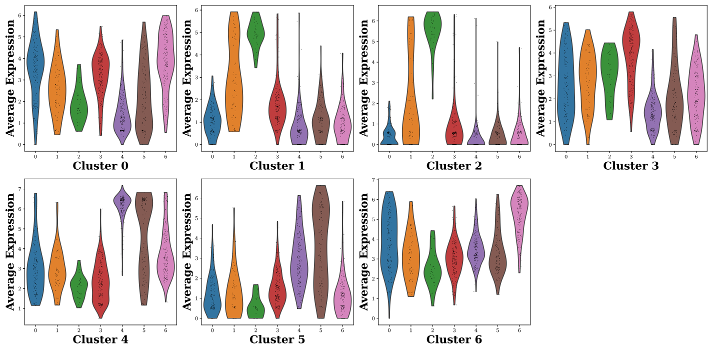

(c) Top Cell Type Matches for Each Cluster Identified by CAKE Based on PanglaoDB Markers

Cluster	Match Type	Annotation	Score
0	Primary	Radial glia cells	0.1429
	Secondary	Retinal progenitor cells	0.0769
1	Primary	Meningeal cells	0.1111
	Secondary	Pancreatic stellate cells	0.0690
2	Primary	Meningeal cells	0.1111
	Secondary	Pancreatic stellate cells	0.0690
3	Primary	No annotation	–
4	Primary	Nuocytes	0.0833
5	Primary	No annotation	–
6	Primary	Radial glia cells	0.1429
	Secondary	Glutaminergic neurons	0.1000

Figure S16: Clustering Performance of CAKE on the Human Brain Dataset and Cell Type Annotation using DEGs and Canonical Markers. (a) Top 10 DEGs identified for each cluster by CAKE. (b) Across-cluster average expression of cluster-specific DEGs. Violin plots show the distribution of average expression levels of these DEGs across all clusters. Strong clustering performance is indicated by high expression of cluster-specific DEGs within their respective clusters and low expression in other clusters. (c) Cell type annotation using the PanglaoDB marker database. Cell types are determined by the overlap between each cluster's DEGs and cell type-specific markers in the PanglaoDB database, with match scores computed based on the proportion of matched markers.

(a) Top 10 DE Genes for Each Cluster Identified by scASDC in Human Brain Data

Cluster 0	Cluster 1	Cluster 2	Cluster 3	Cluster 4	Cluster 5	Cluster 6
C1orf61	GAP43	LUM	CAMKV	UBE2C	PABPC4	EIF2B4
CKB	MYT1L	COL3A1	SYN3	CDK1	UBB	BBS2
PAX6	RTN1	DCN	NOL4	CCNB1	AKAP9	SLC1A3
HMGB2	GRIA2	LGALS1	FGF12	TOP2A	PLXNA2	BSCL2
CREB5	STMN2	MFAP4	CAMTA2	HMGB2	MFAP4	PPFIA2
SLC1A3	NRXN1	CACYBP	SNAP91	KNTC1	COL3A1	ZSCAN26
SPAG9	NEUROD6	KBTBD2	HMP19	UCP2	HERPUD1	CTTNBP2
PHLDA1	GPM6A	S100A11	SCN2A	C11orf31	LUM	MIOS
UBB	KIDINS220	MAGED2	PHACTR3	TXNRD1	DCN	TTLL7
MEIS2	RUNX1T1	FLRT3	CALB2	ACTL6A	S100A11	ALPK1

(b) Average Expression of Top 10 DE Genes from Each Cluster Found by scASDC

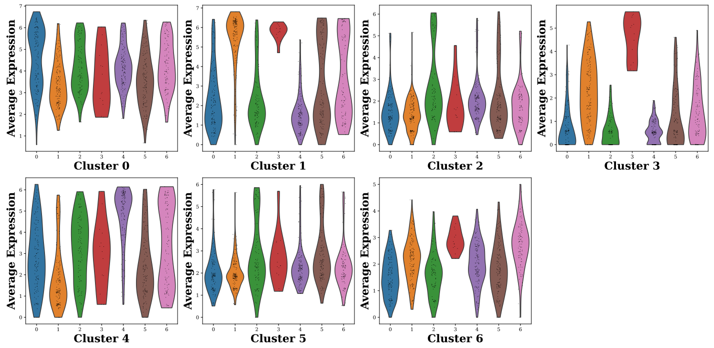

(c) Top Cell Type Matches for Each Cluster Identified by scASDC Based on PanglaoDB Markers

Cluster	Match Type	Annotation	Score
0	Primary	Radial glia cells	0.1429
	Secondary	Glutaminergic neurons	0.1000
1	Primary	No annotation	–
2	Primary	Meningeal cells	0.1111
3	Primary	Retinal progenitor cells	0.0769
4	Primary	Gamma delta T cells	0.0606
5	Primary	Meningeal cells	0.1111
6	Primary	Radial glia cells	0.0714

Figure S17: Clustering Performance of scASDC on the Human Brain Dataset and Cell Type Annotation using DEGs and Canonical Markers. (a) Top 10 DEGs identified for each cluster by scASDC. (b) Across-cluster average expression of cluster-specific DEGs. Violin plots show the distribution of average expression levels of these DEGs across all clusters. Strong clustering performance is indicated by high expression of cluster-specific DEGs within their respective clusters and low expression in other clusters. (c) Cell type annotation using the PanglaoDB marker database. Cell types are determined by the overlap between each cluster's DEGs and cell type-specific markers in the PanglaoDB database, with match scores computed based on the proportion of matched markers.

(a) Top 10 DE Genes for Each Cluster Identified by CoMem-DIPHW in Mouse Pancreas Data

Cluster 0	Cluster 1	Cluster 2	Cluster 3	Cluster 4	Cluster 5	Cluster 6
Hspa1b	Sparc	B2m	Ttr	Pdyn	Pecam1	Krt8
Hspa1a	Serpinh1	Cd74	Gpx3	Hmox1	Egfl7	Krt18
Dnajb1	Cald1	Tmsb4x	Vgf	Vgf	Col4a2	Krt19
Hspb1	Msn	Coro1a	Irx1	Ubr4	Cdh5	Ahnak
Krt8	Col4a1	Ctss	Rbp4	Zranb2	Flt1	Anxa2
Krt18	Col4a2	H2-Eb1	Mafb	Psap	Sparc	Prdx1
Fth1	Vim	Cytip	Spp1	Nr4a2	B2m	Anxa3
Fosb	Ier3	H2-Aa	Id3	Ftl1	Plvap	Ezr
Ddit4	Gm13889	Sh3bgrl3	Serping1	Ctsb	Ctla2a	S100a6
Gem	Anxa2	Srgn	Id1	Adgrl1	Col4a1	Clu

Cluster 7	Cluster 8	Cluster 9	Cluster 10	Cluster 11	Cluster 12
Rbp4	Ttr	Spp1	Hmox1	Hspa1b	Rbp4
Cd24a	Psap	Ttr	Ctsb	Hspa1a	Gpx3
Hhex	Pdyn	Cldn4	Krt18	Pdyn	Tspan8
Clu	Zranb2	Adgrl1	Psap	Dnajb1	Serping1
Arg1	Satb1	Gpx3	Krt8	Zranb2	Clu
Tspan8	Slc35b4	Hmox1	Ftl1	Ubr4	Arg1
Gpx3	Tnfrsf9	Slc35b4	Cldn4	Psap	Trp53i11
Mest	Vgf	Satb1	Ctrb1	Rspo4	Cldn4
Igfbp7	Sbk1	Lrig2	Rspo4	Th	Krt18
Cpa2	Them4	Trim9	Derl3	Adgrl1	Pigr

(c) Top Cell Type Matches for Each Cluster Identified by CoMem-DIPHW Based on PanglaoDB Markers

Cluster	Match Type	Annotation	Score
0	Primary	Merkel cells	0.1333
	Secondary	Hepatoblasts	0.1176
1	Primary	Myofibroblasts	0.1111
	Secondary	Glomus cells	0.1071
2	Primary	No annotation	-
3	Primary	Alpha cells	0.0652
	Secondary	Ductal cells	0.0500
4	Primary	Red pulp macrophages	0.0833
	Secondary	Dopaminergic neurons	0.0500
5	Primary	Pancreatic stellate cells	0.0690
	Secondary	Stromal cells	0.0606
6	Primary	Merkel cells	0.1333
	Secondary	Hepatoblasts	0.1176
7	Primary	Nuocytes	0.0833
	Secondary	Delta cells	0.0625
8	Primary	No annotation	-
9	Primary	Red pulp macrophages	0.0833
	Secondary	Ductal cells	0.0500
10	Primary	Merkel cells	0.1333
	Secondary	Hepatoblasts	0.1176
11	Primary	Juxtaglomerular cells	0.1429
	Secondary	Adrenergic neurons	0.1250
12	Primary	Ductal cells	0.1000
	Secondary	Nuocytes	0.0833

(b) Average Expression of Top 10 DE Genes from Each Cluster Found by CoMem-DIPHW

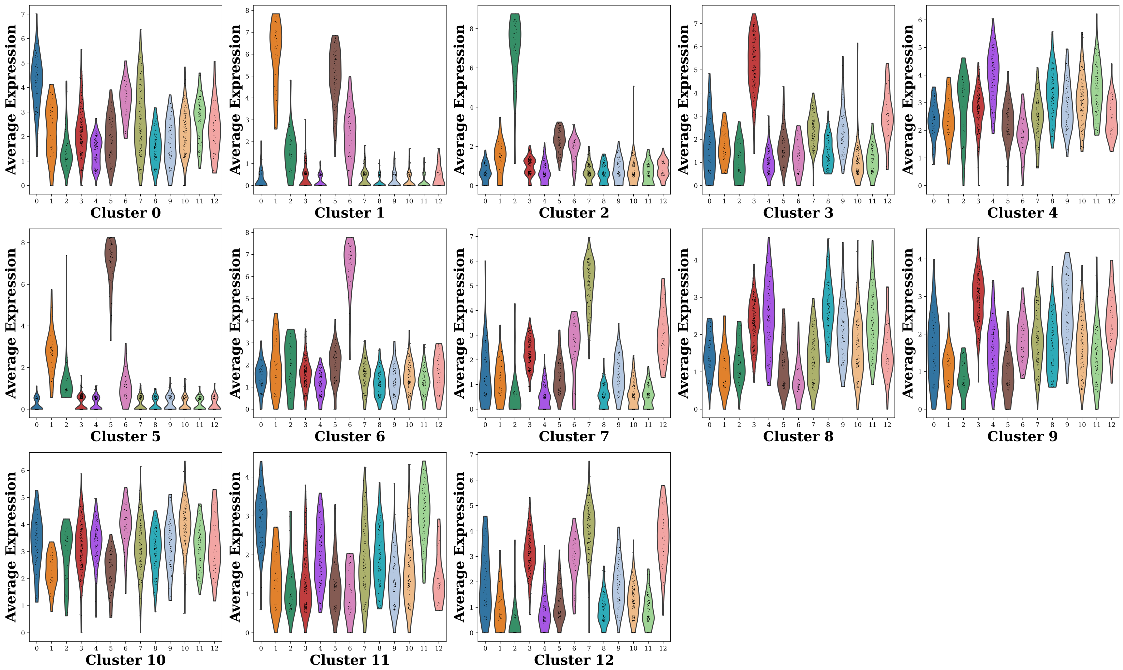

Figure S18: Clustering Performance of CoMem-DIPHW on the Mouse Pancreas Dataset and Cell Type Annotation using DEGs and Canonical Markers. (a) Top 10 DEGs identified for each cluster by CoMem-DIPHW. (b) Across-cluster average expression of cluster-specific DEGs. Violin plots show the distribution of average expression levels of these DEGs across all clusters. Strong clustering performance is indicated by high expression of cluster-specific DEGs within their respective clusters and low expression in other clusters. (c) Cell type annotation using the PanglaoDB marker database. Cell types are determined by the overlap between each cluster's DEGs and cell type-specific markers in the PanglaoDB database, with match scores computed based on the proportion of matched markers.

(a) Top 10 DE Genes for Each Cluster Identified by PCA in Mouse Pancreas Data

Cluster 0	Cluster 1	Cluster 2	Cluster 3	Cluster 4	Cluster 5	Cluster 6
Hmox1	Sparc	Ttr	Krt8	Rbp4	Hmox1	Pecam1
Coro1a	Serpinh1	Gpx3	Clu	Cd24a	Zranb2	Egfl7
Cytip	Cald1	Vgf	Krt18	Hhex	Vgf	Cdh5
Cd74	Msn	Mafb	Krt19	Clu	Pdyn	Col4a2
Ptprc	Vim	Rbp4	S100a6	Arg1	Ftl1	Flt1
Ctrb1	Col4a1	Irx1	Anxa2	Tspan8	Actn1	Sparc
Rac2	Col4a2	Id1	Anxa3	Gpx3	Rspo4	Plvap
Cela1	Smtn	Spp1	Prdx1	Mest	Ctsb	B2m
Stk17b	Csrp1	Tmsb4x	Ahnak	Igfbp7	Nr4a2	Ctla2a
Cd52	Anxa2	Id3	Nfib	Cpa2	Il6st	Msn

Cluster 7	Cluster 8	Cluster 9	Cluster 10	Cluster 11	Cluster 12
H2-Eb1	Ttr	Cldn4	Hmox1	Hspa1b	Gpx3
Pla2g7	Pdyn	Adgrl1	Pdyn	Hspa1a	Spp1
Psap	Vgf	Psap	Zranb2	Dnajb1	Tspan8
Ctss	Amy1	Krt8	Gclc	Krt8	Ttr
Fth1	Zranb2	Slc35b4	Ftl1	Rspo4	Rbp4
H2-Aa	Satb1	Krt18	Psap	Krt18	Hspb1
Ftl1	Psap	Th	Ctsb	Psap	Hspa1b
Cd74	Ubr4	Sptbn4	Pde4b	Nr4a1	Krt18
H2-Ab1	Slc39a14	Lyve1	Slc35b4	Ddit4	Serping1
Apoe	Il6st	Msln	Rspo4	Derl3	Clu

(c) Top Cell Type Matches for Each Cluster Identified by PCA Based on PanglaoDB Markers

Cluster	Match Type	Annotation	Score
0	Primary	Red pulp macrophages	0.0833
	Secondary	Langerhans cells	0.0667
1	Primary	Myofibroblasts	0.1111
	Secondary	Glomus cells	0.1071
2	Primary	Alpha cells	0.0652
3	Primary	Merkel cells	0.1333
	Secondary	Hepatoblasts	0.1176
4	Primary	Nuocytes	0.0833
	Secondary	Delta cells	0.0625
5	Primary	Red pulp macrophages	0.0833
	Secondary	Dopaminergic neurons	0.0500
6	Primary	No annotation	-
7	Primary	No annotation	-
8	Primary	No annotation	-
9	Primary	Juxtaglomerular cells	0.1429
	Secondary	Merkel cells	0.1333
10	Primary	Red pulp macrophages	0.0833
11	Primary	Merkel cells	0.1333
	Secondary	Hepatoblasts	0.1176
12	Primary	Ductal cells	0.0750
	Secondary	Merkel cells	0.0667

(b) Average Expression of Top 10 DE Genes from Each Cluster Found by PCA

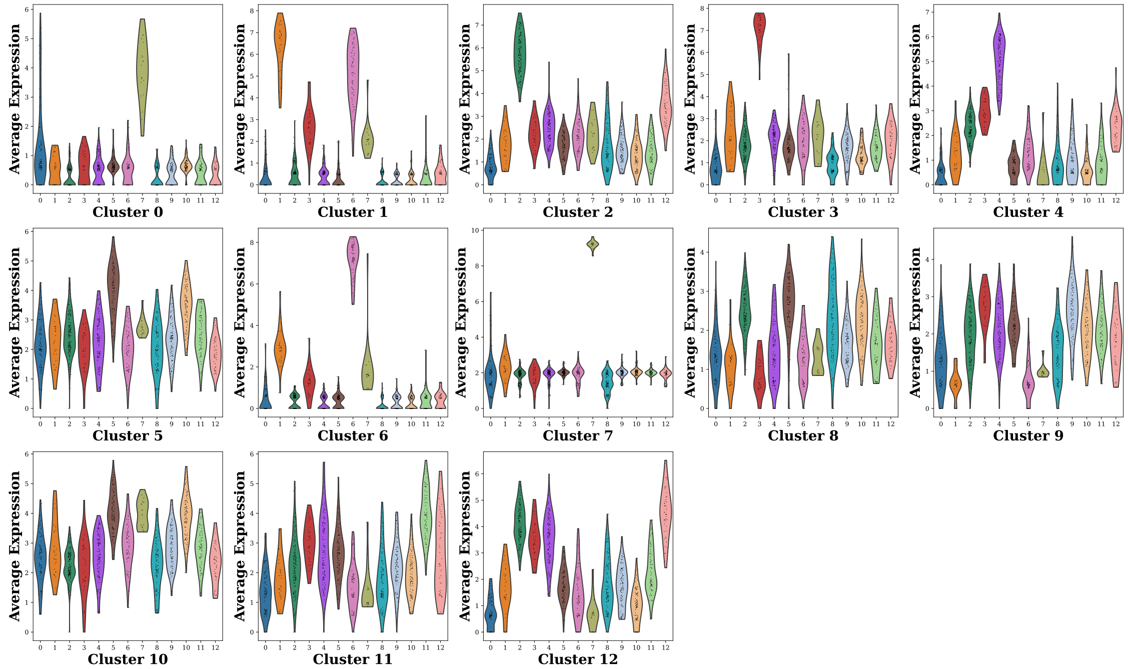

Figure S19: Clustering Performance of PCA on the Mouse Pancreas Dataset and Cell Type Annotation using DEGs and Canonical Markers. (a) Top 10 DEGs identified for each cluster by PCA. (b) Across-cluster average expression of cluster-specific DEGs. Violin plots show the distribution of average expression levels of these DEGs across all clusters. Strong clustering performance is indicated by high expression of cluster-specific DEGs within their respective clusters and low expression in other clusters. (c) Cell type annotation using the PanglaoDB marker database. Cell types are determined by the overlap between each cluster's DEGs and cell type-specific markers in the PanglaoDB database, with match scores computed based on the proportion of matched markers.

(a) Top 10 DE Genes for Each Cluster Identified by graph-sc in Mouse Pancreas Data

Cluster 0	Cluster 1	Cluster 2	Cluster 3	Cluster 4	Cluster 5
Pecam1	Rbp4	Cd52	Prdx1	Smox	Hmox1
Egfl7	Gpx3	Fermt3	Flna	Ramp3	Pdyn
Flt1	Serping1	Rac2	Anxa2	Zfp36l1	Psap
Cdh5	Clu	Coro1a	Msn	Srgn	Rspo4
Sparc	Arg1	Arhgdib	Fth1	Pde4b	Vgf
Col4a2	Spp1	Dock2	Tuba1c	Tmsb4x	Zranb2
B2m	Ttr	Il2rg	Tmsb4x	B2m	Ubr4
Plvap	Tspan8	Itgb2	Cd44	Traf1	Slc35b4
Ctla2a	Irx1	Myo1g	B2m	Lsp1	Satb1
Sox4	Basp1	Ptprc	Cd14	Rgs1	Ctsb
**Cluster 6**	**Cluster 7**	**Cluster 8**	**Cluster 9**	**Cluster 11**	**Cluster 12**
Tgfbi	S100a6	Ighm	Cytip	Rbp4	Anxa2
Fcer1g	Anxa2	Coro1a	Marcksl1	Hhex	Csrp1
Lyz2	Csrp1	Cd74	Cd80	Cd24a	Tuba1c
H2-Eb1	Myof	B2m	Tbc1d4	Clu	Actn1
Pla2g7	Cd14	Pou2af1	Lsp1	Gpx3	Tmsb4x
Mpeg1	Cyba	Iglc3	Tmsb4x	Arg1	Msn
H2-Ab1	Krt19	Cd79a	Tmem123	Mest	Flna
Fth1	Onecut2	Igkc	Gm13546	Gap43	Rhoc
Laptm5	Fth1	Rac2	Ccl22	Peg10	Ahnak
Ctss	Tspan8	Cd52	Ccr7	Tspan8	Cald1

(c) Top Cell Type Matches for Each Cluster Identified by graph-sc Based on PanglaoDB Markers

Cluster	Match Type	Annotation	Score
0	Primary	No annotation	-
1	Primary	Nuocytes	0.0833
	Secondary	Ductal cells	0.0750
2	Primary	Langerhans cells	0.0667
	Secondary	Tuft cells	0.0526
3	Primary	No annotation	-
4	Primary	No annotation	-
5	Primary	Red pulp macrophages	0.0833
6	Primary	No annotation	-
7	Primary	Microfold cells	0.0588
	Secondary	Ductal cells	0.0500
8	Primary	B cells naive	0.0893
	Secondary	Plasma cells	0.0746
9	Primary	Myeloid-derived suppressor cells	0.0833
	Secondary	Langerhans cells	0.0667
11	Primary	Nuocytes	0.0833
	Secondary	Delta cells	0.0625
12	Primary	Myofibroblasts	0.1111
	Secondary	Mesangial cells	0.0526

(b) Average Expression of Top 10 DE Genes from Each Cluster Found by graph-sc

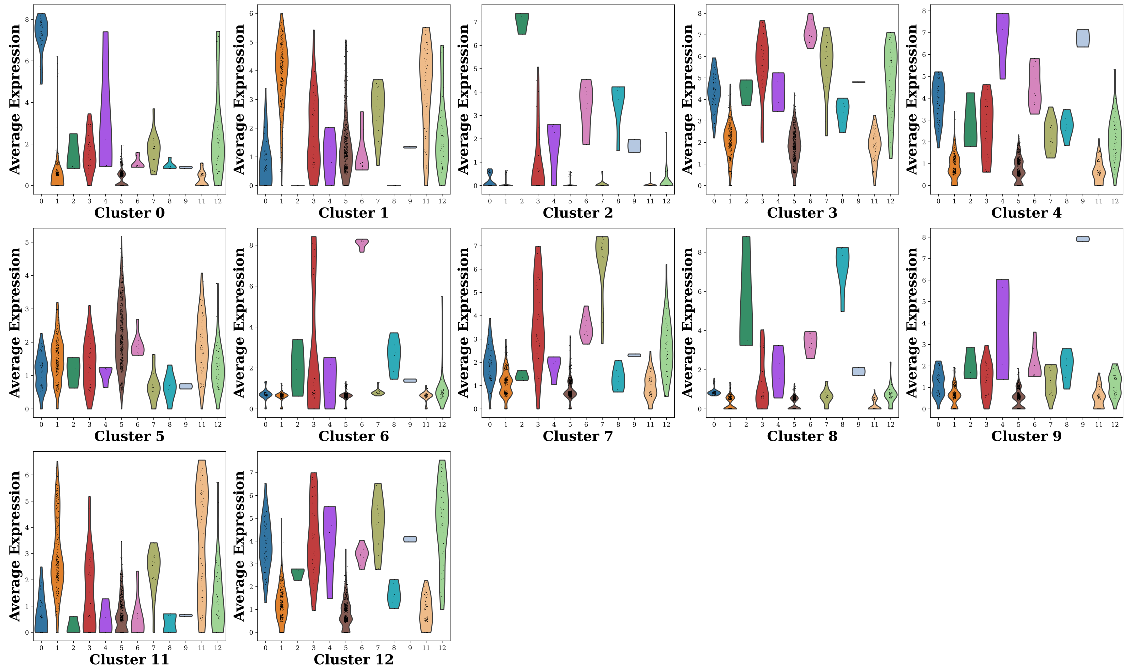

Figure S20: Clustering Performance of graph-sc on the Mouse Pancreas Dataset and Cell Type Annotation using DEGs and Canonical Markers. (a) Top 10 DEGs identified for each cluster by graph-sc. (b) Across-cluster average expression of cluster-specific DEGs. Violin plots show the distribution of average expression levels of these DEGs across all clusters. Strong clustering performance is indicated by high expression of cluster-specific DEGs within their respective clusters and low expression in other clusters. (c) Cell type annotation using the PanglaoDB marker database. Cell types are determined by the overlap between each cluster's DEGs and cell type-specific markers in the PanglaoDB database, with match scores computed based on the proportion of matched markers.

(a) Top 10 DE Genes for Each Cluster Identified by TsImpute in Mouse Pancreas Data

Cluster 1	Cluster 2	Cluster 3	Cluster 4	Cluster 5	Cluster 6
Anxa2	Rbp4	Prdx1	Pecam1	Gpx3	Gpx3
Tmsb4x	Gpx3	Cyba	Mmrn2	Rbp4	Rbp4
B2m	Cldn4	Tmsb4x	Egfl7	Serping1	Spp1
Msn	Clu	Fth1	Esam	Spp1	Clu
Sox4	Krt18	Ftl1	Adgrl4	Tspan8	Serping1
Ier3	Arg1	B2m	Cdh5	Arg1	Arg1
Sparc	Krt8	Flna	Dll4	Vgf	Ttr
Flna	Spp1	Lgals3	Flt1	Igfbp7	Dnajb1
Ifitm3	Hhex	Cd44	Plvap	Ttr	Irx1
Col4a1	Cd24a	Smox	S1pr1	Fgl2	Peg10

Cluster 7	Cluster 9	Cluster 10	Cluster 11	Cluster 12	Cluster 13
Vgf	Psap	Gpx3	Hmox1	Msn	Spp1
Hmox1	Pdyn	Rbp4	Rspo4	B2m	Gpx3
Nr4a2	Ctrb1	Ttr	Psap	Col4a2	Tspan8
Zranb2	Msln	Mafb	Ftl1	Sparc	Rbp4
Pdyn	Amy1	Serping1	Pdyn	Col4a1	Clu
Ctsb	Derl3	Krt8	Adgrl1	Anxa2	Ttr
Ftl1	Th	Vgf	Zranb2	Tmsb4x	Fosb
Gsto1	Ubr4	Irx1	Ctsb	Ier3	Serping1
Slc35b4	Hspa1b	Basp1	Slc35b4	Pmepa1	Fabp5
Rspo4	Cd79a	Krt18	Npat	Ednrb	Actn1

(c) Top Cell Type Matches for Each Cluster Identified by TsImpute Based on PanglaoDB Markers

Cluster	Match Type	Annotation	Score
1	Primary	Pancreatic stellate cells	0.0690
2	Primary	Hepatoblasts	0.1765
	Secondary	Merkel cells	0.1333
3	Primary	No annotation	-
4	Primary	Stromal cells	0.0606
5	Primary	Nuocytes	0.0833
	Secondary	Ductal cells	0.0750
6	Primary	Nuocytes	0.0833
	Secondary	Ductal cells	0.0500
7	Primary	Red pulp macrophages	0.0833
	Secondary	Dopaminergic neurons	0.0500
9	Primary	Juxtaglomerular cells	0.1429
	Secondary	Adrenergic neurons	0.1250
10	Primary	Merkel cells	0.0667
	Secondary	Alpha cells	0.0652
11	Primary	Red pulp macrophages	0.0833
12	Primary	Pancreatic stellate cells	0.0690
13	Primary	Ductal cells	0.0750

(b) Average Expression of Top 10 DE Genes from Each Cluster Found by TsImpute

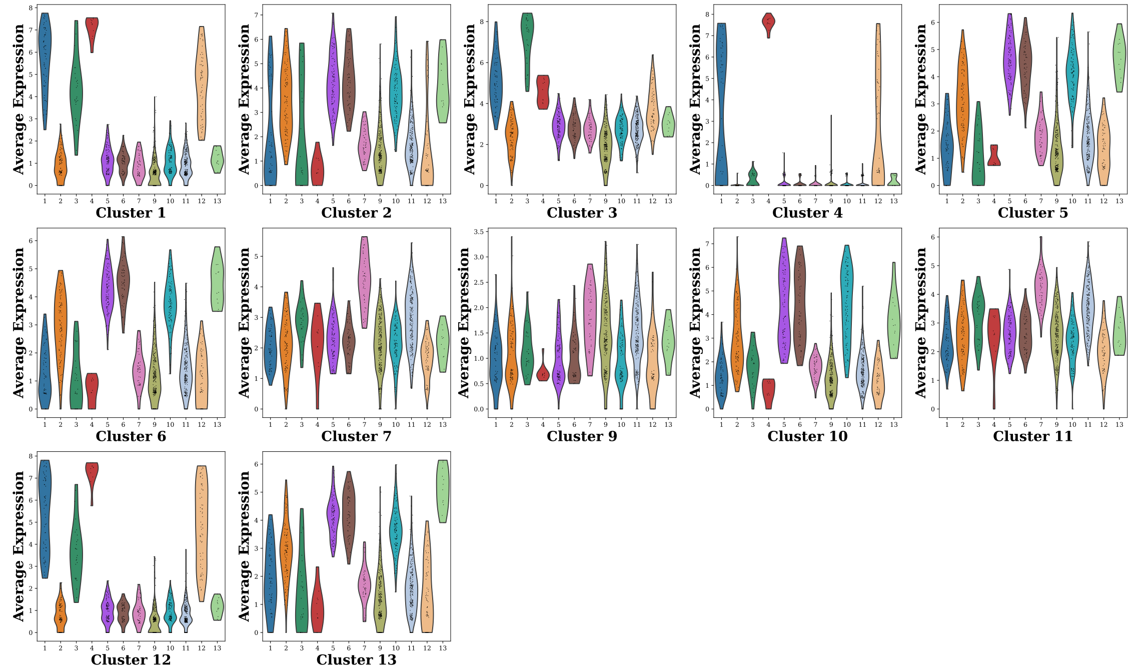

Figure S21: Clustering Performance of tsImpute on the Mouse Pancreas Dataset and Cell Type Annotation using DEGs and Canonical Markers. (a) Top 10 DEGs identified for each cluster by tsImpute. (b) Across-cluster average expression of cluster-specific DEGs. Violin plots show the distribution of average expression levels of these DEGs across all clusters. Strong clustering performance is indicated by high expression of cluster-specific DEGs within their respective clusters and low expression in other clusters. (c) Cell type annotation using the PanglaoDB marker database. Cell types are determined by the overlap between each cluster's DEGs and cell type-specific markers in the PanglaoDB database, with match scores computed based on the proportion of matched markers.

(a) Top 10 DE Genes for Each Cluster Identified by CAKE in Mouse Pancreas Data

Cluster 0	Cluster 1	Cluster 2	Cluster 3	Cluster 4	Cluster 5	Cluster 6
Hmox1	Rbp4	Pdyn	Pecam1	Krt19	B2m	Gpx3
Ezr	Gpx3	Hspa1b	Egfl7	Clu	Cd74	Ttr
Pdyn	Hhex	Rspo4	Cdh5	Krt8	Tmsb4x	Mafb
Psap	Arg1	Zranb2	Col4a2	Anxa2	Cytip	Spp1
Ddit4	Cd24a	Hspa1a	Sparc	Myof	Coro1a	Irx1
Tnfrsf9	Clu	Nr4a2	Flt1	Krt18	Srgn	Id1
Stk4	Tspan8	Vgf	B2m	S100a6	Cyba	Vgf
Th	Mest	Slc35b4	Plvap	Ahnak	Ctss	Rbp4
Il6st	Serping1	Adgrl1	Ctla2a	Muc1	Laptm5	Id3
Gsto1	Cpa2	Hmox1	Msn	Anxa3	H2-Eb1	Basp1
**Cluster 7**	**Cluster 8**	**Cluster 9**	**Cluster 10**	**Cluster 11**	**Cluster 12**	
Rbp4	Psap	Anxa2	Gpx3	Gm13889	Gpx3	
Hhex	Krt18	Cald1	Ttr	Ednrb	Rbp4	
Cd24a	Rspo4	Fth1	Rbp4	Igfbp5	Serping1	
Arg1	Msln	Flna	Irx1	Smtn	Cldn4	
Clu	Nr4a1	Sparc	Mafb	Sparc	Tspan8	
Tspan8	Cela2a	Ier3	Spp1	Msn	Cldn3	
Igfbp7	Ctrb1	Msn	Vgf	S1pr3	Krt18	
Gpx3	Hspa1b	Csrp1	Id3	Rgs5	Ttr	
Mest	Ttr	Cd44	Basp1	Ndufa4l2	Krt8	
Gap43	Gstm2	Igfbp7	Serping1	Col4a1	Tmsb4x	

(c) Top Cell Type Matches for Each Cluster Identified by CAKE Based on PanglaoDB Markers

Cluster	Match Type	Annotation	Score
0	Primary	Juxtaglomerular cells	0.1429
	Secondary	Adrenergic neurons	0.1250
1	Primary	Nuocytes	0.0833
	Secondary	Ductal cells	0.0750
2	Primary	Dopaminergic neurons	0.0500
3	Primary	No annotation	-
4	Primary	Merkel cells	0.1333
	Secondary	Hepatoblasts	0.1176
5	Primary	No annotation	-
6	Primary	Alpha cells	0.0652
7	Primary	Nuocytes	0.0833
	Secondary	Delta cells	0.0625
8	Primary	Merkel cells	0.0667
	Secondary	Hepatoblasts	0.0588
9	Primary	Myofibroblasts	0.1111
10	Primary	Alpha cells	0.0652
11	Primary	Pancreatic stellate cells	0.1379
	Secondary	Glomus cells	0.0714
12	Primary	Merkel cells	0.1333
	Secondary	Hepatoblasts	0.1176

(b) Average Expression of Top 10 DE Genes from Each Cluster Found by CAKE

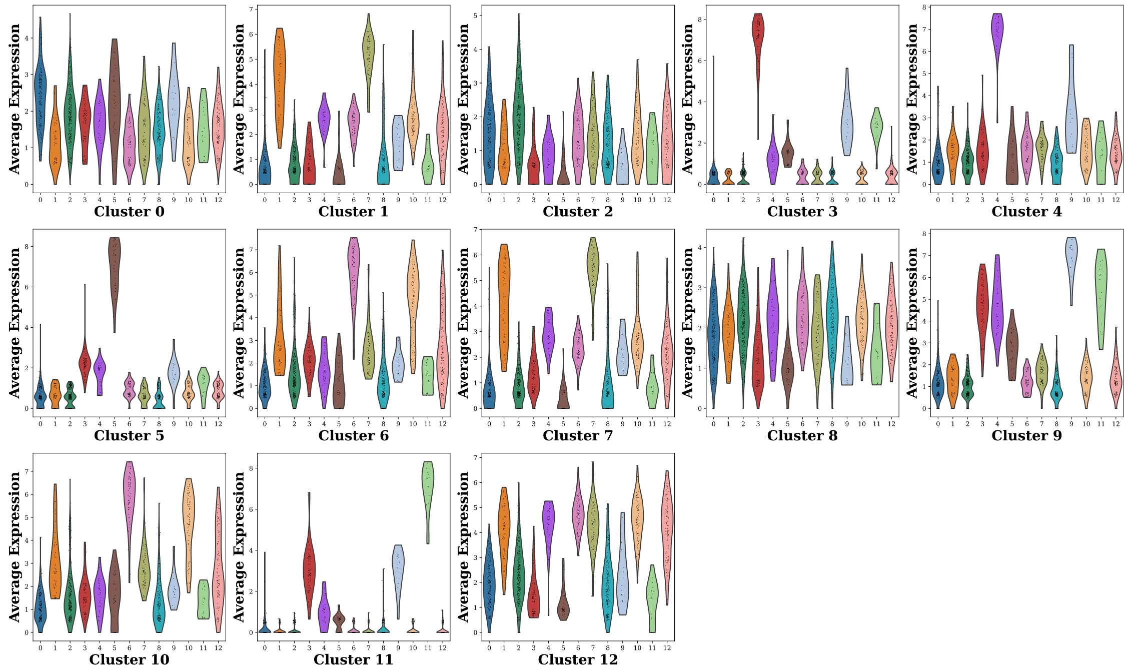

Figure S22: Clustering Performance of CAKE on the Mouse Pancreas Dataset and Cell Type Annotation using DEGs and Canonical Markers. (a) Top 10 DEGs identified for each cluster by CAKE. (b) Across-cluster average expression of cluster-specific DEGs. Violin plots show the distribution of average expression levels of these DEGs across all clusters. Strong clustering performance is indicated by high expression of cluster-specific DEGs within their respective clusters and low expression in other clusters. (c) Cell type annotation using the PanglaoDB marker database. Cell types are determined by the overlap between each cluster's DEGs and cell type-specific markers in the PanglaoDB database, with match scores computed based on the proportion of matched markers.

(a) Top 10 DE Genes for Each Cluster Identified by scASDC in Mouse Pancreas Data

Cluster 0	Cluster 1	Cluster 2	Cluster 3	Cluster 4	Cluster 5	Cluster 6
Pdyn	Mpeg1	Sparc	Rbp4	Ttr	Anxa2	Adgrl1
Hmox1	Ctss	Sox4	Cd24a	Vgf	Flna	Psap
Rspo4	Fth1	B2m	Hhex	Gpx3	Ier3	Pdyn
Psap	Psap	Col4a2	Arg1	Irx1	Serpinh1	Trim9
Zranb2	Fcer1g	Pecam1	Clu	Tmsb4x	Csrp1	Ctrb1
Satb1	Cd74	Egfl7	Igfbp7	Spp1	Col4a1	Zranb2
Actn1	H2-Eb1	Msn	Gpx3	Rbp4	Zfp36l1	Lrig2
Slc35b4	Laptm5	Flt1	Mest	Id3	Msn	Fam171a1
Krt8	Ftl1	Cdh5	Gap43	Fev	Sparc	Phlda1
Ftl1	H2-Aa	Rhoc	Tspan8	Nr4a2	Fth1	Krt18
**Cluster 7**	**Cluster 8**	**Cluster 9**	**Cluster 10**	**Cluster 11**	**Cluster 12**	
Hspa1b	Vgf	Gpx3	Hmox1	Ttr	B2m	
Hspa1a	Ttr	Rbp4	Vgf	Spp1	Tmsb4x	
Dnajb1	Krt8	Ttr	Prdx1	Abhd18	Coro1a	
Hmox1	Fgl2	Serping1	Adgrl1	Mafb	Fth1	
Psap	Zranb2	Irx1	Fgl2	Fgl2	Cd74	
Rspo4	Ctsb	Clu	Tmem123	Gpx3	Cytip	
Pdyn	Gpx3	Spp1	Slc35b4	Serping1	Tmem123	
Ddit4	Cd68	Mafb	Ctsb	Irx1	Ctss	
Msln	Cldn4	Krt8	Psap	Actn1	H2-Eb1	
Ubr4	Ftl1	Krt18	Zranb2	Vgf	H2-Aa	

(c) Top Cell Type Matches for Each Cluster Identified by scASDC Based on PanglaoDB Markers

Cluster	Match Type	Annotation	Score
0	Primary	Red pulp macrophages	0.0833
1	Primary	No annotation	-
2	Primary	No annotation	-
3	Primary	Nuocytes	0.0833
	Secondary	Delta cells	0.0625
4	Primary	Serotonergic neurons	0.1250
	Secondary	Alpha cells	0.0652
5	Primary	Pancreatic stellate cells	0.0690
6	Primary	Merkel cells	0.0667
	Secondary	Hepatoblasts	0.0588
7	Primary	Red pulp macrophages	0.0833
8	Primary	Merkel cells	0.0667
	Secondary	Hepatoblasts	0.0588
9	Primary	Merkel cells	0.1333
	Secondary	Hepatoblasts	0.1176
10	Primary	Red pulp macrophages	0.0833
11	Primary	Alpha cells	0.0652
	Secondary	Ductal cells	0.0500
12	Primary	No annotation	-

(b) Average Expression of Top 10 DE Genes from Each Cluster Found by scASDC

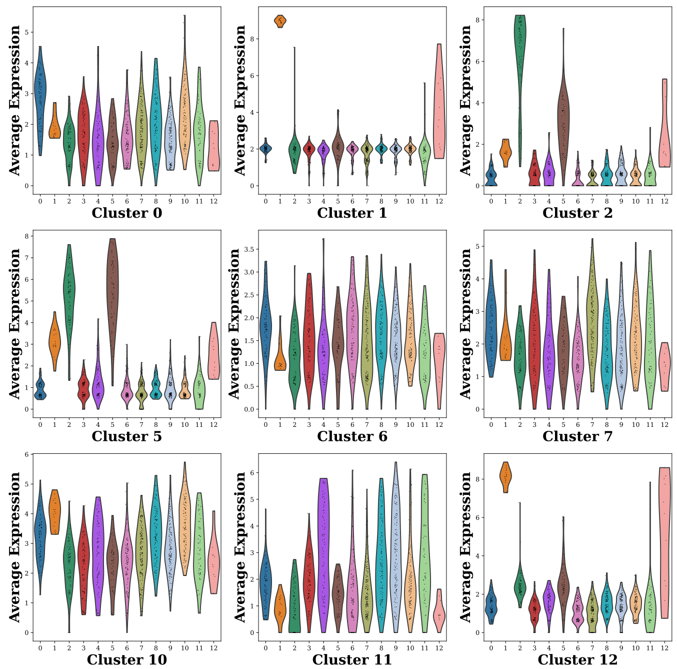

Figure S23: Clustering Performance of scASDC on the Mouse Pancreas Dataset and Cell Type Annotation using DEGs and Canonical Markers. (a) Top 10 DEGs identified for each cluster by scASDC. (b) Across-cluster average expression of cluster-specific DEGs. Violin plots show the distribution of average expression levels of these DEGs across all clusters. Strong clustering performance is indicated by high expression of cluster-specific DEGs within their respective clusters and low expression in other clusters. (c) Cell type annotation using the PanglaoDB marker database. Cell types are determined by the overlap between each cluster's DEGs and cell type-specific markers in the PanglaoDB database, with match scores computed based on the proportion of matched markers.

(a) Top 10 DE Genes for Each Cluster Identified by CoMem-DIPHW in Mouse Brain Data

Cluster 0	Cluster 1	Cluster 2	Cluster 3	Cluster 4
Pcp4	Mag	Sparc	Gad1	Csf1r
Slc1a2	Gjc3	Epas1	Gad2	Ctss
Lamp5	Ptgds	Esam	Vstm2a	Tyrobp
Ier5	Nfasc	Itm2a	Slc6a1	C1qa
Myl4	Fa2h	Igfbp7	Dlx6os1	C1qb
Vstm2a	Hapln2	Hes1	Dner	Rnase4
Clu	S100b	Myl12a	Cnr1	Fcrls
Ablim1	Serpinb1a	Ly6c1	Igf1	Laptm5
Tnnc1	S100a6	Id3	Arl4c	Fcgr3
Rorb	S100a1	Cldn5	Pnoc	Sepp1
**Cluster 5**	**Cluster 6**	**Cluster 7**	**Cluster 8**	
Clu	Wfs1	Tmem212	Nnat	
Slc1a3	Rasd1	Ccdc153	Dcn	
Gja1	Tpm1	Cd24a	Resp18	
Ppap2b	Nr4a2	Rarres2	Nr4a2	
Slc4a4	Palmd	Dynlrb2	Cnr1	
Gpr37l1	Flna	Mia	Wfs1	
Atp1a2	Kctd12	Fam183b	Kctd12	
Ptprz1	Ncan	1110017D15Rik	Itga8	
Ntsr2	Dner	1700094D03Rik	Efnb2	
Aldoc	Itga8	Riiad1	Rgs2	

(c) Top Cell Type Matches for Each Cluster Identified by CoMem-DIPHW Based on PanglaoDB Markers

Cluster	Match Type	Annotation	Score
0	Primary	Glutaminergic neurons	0.1000
1	Primary	Nuocytes	0.0833
	Secondary	Enteric glia cells	0.0588
2	Primary	Retinal progenitor cells	0.0769
	Secondary	Pancreatic progenitor cells	0.0667
3	Primary	GABAergic neurons	0.2000
4	Primary	Kupffer cells	0.0652
	Secondary	Microglia	0.0633
5	Primary	Bergmann glia	0.1220
	Secondary	Astrocytes	0.0952
6	Primary	Peritubular myoid cells	0.0800
	Secondary	Mesangial cells	0.0702
7	Primary	Ciliated cells	0.1111
	Secondary	Ependymal cells	0.1017
8	Primary	Osteocytes	0.2000
	Secondary	Meningeal cells	0.1111

(b) Average Expression of Top 10 DE Genes from Each Cluster Found by CoMem-DIPHW

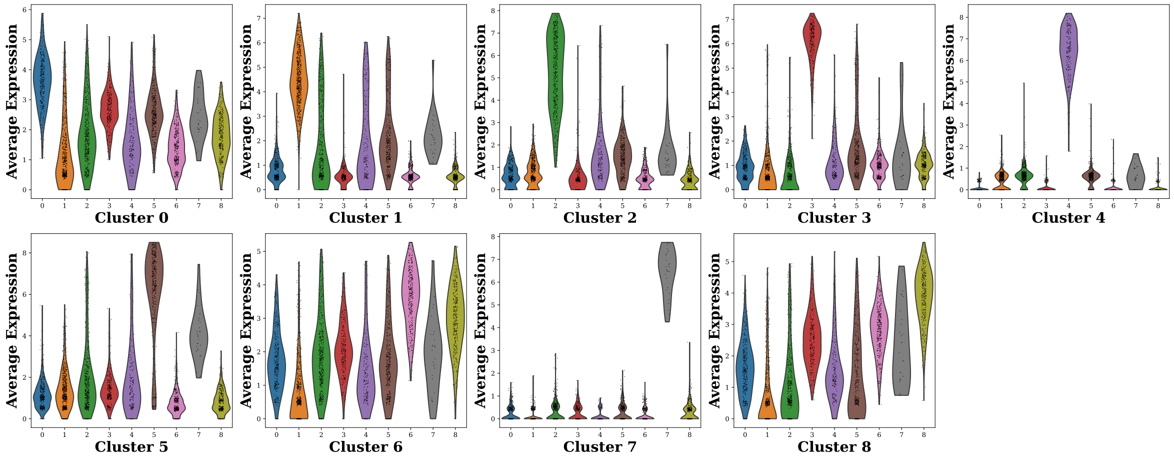

Figure S24: Clustering Performance of CoMem-DIPHW on the Mouse Brain Dataset and Cell Type Annotation using DEGs and Canonical Markers. (a) Top 10 DEGs identified for each cluster by CoMem-DIPHW. (b) Across-cluster average expression of cluster-specific DEGs. Violin plots show the distribution of average expression levels of these DEGs across all clusters. Strong clustering performance is indicated by high expression of cluster-specific DEGs within their respective clusters and low expression in other clusters. (c) Cell type annotation using the PanglaoDB marker database.

(a) Top 10 DE Genes for Each Cluster Identified by PCA in Mouse Brain Data

Cluster 0	Cluster 1	Cluster 2	Cluster 3	Cluster 4
Pcp4	Slc1a3	Itm2a	Mag	Wfs1
Slc1a2	Clu	Sparc	Gjc3	Nnat
Lamp5	Gja1	Ly6c1	Ptgds	Nr4a2
Ier5	Ppap2b	Cldn5	Nfasc	Dcn
Myl4	Slc1a2	Ptprb	Fa2h	Kctd12
Vstm2a	Aqp4	Slco1a4	Hapln2	Rasd1
Clu	Gpr37l1	Pltp	Serpinb1a	Cnr1
Rorb	Pla2g7	Esam	S100b	Tpm1
Tnnc1	Atp1a2	Flt1	Klk6	Itga8
Ablim1	Mt2	Abcb1a	S100a1	Palmd
**Cluster 5**	**Cluster 6**	**Cluster 7**	**Cluster 8**	
Gad1	Pf4	Hexb	Myl9	
Gad2	Mrc1	Ctss	Mgp	
Slc6a1	Tyrobp	P2ry12	Crip1	
Vstm2a	Lyz2	Cx3cr1	Acta2	
Dner	C1qa	Csf1r	Tagln	
Dlx6os1	Csf1r	Gpr34	Tpm1	
Igf1	Cbr2	Laptm5	Tpm2	
Arl4c	Stab1	Tyrobp	S100a11	
Resp18	Ms4a7	Rnase4	Myh11	
Cnr1	Sepp1	C1qb	Mustn1	

(c) Top Cell Type Matches for Each Cluster Identified by PCA Based on PanglaoDB Markers

Cluster	Match Type	Annotation	Score
0	Primary	Glutaminergic neurons	0.1000
1	Primary	Bergmann glia	0.1220
	Secondary	Glutaminergic neurons	0.1000
2	Primary	No annotation	–
3	Primary	Nuocytes	0.0833
	Secondary	Oligodendrocytes	0.0690
4	Primary	Meningeal cells	0.1111
	Secondary	Peritubular myoid cells	0.0800
5	Primary	GABAergic neurons	0.2000
6	Primary	No annotation	–
7	Primary	Microglia	0.0759
8	Primary	Myofibroblasts	0.3333
	Secondary	Vascular smooth muscle cells	0.3333

(b) Average Expression of Top 10 DE Genes from Each Cluster Found by PCA

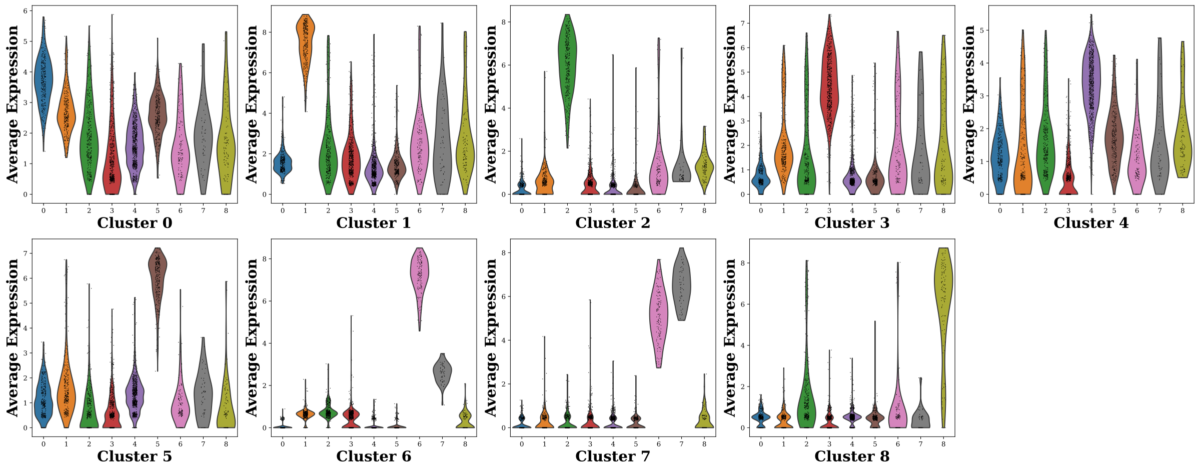

Figure S25: Clustering Performance of PCA on the Mouse Brain Dataset and Cell Type Annotation using DEGs and Canonical Markers. (a) Top 10 DEGs identified for each cluster by PCA. (b) Across-cluster average expression of cluster-specific DEGs. Violin plots show the distribution of average expression levels of these DEGs across all clusters. (c) Cell type annotation using the PanglaoDB marker database.

(a) Top 10 DE Genes for Each Cluster Identified by graph-sc in Mouse Brain Data

Cluster 0	Cluster 1	Cluster 2	Cluster 3	Cluster 4
Wfs1	Itm2a	Clu	Pf4	Mag
Nnat	Sparc	Gja1	Mrc1	Nfasc
Nr4a2	Ly6c1	Pla2g7	Lyz2	Tmem141
Tpm1	Ptprb	Gpr37l1	Csf1r	Ptgds
Rgs2	Cldn5	Slc1a3	Tyrobp	Gjc3
Ncan	Pltp	Mt1	Cbr2	Gpr17
Resp18	Slco1a4	Aqp4	C1qa	Abhd3
Dcn	Esam	Apoe	Stab1	Rnf122
Cnr1	Slc2a1	Mt2	Ccl7	Ppap2b
Ier5	Abcb1a	Ppap2b	F13a1	Fa2h
**Cluster 5**	**Cluster 6**	**Cluster 7**	**Cluster 8**	
Itm2a	Mag	Slc6a1	Ccl24	
Cldn5	Gjc3	Gad1	Ms4a7	
Ly6c1	Hapln2	Gad2	Mrc1	
Sparc	Serpinb1a	Clu	Lyz2	
Ptprb	Fa2h	Dner	Fcgr3	
Flt1	Ptgds	Vstm2a	Dab2	
Slco1a4	Klk6	Dlx6os1	Ctsc	
Egfl7	S100b	Cnr1	Tyrobp	
Esam	S100a6	Arl4c	Sepp1	
Slco1c1	S100a1	Igf1	Pf4	

(c) Top Cell Type Matches for Each Cluster Identified by graph-sc Based on PanglaoDB Markers

Cluster	Match Type	Annotation	Score
0	Primary	Meningeal cells	0.1111
	Secondary	Dopaminergic neurons	0.0500
1	Primary	No annotation	–
2	Primary	Bergmann glia	0.0976
	Secondary	Astrocytes	0.0952
3	Primary	No annotation	–
4	Primary	Nuocytes	0.0833
	Secondary	Meningeal cells	0.0556
5	Primary	No annotation	–
6	Primary	Nuocytes	0.0833
	Secondary	Oligodendrocytes	0.0690
7	Primary	GABAergic neurons	0.2000
8	Primary	No annotation	–

(b) Average Expression of Top 10 DE Genes from Each Cluster Found by graph-sc

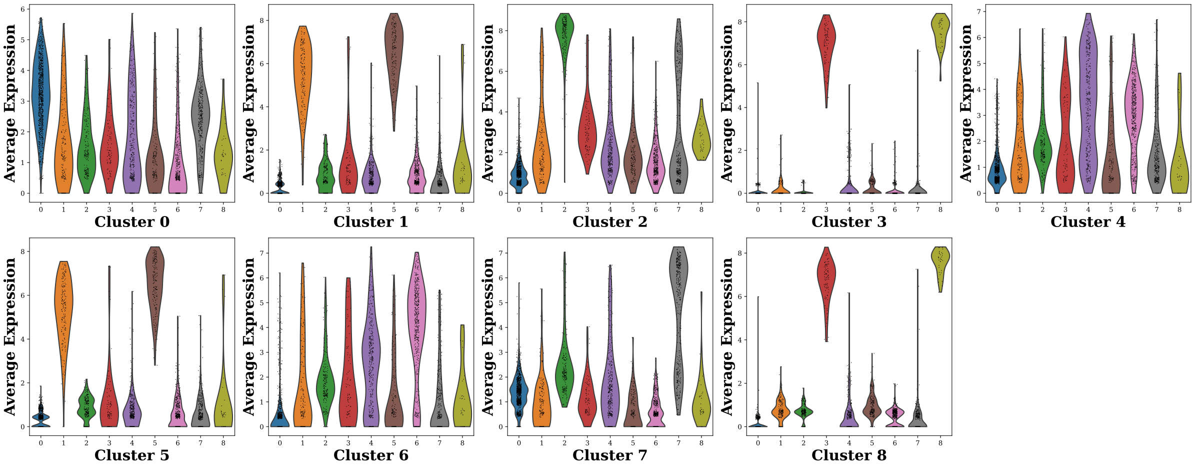

Figure S26: Clustering Performance of graph-sc on the Mouse Brain Dataset and Cell Type Annotation using DEGs and Canonical Markers. (a) Top 10 DEGs identified for each cluster by graph-sc. (b) Across-cluster average expression of cluster-specific DEGs. Violin plots show the distribution of average expression levels of these DEGs across all clusters. (c) Cell type annotation using the PanglaoDB marker database.

(a) Top 10 DE Genes for Each Cluster Identified by TsImpute in Mouse Brain Data

Cluster 1	Cluster 2	Cluster 3	Cluster 4	Cluster 5
Tmem141	Mag	Pla2g7	Sparc	Gad1
Mag	Gjc3	Dusp1	Sepp1	Gad2
Ptgds	Nfasc	Zfp36l1	Slco1c1	Igf1
Nfasc	Fa2h	Apoe	Hes1	Vstm2a
Prom1	Ptgds	Vamp8	Zfp36l1	Slc6a1
Gjc3	Hapln2	Abca1	Cyr61	Arl4c
Rnf122	Tmem141	Tmem176b	Id3	Dlx6os1
Fa2h	Prdx6	Cyr61	Atp1a2	Resp18
9630013A20Rik	Abhd3	Btg2	Epas1	Dner
Mfsd2a	S100b	Anxa5	Serpinh1	Maf
Cluster 6	Cluster 7	Cluster 8	Cluster 9	
Vstm2a	Apoe	Mag	Wfs1	
Dner	Sepp1	Gjc3	Nnat	
Resp18	Pla2g7	Ptgds	Nr4a2	
Cnr1	Mt1	Hapln2	Cnr1	
Gad2	Sat1	Fa2h	Resp18	
Arl4c	Tmem176b	Nfasc	Rgs2	
Gad1	Sdc4	Serpinb1a	Tpm1	
Dlx6os1	Ednrb	S100b	Ncan	
Col19a1	Zfp36l1	S100a1	Rasd1	
Igf1	Atp1a2	S100a6	Dcn	

(c) Top Cell Type Matches for Each Cluster Identified by TsImpute Based on PanglaoDB Markers

Cluster	Match Type	Annotation	Score
1	Primary	Nuocytes	0.0833
	Secondary	Crypt cells	0.0625
2	Primary	Nuocytes	0.0833
	Secondary	Enteric glia cells	0.0588
3	Primary	Spermatozoa	0.1111
4	Primary	Retinal progenitor cells	0.0769
	Secondary	Pancreatic progenitor cells	0.0667
5	Primary	GABAergic neurons	0.2000
6	Primary	GABAergic neurons	0.1333
7	Primary	No annotation	–
8	Primary	Nuocytes	0.0833
	Secondary	Enteric glia cells	0.0588
9	Primary	Meningeal cells	0.1111
	Secondary	Peritubular myoid cells	0.0800

(b) Average Expression of Top 10 DE Genes from Each Cluster Found by TsImpute

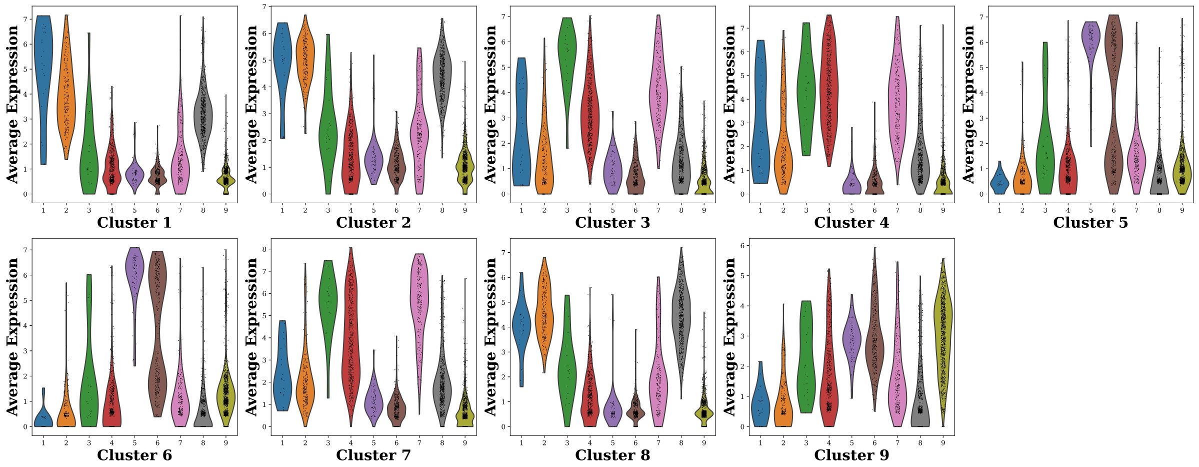

Figure S27: Clustering Performance of tsImpute on the Mouse Brain Dataset and Cell Type Annotation using DEGs and Canonical Markers. (a) Top 10 DEGs identified for each cluster by tsImpute. (b) Across-cluster average expression of cluster-specific DEGs. Violin plots show the distribution of average expression levels of these DEGs across all clusters. (c) Cell type annotation using the PanglaoDB marker database.

(a) Top 10 DE Genes for Each Cluster Identified by CAKE in Mouse Brain Data

Cluster 0	Cluster 1	Cluster 2	Cluster 3	Cluster 4
Mia	Gad1	Pf4	Hexb	Hexb
Dynlrb2	Gad2	Mrc1	Ctss	Cx3cr1
Calml4	Slc6a1	Csf1r	P2ry12	Ctss
1110017D15Rik	Vstm2a	Tyrobp	Csf1r	Csf1r
1700094D03Rik	Dner	Ms4a7	Laptm5	P2ry12
Rsph1	Dlx6os1	Lyz2	C1qa	Gpr34
Clu	Igf1	Cbr2	Tyrobp	C1qb
Fam183b	Arl4c	Sepp1	Cx3cr1	Laptm5
Tmem212	Cnr1	Stab1	Ccl3	Siglech
Ccdc153	Resp18	Fcgr3	Cd53	Fcrls

Cluster 5	Cluster 6	Cluster 7	Cluster 8
Apoe	Lamp5	Clu	Palmd
Gja1	Pcp4	Slc1a2	Wfs1
Pla2g7	Tpm1	Ntsr2	Itm2a
Mt1	Ier5	Prdx6	Slc16a1
Slc1a3	Myo1b	Aqp4	Ly6c1
Ppap2b	Tnnc1	Aldoc	Nfasc
Atp1a2	Mylk	Mt1	Ptprb
Gpr37l1	Mustn1	Pla2g7	Cldn5
Mt2	Igfbp5	Slc1a3	Gjc3
Slc4a4	Myl4	Gja1	Mag

(c) Top Cell Type Matches for Each Cluster Identified by CAKE Based on PanglaoDB Markers

Cluster	Match Type	Annotation	Score
0	Primary	Ciliated cells	0.1111
	Secondary	Ependymal cells	0.1017
1	Primary	GABAergic neurons	0.2000
2	Primary	Microglia	0.0506
3	Primary	Microglia	0.0759
4	Primary	Microglia	0.1013
5	Primary	Bergmann glia	0.0976
	Secondary	Astrocytes	0.0952
6	Primary	No annotation	–
7	Primary	Bergmann glia	0.1220
	Secondary	Astrocytes	0.1111
8	Primary	No annotation	–

(b) Average Expression of Top 10 DE Genes from Each Cluster Found by CAKE

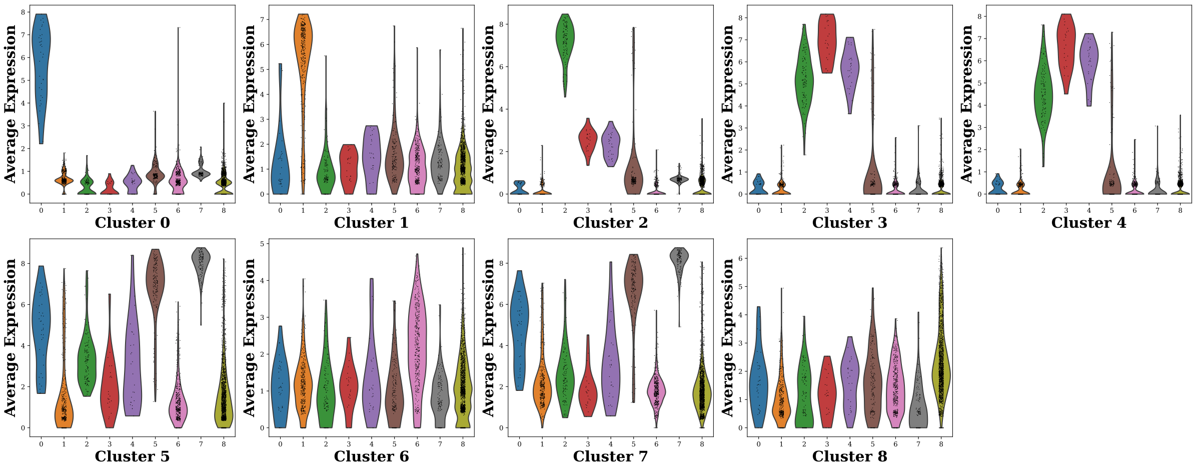

Figure S28: Clustering Performance of CAKE on the Mouse Brain Dataset and Cell Type Annotation using DEGs and Canonical Markers. (a) Top 10 DEGs identified for each cluster by CAKE. (b) Across-cluster average expression of cluster-specific DEGs. Violin plots show the distribution of average expression levels of these DEGs across all clusters. (c) Cell type annotation using the PanglaoDB marker database.

(a) Top 10 DE Genes for Each Cluster Identified by scASDC in Mouse Brain Data

Cluster 0	Cluster 1	Cluster 2	Cluster 3	Cluster 4
Tpm1	Wfs1	Ctss	Tm4sf1	Mag
Myl9	Nnat	Csf1r	Epas1	Gjc3
Acta2	Nr4a2	Rnase4	Ptprb	Ptgds
Flna	Tpm1	Tyrobp	Ly6c1	Nfasc
Crip1	Ncan	Laptm5	Sparc	Fa2h
Tagln	Rgs2	C1qa	Itm2a	Hapln2
Cald1	Resp18	C1qb	Esam	Serpinb1a
Tpm2	Dcn	Cx3cr1	Ackr3	S100b
Mgp	Cnr1	Fcrls	Myl9	S100a1
Myh11	Ier5	Fcgr3	Gpr116	S100a6

Cluster 5	Cluster 6	Cluster 7	Cluster 8
Slc1a3	Itm2a	Pf4	Gad2
Gja1	Sparc	Mrc1	Gad1
Ppap2b	Ly6c1	Ctsc	Vstm2a
Clu	Cldn5	Tyrobp	Slc6a1
Slc1a2	Ptprb	Csf1r	Dner
Gpr37l1	Pltp	C1qb	Arl4c
Apoe	Slco1a4	C1qa	Dlx6os1
Atp1a2	Slco1c1	Lyz2	Igf1
Slc4a4	Flt1	Sepp1	Resp18
Mt2	Abcb1a	Fcgr3	Cnr1

(c) Top Cell Type Matches for Each Cluster Identified by scASDC Based on PanglaoDB Markers

Cluster	Match Type	Annotation	Score
0	Primary	Myofibroblasts	0.4444
	Secondary	Vascular smooth muscle cells	0.3333
1	Primary	Meningeal cells	0.1111
	Secondary	Dopaminergic neurons	0.0500
2	Primary	Microglia	0.0759
	Secondary	Kupffer cells	0.0652
3	Primary	Myofibroblasts	0.1111
	Secondary	Pancreatic progenitor cells	0.0667
4	Primary	Nuocytes	0.0833
	Secondary	Enteric glia cells	0.0588
5	Primary	Bergmann glia	0.1220
	Secondary	Glutaminergic neurons	0.1000
6	Primary	No annotation	–
7	Primary	Kupffer cells	0.0652
	Secondary	Microglia	0.0506
8	Primary	GABAergic neurons	0.2000

(b) Average Expression of Top 10 DE Genes from Each Cluster Found by scASDC

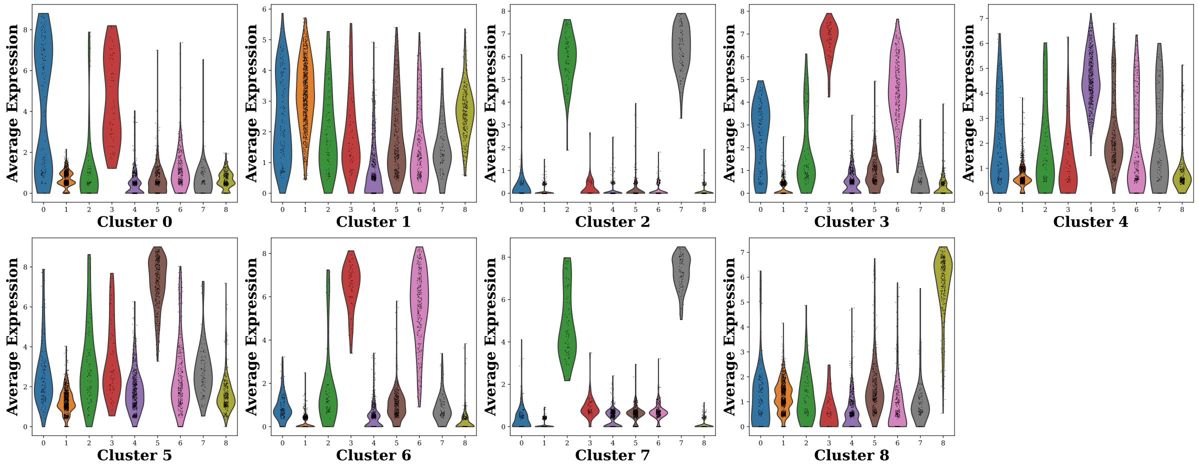

Figure S29: Clustering Performance of scASDC on the Mouse Brain Dataset and Cell Type Annotation using DEGs and Canonical Markers. (a) Top 10 DEGs identified for each cluster by scASDC. (b) Across-cluster average expression of cluster-specific DEGs. Violin plots show the distribution of average expression levels of these DEGs across all clusters. (c) Cell type annotation using the PanglaoDB marker database.

Figure/Table/Section	Description and Value
Experiments on simulated scRNA-seq data across modularity levels (Fig. 4 for average genes per cell type plots)	Number of cells = 3000<br>Number of genes = 1000<br>Number of embedded modules = 30<br>Average number of cells per module = 38<br>Target density of the gene expression matrix = 0.03<br>Density within modules = 0.3<br>Density between modules = 0.1<br>Probability of inter-module connections = 0.6<br>Average background expression = 10<br>Average within-module expression = 20<br>Average inter-module expression = 10<br>Workers = 30<br>Walk length = 30<br>Embedding dimensions = 30<br>Preference exponent = 50<br>Average number of genes per module $\in \{10, 20, 30, 40, 70, 80, 90, 100\}$
Experiments on simulated scRNA-seq data across modularity levels (Fig. 4 for number of modules plots)	Number of embedded modules $\in \{10, 20, 30, 40, 70, 80, 90, 100\}$<br>Average number of genes per module = 50<br>Average number of cells per module = (# genes / # modules) +3<br>Other parameters are the same as Fig. 4 for average genes per cell type plots.
Experiments on the impact of module sizes on ARI clustering performance (Fig. 5)	Same as in Fig. 4 for average genes per cell type plots.
Experiments on the impact of module counts on ARI clustering performance (Fig. 6)	Same as in Fig. 4 for number of modules.
Comparison of ARI and NMI across clustering methods for the ScMixology benchmark datasets (Table IV)	Number of highly expressed genes used = 500<br>Preference exponent = 10<br>Workers = 50<br>Walk length = 10<br>Embedding dimensions = 10
Experiments on human pancreas (Section III F)	Number of highly expressed genes used = 1000<br>Number of clusters = 9<br>Preference exponent = 10<br>Workers = 100<br>Walk length = 50<br>Embedding dimensions = 10
Experiments on mouse pancreas (Section S7)	Number of clusters = 13<br>Other parameters are the same as for human pancreas analysis (Section III F).
Experiments on human brain (Section S6)	Number of clusters = 7<br>Other parameters are the same as for human pancreas analysis (Section III F).
Experiments on mouse brain (Section S7)	Number of clusters = 9<br>Other parameters are the same as for human pancreas analysis (Section III F).

Table S4: Hyperparameters Used in Each Experiment.

References

[Barber, 2007] Barber, M. J. (2007). Modularity and community detection in bipartite networks. *Physical Review E—Statistical, Nonlinear, and Soft Matter Physics*, 76(6):066102.

[Chitra and Raphael, 2019] Chitra, U. and Raphael, B. J. (2019). Random walks on hypergraphs with edge-dependent vertex weights. In Chaudhuri, K. and Salakhutdinov, R., editors, *Proceedings of the 36th International Conference on Machine Learning, ICML 2019, 9-15 June 2019, Long Beach, California, USA*, volume 97 of *Proceedings of Machine Learning Research*, pages 1172–1181, Long Beach, California, USA. PMLR.

[Ciortan and Defrance, 2022] Ciortan, M. and Defrance, M. (2022). Gnn-based embedding for clustering scrna-seq data. *Bioinformatics*, 38(4):1037–1044.
